# MMP-cleavable linker platform for tumour-responsive homo- and heterobivalent antibody–drug conjugates

**DOI:** 10.1039/d5sc06664f

**Published:** 2025-12-03

**Authors:** Andrew J. Counsell, Stephen J. Walsh, Nicola Ashman, Mahri Park, Friederike M. Dannheim, Thomas A. King, Thomas Wharton, Jason S. Carroll, David R. Spring

**Affiliations:** a Yusuf Hamied Department of Chemistry, University of Cambridge Lensfield Road Cambridge CB2 1EW UK spring@ch.cam.ac.uk; b Cancer Research UK Cambridge Institute Robinson Way Cambridge CB2 ORE UK

## Abstract

Herein we report the development of a novel linker platform for tumour-responsive antibody–drug conjugates. A series of functionalised homo- and heterobivalent BisFab conjugates have been synthesised, comprising an MMP-cleavable peptide sequence which facilitates selective monomerisation in the tumour microenvironment of the BisFab into two smaller cytotoxic species. The platform was then expanded to produce bispecific BisFab conjugates.

## Abstract 2

Herein we report the development of a novel linker platform for tumour-responsive antibody–drug conjugates.

## Graphical abstract

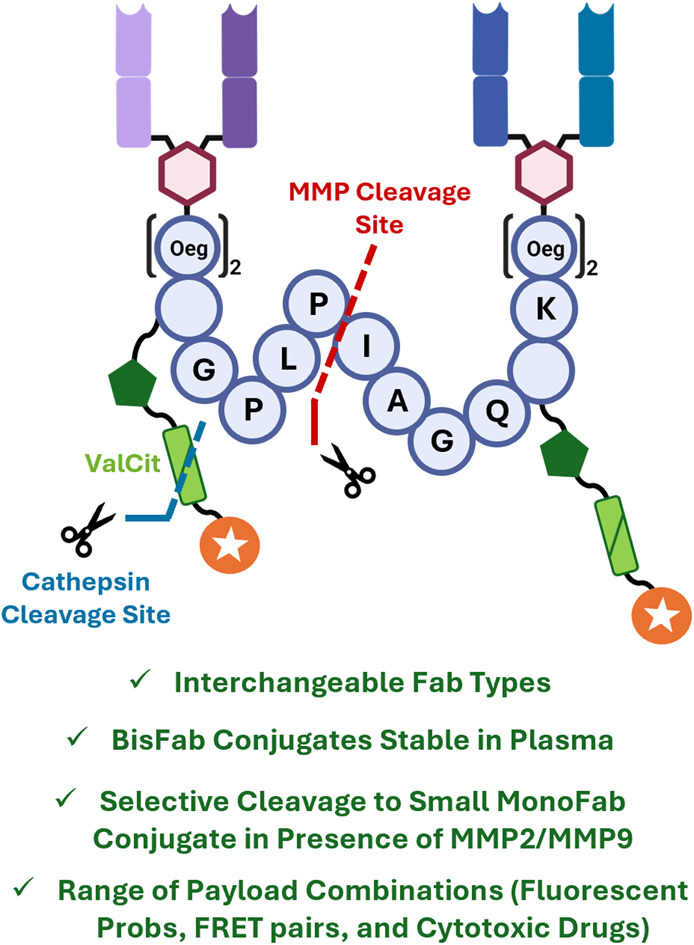

## Introduction

Antibody-based conjugates are of prominent interest to the pharmaceutical industry, due to their ability to attenuate the systemic cytotoxicity of small molecule drugs until their accumulation in tumours. To date, most development has focused on conjugates comprising full sized IgG antibodies, which face limitations in terms of tumour penetration on account of their large size (c. 150 kDa).^1–3^ In contrast, conjugates comprising smaller antibody fragments, such as antibody-binding fragments (Fabs, c. 50 kDa) or single-chain variable fragments (scFvs, c. 25 kDa), elicit potential for increased tumour penetration. This provides benefits particularly in the context of solid tumours, including a greater and ‘more homogenous’ tumour uptake, ultimately leading to a widened therapeutic window.^4–11^ However, smaller therapeutic scaffolds suffer from limitations of their own and typically exhibit decreased circulatory time and therefore reduced tumour exposure.^8,9^ To attain a balance between the two extremes, a plethora of strategies have been reported that enable the conjugation of two Fab regions to produce homodimer and heterodimer ‘BisFab’ constructs which are larger than Fabs yet smaller than full length antibodies.^12–20^

We believe that it may be possible to further improve this concept to maximise the extent of tumour penetration without the expense of decreased circulation, by exploiting extracellular proteolytic activity to effect controlled fragmentation of a therapeutic comprising two antibody fragments. As such, we designed a cleavable BisFab format, wherein two antibody Fab subunits are connected by a linker incorporating the matrix metalloproteinase (MMP)-cleavable motif Gly-Pro-Leu-Gly-Ile-Ala-Gly-Gln. This linker is obtainable by solid-phase peptide synthesis (SPPS), and easily adapted to facilitate payload or fluorophore conjugation.

MMPs are extracellular proteins which are involved in the natural degradation of the extracellular matrix and have been shown to be upregulated in some cancers (*e.g.* MMP2/MMP9).^21–23^ MMP-cleavable moieties have been incorporated into therapeutics that have been shown to exhibit alterations in affinity, structure, conformation, hydrophobicity, and charge, with a consequential positive influence on pharmacokinetic profile in terms of tumour-selective accumulation, tissue penetration, selective drug release, cellular uptake, and intracellular targeting.^24–27^

By connecting the antibody fragments through a tumour-microenvironment-cleavable linker, the intention is that the conjugate is able to benefit from increased stability and circulation time, all the while being primed for dissociation into smaller components upon reaching the tumour microenvironment for improved penetration.

**Fig. 1:**
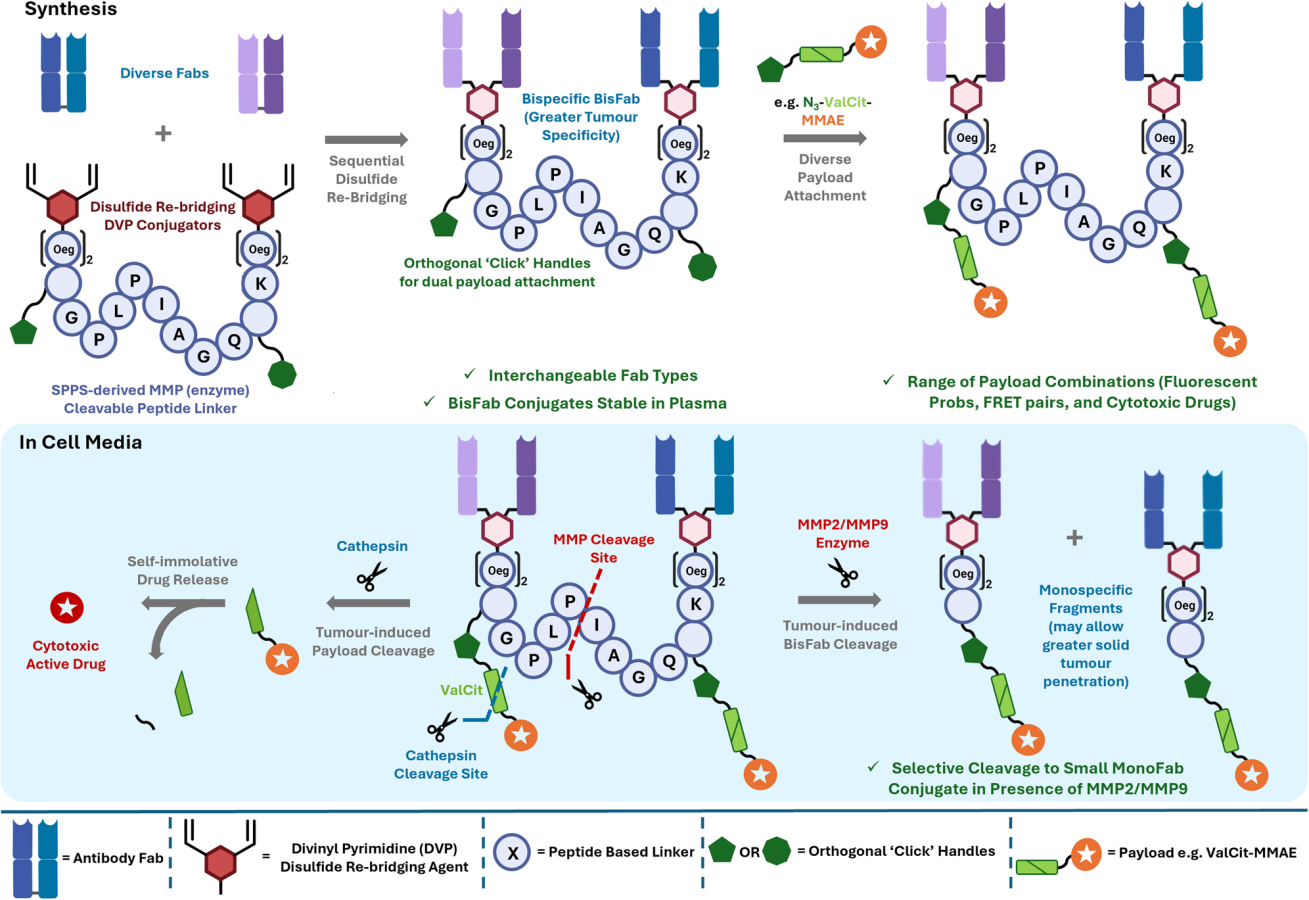
Overview of the cleavable BisFab concept. Sequential disulfide re-bridging of two Fab units to a peptide linker creates a BisFab construct that can be functionalised with payloads-of-interest *via* ‘click’ chemistry. The full conjugate is stable in plasma but will fragment in the presence of MMPs to release two smaller monospecific conjugates. If a selective drug release is desired, self-immolative linker systems can be incorporated.

Here we report the synthesis of MMP-cleavable, mono- and bispecific BisFabs and demonstrate their selectivity and potency in cellular assays (Fig. 1). Late-stage functionalisation of BisFabs using click handles allowed the bioorthogonal incorporation of the fluorophore Alexa Fluor® 488 (AF488), cytotoxin monomethyl auristatin E (MMAE), and a tetramethylrhodamine (TAMRA)/AF488 fluorescence resonance energy transfer (FRET) pair.

## Results and discussion

### Confirming MMP-dependent cleavage of a BisFab

**Fig. 2:**
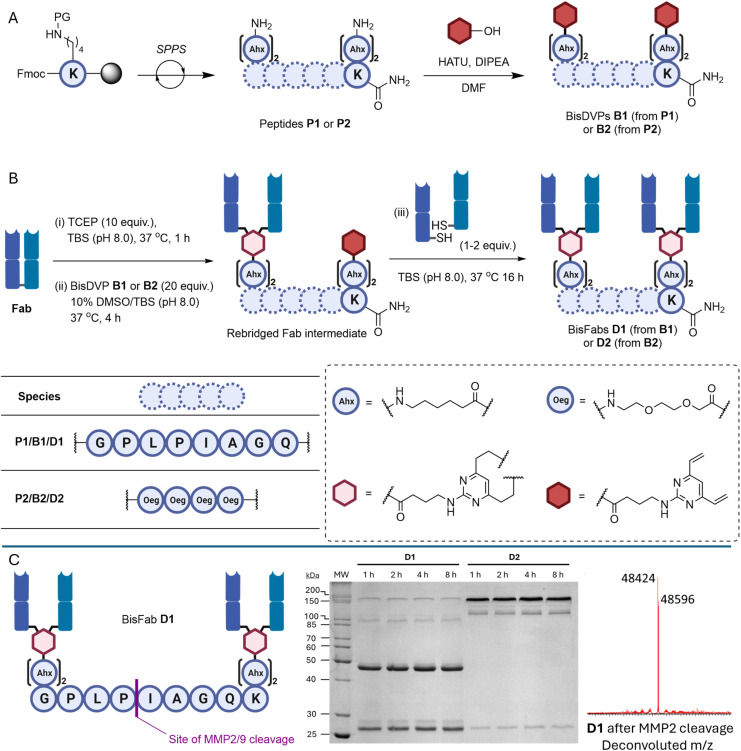
Synthetic approach towards unfunctionalised BisFabs: (A) two-stage synthesis of BisDVP linkers B1 and B2 comprising SPPS (solid phase peptide synthesis) and HATU-mediated solution phase DVP coupling; (B) trastuzumab Fab dimerisation with BisDVP linkers to produce BisFabs D1 and D2, respectively; (C) cleavage studies using MMP2. (Left) Scheme of D1 showing MMP2/9 cleavage site. BisFab D1 showed full cleavage with MMP2. D1 or D2 (5 µM) were incubated at 37 °C with 0.01 equivalents of MMP2 in TCN buffer (50 mM Tris, 10 mM CaCl_2_, 150 mM NaCl, pH 7.5) over 8 hours, with aliquots taken at 1, 2, 4 and 8 hours; (Middle) SDS-PAGE analysis from BisFab D1 and D2 cleavage studies with MMP2; lanes: MW = molecular weight marker, time points indicated above lane marker; (Right) deconvoluted MS of Fab monomer species produced upon MMP2-mediated cleavage of D1: observed 48 424 Da and 48 596 Da, expected 48 421 Da and 48 594 Da. PG = Alloc, X = variable letter, Oeg = 8-amino-3,6-dioxaoctanoic acid, Ahx = 6-aminohexanoic acid.

To confirm that MMP can cleave a peptide in a BisFab conjugate, proof-of-concept conjugates with and without the MMP-cleavable peptide Gly-Pro-Leu-Gly-Ile-Ala-Gly-Gln were designed. A branched SPPS strategy was used to synthesise the linkers (Fig. 2A). Following synthesis of the backbone, which consisted of either the MMP-cleavable peptide (P1) or poly(ethylene glycol) (P2), flanked by protected lysine residues, the lysine side chains were deprotected and SPPS continued to extend the linker with short alkyl (6-aminohexanoic acid; Ahx) spacers. The branched peptide was cleaved from the resin, and solution phase amide coupling with divinylpyrimidine (DVP) moieties^28^ provided BisDVP linkers B1 and B2.

BisFab conjugates D1 and D2 were then generated using a two-step strategy (Fig. 2B). Firstly, an excess of BisDVP linker was used to rebridge trastuzumab Fab following disulfide reduction. Unreacted BisDVP was removed by ultrafiltration and then another equivalent of reduced trastuzumab Fab was added to install the second Fab on the conjugate. Although attempts were made to form the homodimer in a single conjugation without success, this two-step strategy provided efficient access to the homodimer products after size exclusion chromatography (SEC) to remove unreacted Fab and monofunctionalised intermediates (Fig. S2). SDS-PAGE analysis of the SEC-purified BisFab suggested the presence of a small quantity of incomplete re-bridged species. As non-reducing SEC analysis showed the conjugate to be clean, the presence of lighter species on the reducing SDS-PAGE gel is attributed to some of the light chain units being non-covalently bonded to the corresponding heavy chains in a Fab. This could occur if the DVP conjugator reacted successfully with the heavy chain but not with the light. In the non-reducing conditions of the SEC, non-covalent interactions hold the BisFab together thus making it appear the same as the fully re-bridged version. As all cysteine conjugation systems that do not re-bridge disulfides (such as maleimide) generate antibody constructs without the covalent disulfide bridges intact, the presence of non-covalently held light chain should not impact the properties of the BisFab construct in physiological settings. Changing the length of the Ahx linkage between 1–4 units was not observed to impact conjugation efficiency and so linkers with two such units were selected for further synthesis and studies (Fig. S2b).

Pleasingly, cleavage studies with activated MMP2 rapidly monomerised BisFab D1 (Fig. 2C) whereas non-cleavable BisFab D2 was found to remain intact under the same conditions. Gratifyingly, this consumption was observed to go to near completion for BisFab D1 within 1 hour with 0.01 equivalents of MMP2. A significant amount of cleavage was also observed with 0.001 equivalents of MMP2 (Fig. S3). In all cases, no additional consumption of the dimer species was detected beyond 1 hour. Protein LCMS analysis confirmed cleavage had occurred to completion (with no BisFab detectable), and that cleavage occurred at the expected Gly–Ile bond.

### Homo- and heterobifunctional BisFabs

With the MMP2 cleavage concept proven, attention turned to functionalising the BisFab conjugates with model payloads. It was envisaged that if two payloads were placed either side of the MMP cleavage site, then both fragments would contain an active payload after monomerisation. Firstly, by incorporating two identical handles, homobifunctional BisFabs could be produced. These species would ensure uniform behaviour of the BisFab cleavage products, desirable for fluorescent labelling or antibody–drug conjugate formation.

**Fig. 3:**
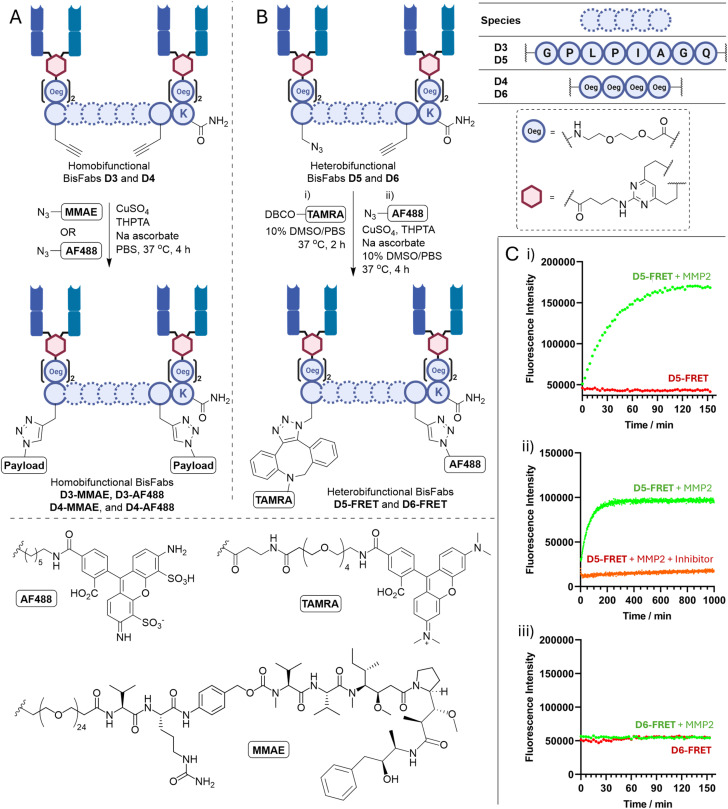
Homo- and heterobifunctional BisFabs. (A) Cleavable D3 and non-cleavable D4 BisFabs (synthesised *via* SPPS, DVP coupling, and bioconjugation) were reacted with AF488 or MMAE *via* click chemistry to generate homobifunctional BisFabs D3-MMAE, D3-AF488, D4-MMAE, or D4-AF488; (B) cleavable D5 and non-cleavable D6 BisFabs (synthesised *via* SPPS, DVP coupling, and bioconjugation) were reacted with the TAMRA and AF488 FRET pair *via* consecutive SPAAC and CuAAC to generate heterobifunctional BisFabs D5-FRET and D6-FRET; (C) fluorometric measurement of Alexa Fluor® 488 quenching in dimers D5-FRET and D6-FRET: (i) incubation of D5-FRET in the presence (green) and absence (red) of MMP2; (ii) incubation of D5-FRET with MMP2 in the presence (orange) and absence (green) of the MMP inhibitor (2*R*)-2-[(4-biphenylylsulfonyl)amino]-3-phenylpropionic acid; (iii) incubation of non-cleavable D6-FRET in the presence (green) or absence (orange) of MMP2. *λ*_ex_ = 495 nm, *λ*_em_ = 535 nm. Each point is the average of three technical replicates. THPTA = tris(3-hydroxypropyltriazolylmethyl)amine; MMAE = monomethyl auristatin E; AF488 = Alexa Fluor® 488; DBCO = dibenzylcyclooctyne; TAMRA = carboxytetramethylrhodamine; Oeg = 8-amino-3,6-dioxaoctanoic acid.

SPPS was used to synthesise MMP-cleavable and non-cleavable BisFabs D3 and D4, respectively, bearing alkyne handles (Fig. 3A). An alternative lysine protecting group (Dde, *N*-(1-(4,4-dimethyl-2,6-dioxocyclo-hexylidene)-ethyl)) was used to overcome issues experienced with Pd-catalysed Alloc removal, which were suspected to be due in part to the presence of the free alkynes.^29^ 8-Amino-3,6-dioxaoctanoic acid (Oeg) was selected over 6-aminohexanoic acid (Ahx) as branch spacers in order to improve the solubility of the resulting linkers. After the peptides (P3 and P4) were cleaved from the resin, coupling to DVP provided linkers B3 and B4 (see SI). The corresponding BisFabs D3 and D4 were then generated using the previously optimised two-step dimerisation procedure.

Pleasingly, copper-catalysed azide alkyne cycloaddition (CuAAC) enabled installation of a fluorophore or cytotoxin (Fig. 3A). Alexa Fluor® 488 was incorporated to produce BisFabs D3-AF488 and D4-AF488 with a fluorophore-to-antibody ratio (FAR) of 2.0 (Fig. S5), whilst reaction with a payload containing the tubulin inhibitor MMAE produced BisFabs D3-MMAE and D4-MMAE with drug-to-antibody ratios (DARs) of 1.8 and 1.9, respectively (Fig. S6). The MMAE linkage contains a cathepsin cleavable valine-citrulline dipeptide commonly used in antibody–drug conjugates (ADCs) to allow the potent drug to be selectively released at the tumour site.

Alternatively, it was envisaged that incorporating two different handles across the cleavage site would enable differential functionalisation. Such heterobifunctional BisFabs could be useful for the incorporation of synergistic payloads in next-generation therapeutics or to introduce FRET pairs that would become fluorescently active upon cleavage for imaging applications.

Therefore, BisFabs D5 and D6 were synthesised bearing both an azide and an alkyne handle *via* SPPS, DVP coupling, and bioconjugation (Fig. 3B).

To demonstrate the practicality of a heterobifunctional BisFab, cleavable and non-cleavable FRET pair containing BisFabs were synthesised. Consecutive strain promoted azide–alkyne cycloaddition (SPAAC), with a DBCO-TAMRA reagent, and CuAAC, with an Alexa Fluor® 488 azide, generated FRET-enabled BisFabs D5-FRET and D6-FRET (Fig. 3B).

A range of fluorescence cleavage assays were then performed on BisFabs D5-FRET and D6-FRET, from which MMP-mediated BisFab cleavage was monitored. Incubation of D5-FRET with either MMP2 (Fig. 3C) or MMP9 (Fig. S4) led to fluorescence recovery, indicating successful cleavage of the BisFab despite the additional steric hindrance near the cleavage site. As expected, minimal increase in fluorescence was observed when non-cleavable D6-FRET was incubated under the same conditions (Fig. 3C(iii)). When the known MMP inhibitor (2*R*)-2-[(4-biphenylylsulfonyl)amino]-3-phenylpropionic acid^30^ was present, no cleavage was observed (Fig. 3C(ii)).

### Stability and biological evaluation

With a range of conjugates synthesised, attention turned to examining conjugate stability and activity in comparison to the parent trastuzumab antibody. D3-AF488 and D4-AF488 were used to demonstrate the stability of BisFab conjugates. Premature release of cytotoxic molecules from antibody conjugates is likely to result in off-target toxicity and will usually occur at the site of protein conjugation. In the context of BisFabs, it is important that the linking peptide remains stable in circulation. Gratifyingly, no loss of BisFab fluorescence or transfer of fluorescence to other proteins was observed when D3-AF488 and D4-AF488 were incubated with human plasma at 37 °C over a 96-hour period (Fig. S8). Furthermore, no cleavage of the BisFabs was observed.

**Fig. 4:**
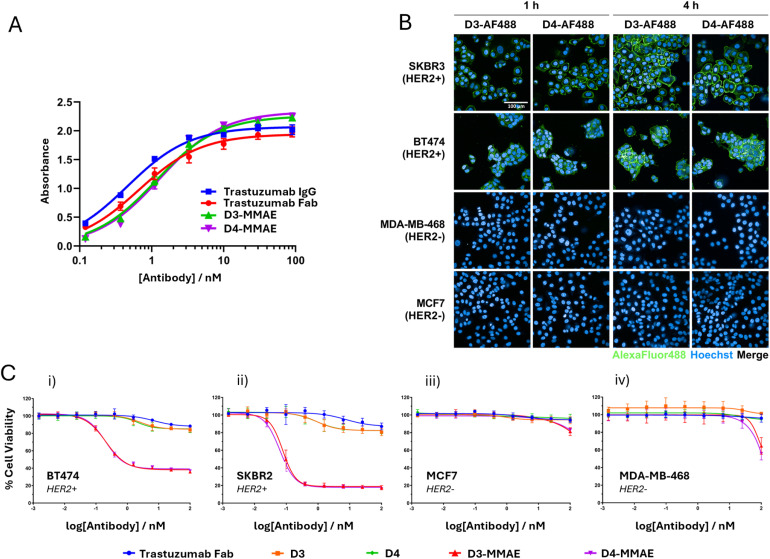
ELISA, microscopy and cytotoxicity data for the functionalised BisFabs. (A) ELISA binding affinity comparison between trastuzumab IgG, trastuzumab Fab, and conjugates D6-MMAE and D7-MMAE. All conjugates exhibited concentration-dependent binding to HER2. Error bars represent the standard deviation of three technical replicates; (B) live cell microscopy images of HER2-positive (SKBR3 and BT474) and HER2-negative (MDA-MB-468 and MCF7) cell lines following incubation for either 1 or 4 hours with fluorescent BisFabs D3-AF488 and D4-AF488. Scale bar represents 100 µm. Alexa Fluor® 488 and Hoechst channels have been merged in all images; (C) cell viability of HER2-positive (i) BT474 and (ii) SKBR3, and HER2-negative (iii) MCF7 and (iv) MDA-MB-468 cell lines, after 96-hour treatment with trastuzumab Fab, D3, D4, D3-MMAE, D4-MMAE. Data is reported as the mean of three biological replicates, and error bars represent the standard error of the mean.

Next, the binding affinities of conjugates D3-MMAE and D4-MMAE for the HER2 receptor were compared to those of trastuzumab IgG or trastuzumab Fab by indirect enzyme-linked immunosorbent assay (ELISA) (Fig. 4A). Gratifyingly, no substantial difference was observed across all species showing that dimerisation of trastuzumab Fab in the BisFab format and subsequent CuAAC functionalisation does not significantly interfere with recognition of the HER2 receptor.

The selectivity of the BisFab conjugates for HER2 was demonstrated by incubating AlexaFluor conjugates with a panel of HER2-positive and HER2-negative human breast cancer cell lines. Live cell fluorescent microscopy revealed fluorescent labelling of the cell surface in both HER2-positive cell lines (SKBR3 and BT474) after incubation for one hour with D3-AF488 or D4-AF488 (Fig. 4B). Moreover, significant internalisation was observed after four hours of incubation for the BT474 cell line. Conversely, no fluorescent labelling was detected in either of the HER2-negative cell lines (MDA-MB-468 and MCF7). The results confirmed the excellent selectivity of BisFab conjugates towards HER2-positive cells over HER2-negative cells, and further confirmed successful internalisation.

A cell viability assay was used to further demonstrate the selectivity and cytotoxicity of BisFab conjugates. Both D3-MMAE and D4-MMAE exhibited significant concentration-dependent cytotoxicity against HER2-positive cell lines (BT4747 and SKBR3) compared to both unmodified trastuzumab Fab, and unfunctionalised BisFabs D3 and D4 (Fig. 4C). D3-MMAE was found to have IC_50_ values of 209 pM and 78 pM against BT474 and SKBR3, respectively. Likewise, D4-MMAE was found to have corresponding IC_50_ values of 201 pM and 63 pM.

The higher activity against SKBR3 cells despite seemingly lower internalisation (Fig. 4B) may be due to difference in the time points in the assay readouts, with internalisation becoming sufficient to see high cytotoxicity after 96 hours. The sensitivity of the SKBR3 cell line to MMAE alone has also been shown to be higher than for BT474,^31^ which is concurrent with the data reported here.

Both ADCs exhibited no significant effect on cell proliferation for the HER2-negative cell lines when treated at concentrations below 100 nM. No significant difference in anti-proliferative effect towards HER2-positive cells in 2D culture was observed between D3-MMAE and D4-MMAE; further experiments will need to carried out to determine if the MMP-cleavable constructs offer additional benefits in 3D culture settings which more closely mimic solid tumour environments.

These results demonstrate the utility of the BisDVP/BisFab platform for the generation of potent ADCs.

### Bispecific fab dimers

Beyond the potential benefits in terms of tissue penetration, we considered the utility of BisFabs to generate bispecific antibody constructs. There are multiple benefits that arise from the tandem specificity of a bispecific antibody. For example, they can be tailored to address the reality that cancers present with a variety of antigen expression profiles,^32^ as well as achieve increased efficacy by simultaneous therapeutic action through multiple pathways (*e.g.* immune effector cell direction).^33^

**Fig. 5:**
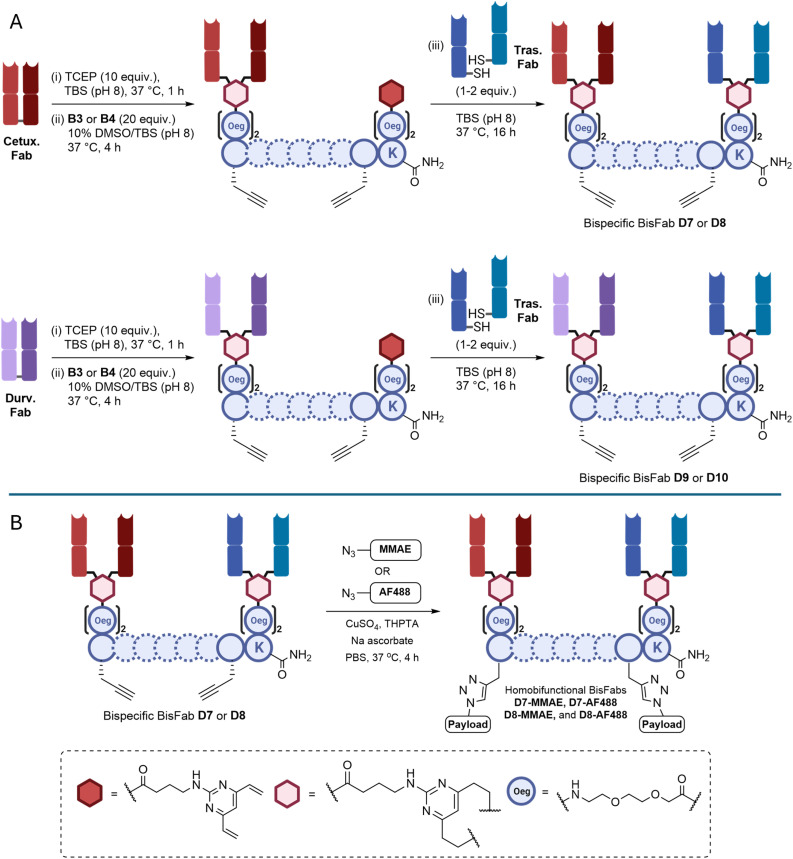
Bispecific BisFabs. (A) Synthesis of cleavable and non-cleavable bispecific BisFabs from cetuximab, durvalumab and trastuzumab Fabs; (B) click functionalisation of BisFabs D7 and D8 to with MMAE and AF488 to give D7-MMAE, D7-AF488, D8-MMAE, and D8-AF488. THPTA = Tris(3-hydroxypropyltriazolylmethyl)amine; MMAE = monomethyl auristatin E; AF488 = Alexa Fluor® 488.

Due to the stepwise synthesis of BisFabs using BisDVP linkers, the methodology is advantageous for generating bispecific antibody conjugates. To exemplify the versatility and modularity of the BisDVP platform, two bispecific combinations were envisaged—each with a distinct intended ‘bispecific advantage’ (Fig. 5A).

One advantage of bispecific conjugates is that of improved therapeutic selectivity, which can be achieved by simultaneously targeting two different receptors that are both expressed or upregulated by the desired target cell. In this case, presented by the desired cell, thus increasing the likelihood of targeting that cell over cells expressing only one of the antigens. Various studies have demonstrated the potential benefit in the simultaneous targeting of EGFR and HER2. Cancers such as bladder,^34,35^ colorectal,^36,37^ oesophageal^38^ and prostate^39,40^ are all known to exhibit combined upregulation of both antigens. Moreover, the co-expression of EGFR and HER2 in certain subtypes of breast cancer is heavily associated with poor patient prognosis.^41,42^ Indeed, in light of this, a number of α-EGFR × α-HER2 bispecific therapeutics have been developed.^43–47^ To demonstrate the ability to generate this style of bispecific, BisFabs D7 and D8, containing the α-HER2 trastuzumab Fab and the α-EGFR cetuximab Fab, were designed.

Another benefit of bispecific conjugates is through the activation of multiple therapeutic pathways. The PD-L1/PD-1 interaction is known to mediate various immune suppressive mechanisms, such as the inhibition of cytokine production and T cell proliferation.^48^ PD-L1 is normally expressed on a variety of immune cells as a mechanism of inhibiting auto-immunity, and is therefore a prime candidate target for enhancing selectivity towards tumour-antigen presenting cells.^49^ As has been recently remarked, simultaneous targeting of PD-L1 may offer a route towards the circumvention of acquired resistance to HER2-directed therapies on account of the association with trastuzumab-induced drug resistance and upregulation of PD-L1.^50^ Clinical studies in which both HER2 and PD-L1 were targeted with a combination of α-HER2/α-PD-L1 monoclonal antibodies revealed strong patient responses,^51,52^ and a recently-reported α-HER2 × α-PD-L1 bispecific exhibited greater therapeutic efficacy than single and combination antibody treatments in a HCC1954 xenograft model.^53^ To demonstrate the ability to generate this style of bispecific, BisFabs D9 and D10, containing the α-HER2 trastuzumab Fab and the α-PD-L1 durvalumab Fab, were designed.

The bispecific BisFabs were synthesised (Fig. 5A) and conjugated to payloads (Fig. 5B). The Fabs of the α-EGFR cetuximab (Erbitux®) and α-PD-L1 durvalumab (Imfinzi®) were synthesised from their respective parent IgGs. Each Fab was then reacted with homobifunctionalised BisDVP linkers L3 (cleavable) and L4 (non-cleavable), followed by SEC purification to remove unreacted linker and any unwanted homodimer that may have formed in the first reaction step. The intermediates were then subjected to reaction with trastuzumab Fab to yield the desired bispecific BisFabs. As the BisDVP linkers are asymmetric, the initial Fab conjugation may occur on ether end, which, after the second fab is added, will cause two isomers to be generated. Whilst these are not chemically identical, the difference in the pharmacology will be negligible. D7 and D8 were then functionalised with either Alexa Fluor® 488 or PEG_24_-Val-Cit-PABC-MMAE.

Bispecific BisFab conjugates D7-AF488 and D8-AF488 were shown to be stable in human plasma over four days, both with respect to fluorophore conjugation and linker peptide integrity (Fig. S9).

**Fig. 6:**
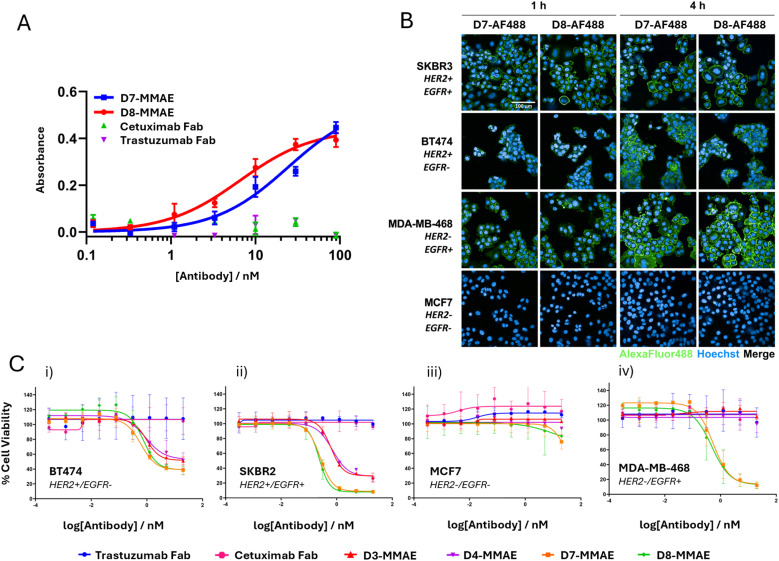
ELISA, microscopy and cytotoxicity of the bispecific BisFabs. (A) HER2 × EGFR sandwich ELISA analysis of trastuzumab Fab, cetuximab Fab, D7-MMAE and D8-MMAE. All α-HER2 × α-EGFR conjugates displayed concentration-dependent binding, whilst the monovalent controls exhibited no significant absorbance. Error bars represent the standard deviation of biological triplicates; (B) live cell microscopy images of SKBR3 (HER2-positive/EGFR-positive) and BT474 (HER2-positive/EGFR-negative) and MDA-MB-468 (HER2-negative/EGFR-positive) and MCF7 (HER2-negative/EGFR-negative) cell lines following incubation for either 1 or 4 h with fluorescent BisFabs D7-AF488 and D8-AF488. Scale bar represents 100 µm; (C) cell viability of (i) BT474 (HER2+/EGFR−), (ii) SKBR3 (HER2+/EGFR+), (iii) MCF7 (HER2-/EGFR−) and (iv) MDA-MB-468 (HER2−/EGFR+) cell lines after 96-hour treatment with trastuzumab Fab, cetuximab Fab, D3-MMAE, D4-MMAE, D7-MMAE, or D8-MMAE. Data is reported as the mean of three independent biological replicates, and error bars represent the standard error of the mean.

To determine whether the α-EGFR × α-HER2 and α-PD-L1 × α-HER2 BisFabs were capable of simultaneous engagement of both target antigens, a sandwich ELISA was performed. Gratifyingly, simultaneous receptor engagement was observed for all BisFabs tested (Fig. 6A). None of the monovalent controls (trastuzumab Fab, cetuximab Fab or durvalumab Fab) exhibited significant absorbance in the assay.

Cellular selectivity of the α-EGFR × α-HER2 BisFabs D7-AF488 and D8-AF488 was then examined by live cell fluorescent microscopy (Fig. 6B). The same four cell-line panel as before (SKBR3, BT474, MDA-MB-468 and MCF7) was selected for incubation with the fluorescent bispecific conjugates. As was observed for the α-HER2 × α-HER2 conjugates, fluorescent labelling of the HER2-positive cell lines SKBR3 and BT474 was observed as expected in the case of both D7-AF488 and D8-AF488. Similarly, no fluorescent labelling was observed for the HER2-negative/EGFR-negative MCF7 cell line. Pleasingly, significant fluorescent labelling of the HER2-negative/EGFR-positive MDA-MB-468 cell line was observed after one hour, confirming the ability for binding of the bispecific BisFabs by means of the EGFR receptor. Moreover, in all cell lines (with the exception of MCF7) significant internalisation was observed after four hours of incubation.

The cytotoxicity of α-EGFR × α-HER2 MMAE conjugates D7-MMAE and D8-MMAE was then investigated across two HER2-positive cell lines (BT474 and SKBR3), and two HER2-negative cell lines (MCF7 and MDA-MB-468). Pleasingly, both α-EGFR × α-HER2 MMAE conjugates D7-MMAE and D8-MMAE exhibited significant concentration-dependent cytotoxicity compared to their unmodified antibody components, trastuzumab Fab and cetuximab Fab (Fig. 6C). Selectivity towards the HER2-positive and EGFR-positive cell lines was clearly confirmed, with both D7-MMAE and D8-MMAE exhibiting no significant effect on cell proliferation in the HER2-negative/EGFR-negative MCF7 cell line compared to the vehicle control. Notably, significant differences in the anti-proliferative activities of the α-EGFR × α-HER2 and α-HER2 × α-HER2 conjugates were observed in the SKBR3 (HER2-positive/EGFR-positive) and MDA-MB-468 (HER2-negative/EGFR-positive) cell lines. For the SKBR3 cell line, increased potency and efficacy was observed for the bispecific α-EGFR × α-HER2 conjugates (IC_50_ values of 0.25 and 0.23 nM, respectively) compared to that of the α-HER2 × α-HER2 conjugates (IC_50_ values of 0.61 and 0.70 nM, respectively), presumably owing to the dual antigen expression of the SKBR3 cell line facilitating greater cellular uptake. As expected, the α-EGFR × α-HER2 conjugates exhibited significant cytotoxicity against MDA-MB-468 (IC_50_ of 0.54 and 0.48 nM, respectively), with no corresponding cytotoxicity observed for the α-HER2 × α-HER2 conjugates. No significant difference in cell viability was observed in BT474 (HER2-positive/EGFR-negative) and MCF7 (HER2-negative/EFGR-negative) cell lines between the bispecific and monospecific ADCs.

## Conclusions

A novel platform for the generation of functionalised antibody-based conjugates has been developed. The platform is versatile, enabling the synthesis of homo- and hetero-bifunctional conjugates as well as the ability to generate bispecific conjugates. The exquisite selectivity and potent cytotoxicity of the reported conjugates affirm the potential for use of this platform in the development of therapeutics against cancer subtypes with multiple antigen expression.

Furthermore, by connecting two antibody Fab subunits with a tumour microenvironment-cleavable peptidic linker, the conjugates are primed for exploiting the benefits of both ‘large’ and ‘small’ biologics – improved circulatory half-life and greater tumour penetration. Future work will focus on exploring these properties *in vivo* to validate the concept.

## Author contributions

A. J. C. and S. J. W. were involved in conceptualisation and investigation. N. A., M. P., and F. M. D. were involved in investigation. J. S. C. and D. R. S. were involved in supervision. The manuscript was written through contributions of all authors. All authors have given approval to the final version of the manuscript.

## Conflicts of interest

There are no conflicts to declare.

## Supplementary Material

SC-OLF-D5SC06664F-s001

## Data Availability

The data supporting this article have been included as part of the supplementary information (SI). Supplementary information is available. See DOI: https://doi.org/10.1039/d5sc06664f.
