# Supplementary material for: MMP-cleavable linker platform for tumour-responsive homo- and heterobivalent antibody–drug conjugates
Source: Chem Sci. 2025 Dec 3;17(5):2722–31. doi: 10.1039/d5sc06664f (PMC12694610; doi:10.1039/d5sc06664f)
Supplement: SC-017-D5SC06664F-s001 [file SC-017-D5SC06664F-s001.pdf]

Supplementary Information (SI) for Chemical Science.
This journal is © The Royal Society of Chemistry 2025

Supplementary Information

MMP-Cleavable Linker Platform for Tumour-Responsive Homo- and Heterobivalent Antibody-Drug Conjugates

Andrew J. Counsell,[a] Stephen J. Walsh,[ab] Nicola Ashman,[a] Mahri Park,[a] Friederike M. Dannheim,[a] Thomas A. King,[a] Thomas Wharton, [a] Jason S. Carroll,[b] and David R. Spring*[a]

[a] *Yusuf Hamied Department of Chemistry, University of Cambridge, Lensfield Road, Cambridge, CB2 1EW, UK. E-mail: spring@ch.cam.ac.uk.*
[b] *Cancer Research UK Cambridge Institute, Robinson Way, Cambridge, CB2 ORE, UK.*

Contents

Experimental Procedures 2
- 1.1: Small Molecule General Information 2
- 1.2 Small Molecule Synthesis 3
- 1.3 Peptide Synthesis 8
  - Manual SPPS 8
  - Automated SPPS 8
- 1.4 BisDVP Linker Synthesis 10
- 1.5 Protein Chemistry 13
- 1.6 MMP2 Cleavage 29
- 1.7 Fluorimetry and DAR analysis 30
- 1.8 BisFab Plasma Stability 32
- 1.9 Enzyme-linked immunosorbent assay (ELISA) 34
- 1.10 Cell-based assays 35

Analytical Data 37
- NMR Spectra 37
- HPLC Traces 44
- Protein LCMS 46
- Bioconjugation Analyses 56

References 59

Experimental Procedures

1.1: Small Molecule General Information

All solvents and reagents were used as received unless otherwise stated. Ethyl acetate, methanol, dichloromethane, and acetonitrile were distilled from calcium hydride. Diethyl ether was distilled from a mixture of lithium aluminium hydride and calcium hydride. Petroleum ether refers to the fraction obtained between 40–60 °C upon distillation. Tetrahydrofuran was dried using Na wire and distilled from a mixture of lithium aluminium hydride and calcium hydride with triphenylmethane as indicator.

Non-aqueous reactions were conducted under a stream of dry nitrogen using oven-dried glassware. Temperatures of 0 °C were maintained using an ice-water bath. All temperatures below 0 °C were maintained using an acetone-dry ice bath. Room temperature refers to ambient temperature.

Manual flash column chromatography was carried out using slurry-packed Merck 9385 Kieselgel 60 SiO_2 (230-400 mesh) under a positive pressure of nitrogen. Reverse phase chromatography was performed on a Combiflash Rf-200 automated chromatography system with RediSep® reverse-phase C18-silica flash columns (20-40 µm).

Yields refer to spectroscopically and chromatographically pure compounds unless otherwise stated. Reactions were monitored by thin layer chromatography (TLC) or liquid chromatography mass spectroscopy (LCMS). TLC was performed using glass plates pre-coated with Merck silica gel 60 F_{254} and visualised by quenching of UV fluorescence (λ_{max} = 254 nm) or by staining with potassium permanganate. Retention factors (R_f) are quoted to two decimal places. LCMS was carried out using a Waters ACQUITY H-Class UPLC with an ESCi Multi-Mode Ionisation Waters SQ Detector 2 spectrometer using MassLynx 4.1 or MassLynx 4.2 software. ESI refers to the electrospray ionisation technique. LCMS solvents: A: 2 mM NH_4OAc in H_2O/MeCN (95:5); B: MeCN; and C: 2% formic acid. LCMS Column: ACQUITY UPLC® CSH C18 (2.1 mm × 50 mm, 1.7 µm, 130 Å) temperature controlled at 40 °C. LCMS solvent gradient: 5 – 95% B with constant 5% C over 1 min at a flow rate of 0.6 mL/min. LCMS detector: PDA eλ Detector 220 – 800 nm, interval 1.2 nm.

High resolution mass spectrometry (HRMS) measurements were recorded with a Micromass Q-TOF mass spectrometer or a Waters LCT Premier Time of Flight mass spectrometer. Mass values are reported within the error limits of ±5 ppm mass units.

Analytical high performance liquid chromatography (HPLC) was performed on an Agilent 1260 Infinity using a SUPELCOSIL ABZ+Plus column (150 mm × 4.6 mm, 3 µm), eluting with a linear gradient system (Solvent A: 0.05% (v/v) TFA in H_2O; Solvent B: 0.05% (v/v) TFA in CH_3CN) over 15 min in a flow rate of 1 mL/min. UV absorbance was monitored at 220 and 254 nm. Preparative high performance liquid chromatography (HPLC) was performed on an Agilent 1260 Infinity using a SUPELCOSIL ABZ+Plus column (250 mm × 21.2 mm, 5

μm), eluting with a linear gradient system (Solvent A: 0.05% (v/v) TFA in H_2O; Solvent B: 0.05% (v/v) TFA in CH_3CN). UV absorbance was monitored at 220 and 254 nm.

Proton and carbon nuclear magnetic resonance (NMR) were recorded using an internal deuterium lock on Bruker DPX-400 (400 MHz, 101 MHz), Bruker Avance 400 QNP (400 MHz, 101 MHz) and Bruker Avance 500 Cryo Ultrashield (500 MHz, 126 MHz). In proton NMR, chemical shifts (δ) are reported in parts per million (ppm), to the nearest 0.01 ppm and are referenced to the residual non-deuterated solvent peak ($CHCl_3$: 7.26, DMSO: 2.50, CH_3OH: 3.31). Coupling constants (J) are reported in Hertz (Hz) to the nearest 0.1 Hz. Data are reported as follows: chemical shift, multiplicity (s = singlet; d = doublet; t = triplet; q = quartet; qn = quintet; sep = septet; m = multiplet; or as a combination of these, *e.g.* dd, dt), integration, coupling constant(s), and assignment (where determinable; shifts labelled alphanumerically *e.g.* H^a, H^b, C^f; C_Q refers to quaternary carbon). In carbon NMR, chemical shifts (δ) are quoted in ppm, to the nearest 0.1 ppm, and are referenced to the residual non-deuterated solvent peak ($CHCl_3$: 77.16, DMSO, 39.52, CH_3OH: 49.00).

IR spectra were recorded neat on a Perkin-Elmer Spectrum One spectrometer with internal referencing. Selected absorption maxima (ν_{max}) are reported in wavenumbers (cm^{-1}).

1.2 Small Molecule Synthesis

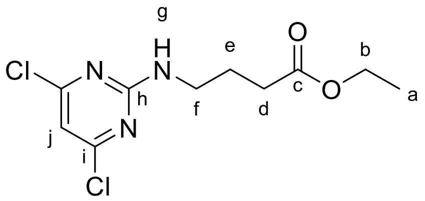

Ethyl 4-((4,6-dichloropyrimidin-2-yl)amino)butanoate (1)

To a solution of 2,4,6-trichloropyrimidine (2.00 mL, 17.4 mmol) and ethyl 4-aminobutyrate hydrochloride (3.20 g, 19.1 mmol) in acetone (60 mL) at 0 °C, Et_3N (5.6 mL, 34.5 mmol) was added dropwise. The mixture was stirred at 0 °C for 2 h. The solvent was then removed under reduced pressure, and the residue taken up in H_2O (20 mL) and extracted with CH_2Cl_2 (4 × 20 mL). The combined organic layers were dried over anhydrous $MgSO_4$, concentrated under reduced pressure, and purified by column chromatography (SiO_2, Petroleum Ether ramping to EtOAc/Petroleum Ether = 3:10) to yield **1** as a white solid (0.888 mg, 3.19 mmol, 18%).

R_f (SiO_2, EtOAc/Petroleum Ether = 1:5) 0.33. **^{1}H NMR** (400 MHz, $CDCl_3$) δ 6.56 (s, 1H, H^j), 6.08 (br s, 1H, H^g), 4.11 (q, 2H, *J* = 7.1 Hz, H^b), 3.46 (q, 2H, *J* = 6.6 Hz, H^f), 2.36 (t, 2H, *J* = 7.2 Hz, H^d), 1.90 (qn, 2H, *J* = 7.1 Hz, H^e), 1.23 (t, 3H, *J* = 7.2 Hz, H^a). **^{13}C NMR** (101 MHz,

$CDCl_3$) δ 173.2 (C^c), 161.9 (C^h + C^i), 108.9 (C^j), 60.6 (C^b), 40.9 (C^f), 31.6 (C^d), 24.6 (C^e), 14.3 (C^a). **LRMS** (ESI+) *m/z* found $[M+H]^+$ 278.2, $C_{10}H_{14}Cl_2N_3O_2^+$ required 278.0.

The data are consistent with previously published data.[1]

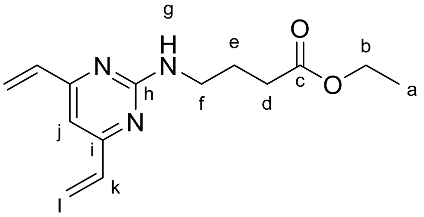

Ethyl 4-((4,6-divinylpyrimidin-2-yl)amino)butanoate (2)

A solution of **1** (220 mg, 0.79 mmol), potassium vinyltrifluoroborate (540 mg, 4.03 mmol), and potassium carbonate (660 mg, 4.78 mmol) in THF (5 mL) and H_2O (0.5 mL) was degassed with N_2 for 10 min. $PdCl_2(dppf)·CH_2Cl_2$ (110 mg, 0.13 mmol) was added, and the solution heated at 70 °C for 16 h. The reaction mixture was then cooled to room temperature and filtered through Celite® under vacuum. The filtrated was concentrated under reduced pressure and the residue purified *via* column chromatography (SiO_2, EtOAc/Petroleum Ether = 1:5) to yield **2** as a pale yellow oil (177 mg, 0.68 mmol, 86%).

R_f (SiO_2, EtOAc/Petroleum Ether = 1:5) 0.20. **^{1}H NMR** (400 MHz, $CDCl_3$) δ 6.60 – 6.51 (m, 2H, H^k), 6.50 (s, 1H, H^j), 6.33 (d, 2H, *J* = 17.3 Hz, H^l), 5.53 (dd, 2H, *J* = 10.6, 1.4 Hz, H^l), 5.25 (br t, 1H, *J* = 5.6 Hz, H^g), 4.10 (q, 2H, *J* = 7.1 Hz, H^b), 3.50 (q, 2H, *J* = 6.6 Hz, H^f), 2.38 (t, 2H, *J* = 7.4 Hz, H^d), 1.93 (qn, 2H, *J* = 7.1 Hz, H^e), 1.21 (t, 3H, *J* = 7.1 Hz, H^a). **^{13}C NMR** (101 MHz, $CDCl_3$) δ 173.5 (C^c), 163.8 (C^i), 162.8 (C^h), 136.0 (C^k), 121.4 (C^l), 105.7 (C^j), 60.4 (C^b), 40.8 (C^f), 31.8 (C^d), 25.2 (C^e), 14.3 (C^a). **LRMS** (ESI+) *m/z* found $[M+H]^+$ 261.6, $C_{14}H_{19}N_3O_2^+$ required 262.2.

The data are consistent with previously published data.[1]

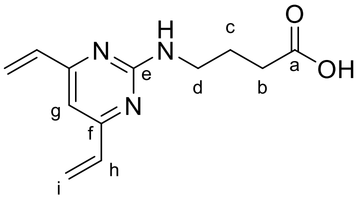

4-((4,6-divinylpyrimidin-2-yl)amino)butanoic acid (3)

To a solution of **2** (717 mg, 2.74 mmol) in THF (3 mL) and H_2O (3 mL) at 0 °C was added $LiOH·H_2O$ (180 mg, 1.56 mmol). The mixture was left to stir at room temperature for 36 h, and then concentrated under reduced pressure. The residue was taken up in saturated NH_4Cl (aq) (40 mL) and extracted with 10% iPrOH/EtOAc (4 × 120 mL), with 1 M HCl (aq) adjusting to pH 4 between extractions. The combined organic fractions were dried over

$MgSO_4$, filtered, and concentrated under reduced pressure to yield **3** as an off-white solid (376 mg, 1.69 mmol, 62%).

R_f 0.39 (SiO_2, 5% MeOH in CH_2Cl_2 + 0.5% AcOH). **^{1}H NMR** (400 MHz, CH_3OD) δ 6.70 (s, 1H, H^g), 6.61 (dd, 2H, *J* = 17.1, 10.8 Hz, H^h), 6.37 (d, 2H, *J* = 17.2 Hz, H^i), 5.57 (dd, 2H, *J* = 10.6, 1.3 Hz, H^i), 3.48 (t, 2H, *J* = 6.8 Hz, H^d), 2.38 (t, 2H, *J* = 7.3 Hz, H^b), 1.91 (m, 2H, H^c). **^{13}C NMR** (101 MHz, MeOD) δ 177.4 (C^a), 165.3 (C^f), 164.0 (C^e), 137.0 (C^h), 122.1 (C^i), 105.7 (C^g), 41.5 (C^d), 32.4 (C^b), 26.2 (C^c). **LRMS** (ESI-) *m/z* found $[M-H]^-$ 232.1, $C_{12}H_{14}N_3O_2^-$ required 232.1.

The data are consistent with previously published data.[1]

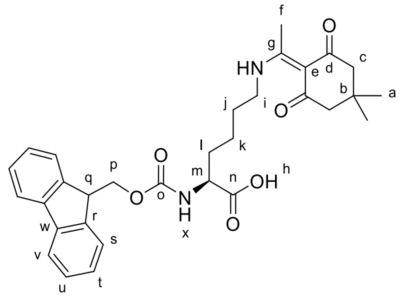

N^2-(((9*H*-fluoren-9-yl)methoxy)carbonyl)-N^6-(1-(4,4-dimethyl-2,6-dioxocyclohexylidene)ethyl)-*L*-lysine (4)

Fmoc-Lys(Boc)-OH (2.50g, 5.34 mmol) was dissolved in 4M HCl/dioxane (60 mL) and stirred at rt for 2 h. The solvent was then removed under reduced pressure. The residue was dissolved in EtOH (30 mL), and 2-acetyldimedone (1.48 g, 8.10 mmol, 1.5 equiv.) and DIPEA added, and the mixture heated at 75 °C for 17 h. After cooling to rt, then solvent was removed under reduced pressure. The residue was dissolved in CH_2Cl_2 (50 mL) and washed with 1 M HCl (2 × 50 mL). The organic layer was dried over Na_2SO_4, filtered, and concentrated. The residue was purified by flash column chromatography (SiO_2, 0 to 10% MeOH in CH_2Cl_2) to give **4** as an off-white solid (1.34 g, 2.51 mmol, 47%).

R_f 0.18 (SiO_2, 5% MeOH in CH_2Cl_2). **^{1}H NMR** (400 MHz, CDC l_3) δ 13.34 (s, 1H, H^h), 7.75 (d, 2H, *J* = 7.5 Hz, H^v), 7.60 (t, 2H, *J* = 7.5 Hz, H^s), 7.38 (t, 2H, *J* = 7.4 Hz, H^u), 7.30-7.26 (m, 2H, H^t), 5.79 (d, 1H, *J* = 8.2 Hz, H^x), 4.48-4.45 (m, 1H, H^m), 4.37 (d, 2H, *J* = 6.7 Hz, H^p), 4.20 (t, 1H, *J* = 6.8 Hz, H^q), 3.43-3.40 (m, 2H, H^i), 2.55 (s, 3H, H^f), 2.37 (s, 4H, H^c), 1.98-1.55 (m, 6H, H^j + H^k + H^l), 1.02 (s, 6H, H^a). **^{13}C NMR** (101 MHz, $CDCl_3$) δ 198.4 (C^d), 187.6 (C^n), 174.6 (C^g), 156.3 (C^o), 144.0 (C^r), 141.4 (C^w), 127.8 (C^u), 127.2 (C^v), 125.3 (C^t), 120.1 (C^s), 107.9 (C^e), 67.2 (C^p), 53.5 (C^m), 52.5 (C^c), 47.3 (C^q), 43.5 (C^i), 32.1 (C^l +C^j), 30.3 (C^b), 28.4 (C^a), 22.6 (C^k), 18.3 (C^f). **LRMS** (ESI+) *m/z* found $[M+H]^+$ 533.6, $C_{31}H_{38}N_2O_6^+$ required 533.3.

The data are consistent with previously published data.[2]

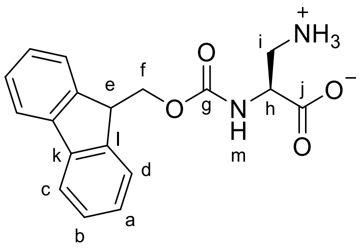

(*S*)-2-((((9*H*-fluoren-9-yl)methoxy)carbonyl)amino)-3-ammoniopropanoate (5)

To a solution of [bis(trifluoroacetoxy)iodo]benzene (5.4 g, 16.9 mmol) in DMF/H_2O (2:1, 79 mL) was added Fmoc-Asn-OH (4.00 g, 11.3 mmol). The solution was stirred for 15 min, after which pyridine (2.10 mL) was added and the mixture stirred for 14 h. The solvent was removed under reduced pressure. The oily residue was taken up in H_2O (60 mL), acidified with concentrated HCl (2 mL), and washed with Et_2O (4 × 40 mL). The aqueous phase was then adjusted to pH 6 with 5 M NaOH, and the resulting precipitate was filtered, washed with H_2O, ice-cold EtOH, Et_2O, and dried *in vacuo* to yield **5** as a beige powder (2.76 g, 8.37 mmol, 74%).

^{1}H NMR (400 MHz, DMSO-*d*$_6$) δ 7.89 (d, *J* = 7.5 Hz, 2H, H^c), 7.70 (d, *J* = 7.2 Hz, 2H, H^d), 7.40 (t, *J* = 7.5 Hz, 2H, H^b), 7.35 (t, *J* = 7.5 Hz, 2H, H^a), 6.95 (d, *J* = 6.1 Hz, 1H, H^m), 4.29-4.20 (m, 3H, H^e + H^f), 3.84-3.79 (m, 1H, H^h), 3.00 (m, 1H, H^i), 2.88 (m, 1H, H^i). **^{13}C NMR** (101 MHz, DMSO-*d*$_6$) δ 171.6 (C^j), 155.8 (C^g), 143.9 (C^l), 140.7 (C^k), 128.9 (C^b), 127.6 (C^a), 125.3 (C^c), 120.1 (C^d), 65.7 (C^f), 51.8 (C^h), 46.6 (C^e), 40.7 (C^i). **LRMS** (ESI+) *m/z* found $[M+H]^+$ 327.2, $C_{18}H_{20}N_2O_4^+$ required 327.1.

The data are consistent with previously published data.[3]

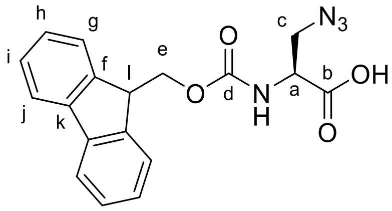

(*S*)-2-((((9*H*-fluoren-9-yl)methoxy)carbonyl)amino)-3-azidopropanoic acid (6)

To a solution of **5** (2.07 g, 6.34 mmol) in H_2O/CH_2Cl_2/MeOH (1:2:3, 180 mL) was added $CuSO_4{\cdot}5H_2O$ (20 mg, 0.081 mmol), and **7** (4.6 g, 17.0 mmol). The mixture was adjusted to pH 9 with saturated K_2CO_3 and stirred for 22 h. The solution was then diluted with CH_2Cl_2 (120 mL) and the aqueous phase isolated. The organic phase was extracted with saturated $NaHCO_3$ (2 × 200 mL). The aqueous phases were combined, washed with Et_2O (2 × 200 mL), adjusted to pH 2 with concentrated HCl, and then extracted with Et_2O (3 × 240 mL). The combined organic extracts were dried over $MgSO_4$, filtered, and concentrated under reduced pressure. The residue was taken up in EtOAc and

concentrated under a stream of N_2 to yield **6** as an amorphous beige solid (1.77 g, 5.03 mmol, 79%).

^{1}H NMR (400 MHz, DMSO-d_6) δ 7.94-7.88 (m, 2H, H^j), 7.73 (d, *J* = 7.5 Hz, 2H, H^g), 7.44-7.40 (app t, *J* = 7.3 Hz, 2H, H^i), 7.35-7.32 (app t, *J* = 7.4 Hz, 2H, H^h), 4.33-4.30 (m, 2H, H^e), 4.21-4.26 (m, 2H, H^l + H^a), 3.64-3.60 (m, 2H, H^c). **^{13}C NMR** (101 MHz, DMSO-*d*6) δ 171.1 (C^b), 156.1 (C^d), 143.8 (C^f), 140.7 (C^k), 127.7 (C^i), 127.1 (C^h), 125.3 (C^g), 120.1 (C^j), 65.9 (C^e), 53.8 (C^a), 50.9 (C^c), 46.6 (C^l). **LRMS** (ESI+) *m/z* found $[M+H]^+$ 353.2, $C_{18}H_{17}N_4O_4^+$ required 353.1.

The data are consistent with previously published data.[4]

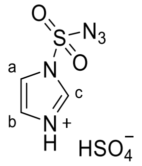

1-(Azidosulfonyl)-1*H*-imidazol-3-ium hydrogen sulfate (7)

Sulfuryl chloride (16.1 mL, 199 mmol) was added dropwise (1 h) to an ice-cold suspension of NaN_3 (13.0 g, 200 mmol) in MeCN (200 mL) and stirred at 0 °C for 16 h. Imidazole (25.9 g, 380 mmol) was then added to the suspension, and the solution was allowed to warm to room temperature and stir for a further 5 h. The solution was then diluted with EtOAc (400 mL), washed with H_2O (2 × 400 mL) and $NaHCO_3$ (2 × 200 mL), dried over $MgSO_4$, filtered and concentrated under reduced pressure to approximately 200 mL. The solution was then cooled to 0 °C, and acidified dropwise over 30 min with a solution of 10% H_2SO_4/EtOAc (110 mL). The solution was warmed to room temperature and stirred overnight. The white precipitate formed was collected under vacuum filtration, washed with EtOAc and dried to yield **7** as a beige powder (23.5 g, 86.6 mmol, 43%).

^{1}H NMR (400 MHz, DMSO-d_6) δ 8.63 (s, 1H, H^c), 7.98 (app t, *J* = 1.56, 1H, H^a) 7.34 (app q, *J* = 0.88 Hz, 1H, H^b). **^{13}C NMR** (101 MHz, DMSO-d_6) δ 137.8 (C^c), 130.6 (C^a), 118.9. (C^b).

The data are consistent with previously published data.[5]

1.3 Peptide Synthesis

Peptides were either synthesised by manual or automated solid-phase peptide synthesis (SPPS), or a combination thereof. For example, peptides whose synthesis necessitated the removal of an Alloc or Dde group were synthesised *via* automated SPPS up until the deprotection step, whereupon the resin was removed from the automated synthesiser and the synthesis continued manually from thereon (so as to avoid the use of harsh reagents on the automated synthesiser and/or reduce the chance of instrument contamination).

Manual SPPS

Manual SPPS was performed on MHBA Rink Amide resin (0.308 mmol/g, 100-200 mesh). Fmoc-protected amino acids were employed, and triphenylmethyl used as a side chain protecting group for Gln.

Couplings were performed by adding HATU (2.9 equiv.) to a solution of the Fmoc-protected amino acid (3.9 equiv.) in DMF. This was added to the resin, and after stirring briefly, DIPEA (6 equiv.) was added, and the mixture agitated for 3-10 minutes. Fmoc deprotection was performed with 20% piperidine in DMF at room temperature with agitation. N-terminal acetyl capping was performed with Ac_2O (50 equiv.) and DIPEA (50 equiv.) in DMF, shaking for 1 hour.

The completion of amide couplings and Fmoc deprotection was determined by Kaiser test. Briefly, 3 drops each of phenol, ninhydrin, and potassium cyanide were added to a small vial containing a small amount of resin swelled in CH_2Cl_2. The mixture was heated gently, with blue colouration of the resin indicating the presence of a free amine. A coupling was repeated where the test indicated the presence of free amine.

Side chain deprotection and cleavage from the resin was achieved with TFA:TIPS:H_2O (95:5:5) for 1 hour at 42 °C, after which the mixture was filtered, and the filtrate concentrated under a stream of N_2, and purified *via* preparative HPLC.

Automated SPPS

Automated SPPS was performed using a CEM Liberty Blue Automated Microwave Peptide Synthesiser using MHBA Rink Amide resin (0.308 mmol/g, 100-200 mesh). All peptide couplings were performed with Fmoc-protected amino acids (5 equiv.), Oxyma pure (10 equiv.) and DIC (5 equiv.) in DMF. All amino acids were coupled with 25 W power at 75 °C over 15 min.

Fmoc deprotection was performed with a solution of 20% piperidine in DMF, with 45 W power at 75 °C over 3 min. Cleavage and HPLC purification of peptides were carried out as previously described for manual SPPS.

Table S1. Peptide sequence, mass spectrometry and analytical HPLC data for peptides. All peptides are C-terminal amides, and are uncapped at the N-terminus and sidechains unless otherwise specified. Retention time determined by analytical HPLC (5-95% MeCN in H_2O over 15 min). Ac = acetyl; Ahx = 6-aminohexanoic acid; Oeg = 8-amino-3,6-dioxaoctanoic acid; Mca = 2-(7-methoxy-2-oxo-2H-chromen-4-yl)acetyl; Dnp = 2,4-dinitrophenyl; Pra = propargylglycine; Aza = azaalanine **6**.

Peptide	Sequence	m/z found	m/z calc.	Species	Purity / %	Rt / min
**P1**	Ahx_2GPLGIAGQK(Ahx_2)	646.8	646.3	M+2H	100	6.06
**P2**	Ahx_2Oeg_4K(Ahx_2)	1179.1	1178.8	M+H	84	5.67
**P3**	Oeg_2PraGPLGIAGQPraK(Oeg_2)	806.4	805.4	M+2H	100	6.18
**P4**	$Oeg_2PraOeg_4$PraK(Oeg_2)	1497.5	1497.8	M+H	90	5.34
**P5**	Oeg_2AzaGPLGIAGQPraK(Oeg_2)	814.4	813.9	M+2H	100	5.64
**P6**	$Oeg_2AzaOeg_4$PraK(Oeg_2)	757.5	757.4	M+2H	100	5.55

Revised synthesis of **P3** and **P4**

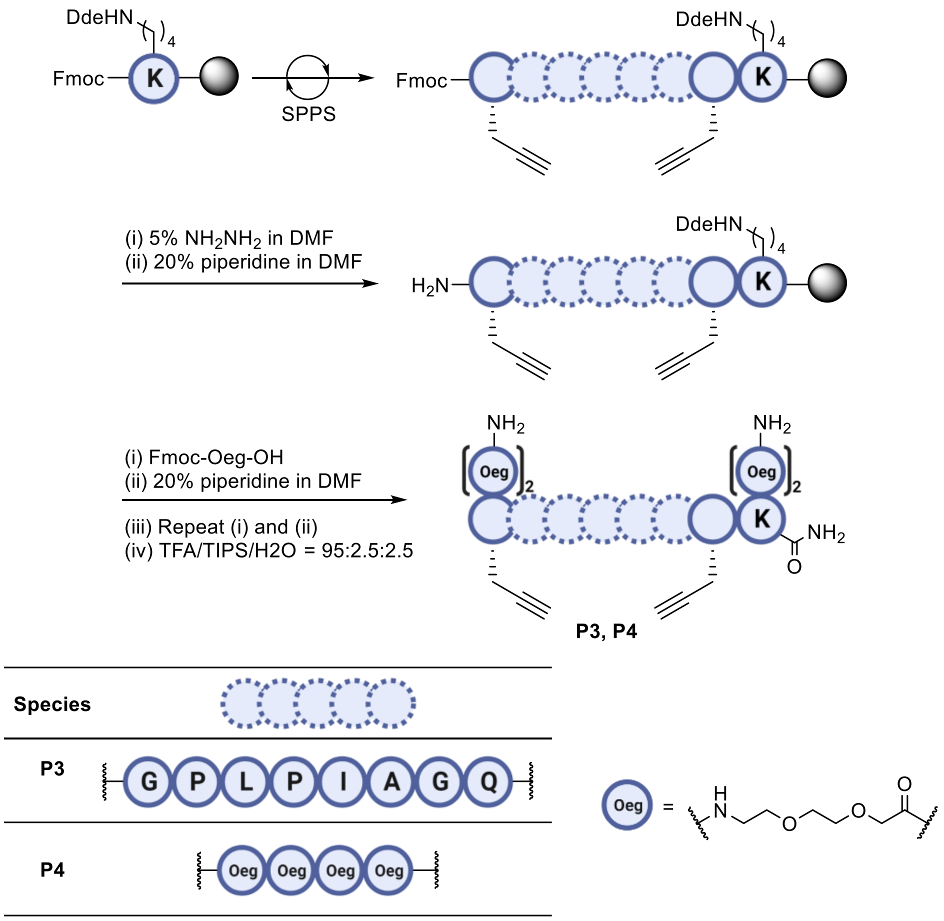

Figure S1: Revised SPPS protocol for the syntheses of **P3** and **P4** using Dde protected lysine **4**.

1.4 BisDVP Linker Synthesis

General Method A: Synthesis of BisDVP Linkers *via* DVP Amide Coupling

A solution of DVP **3** (6 equiv.), HATU (5.5 equiv.), and DIPEA (10 equiv.) in DMF was stirred at rt for 5 min. To this solution, was added a solution of peptide **P1, P2, P3**, **P4**, **P5**, or **P6** (2 × TFA salt; 1 equiv.) in DMF. The resulting solution was stirred at rt for 1 hr, and then concentrated under a gentle stream of N_2. Purification by automated reverse phase flash column chromatography (RediSep Gold C18 High Performance, 5.5 g; 10-100% Solvent B in Solvent A; Solvent A: 0.1 M NH_4OH, Solvent B: MeCN) followed by lyophilisation yielded linker **B1, B2, B3**, **B4**, **B5**, or **B6** as white powders. Peptide linkers were characterised by LRMS and HPLC.

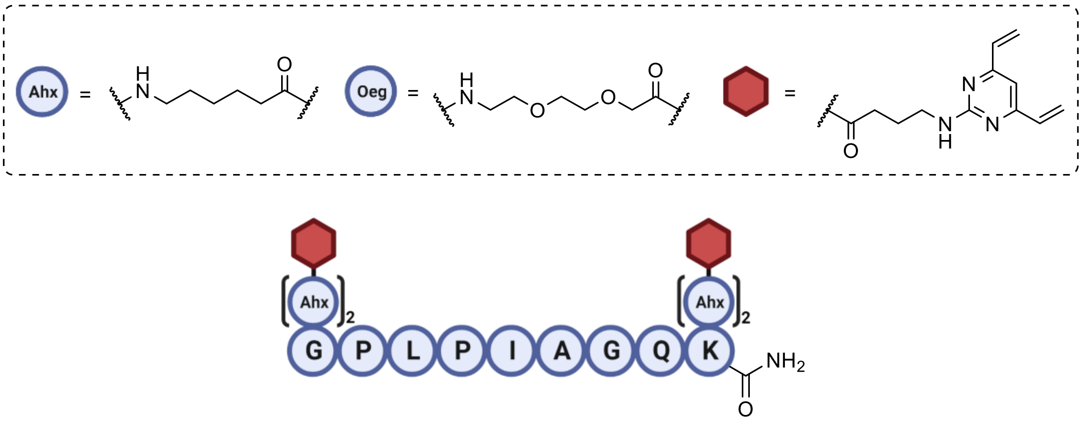

BisDVP **B1** was prepared from **P1**·2TFA (9.69 mg, 6.38 µmol) according to General Method A, yielding a white powder (2.00 mg, 1.16 µmol, 18%).

LRMS (ESI) m/z found $[M+2H]^{2+}$ 861.7; $C_{85}H_{136}N_{22}O_{16}$ required 861.5. **HPLC** (5-95% MeCN/H_2O over 15 min) retention time 7.97 min; (5-55% MeCN/H_2O over 15 min) retention time 11.45 min.

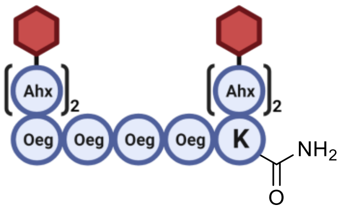

BisDVP **B2** was prepared from **P2**·2TFA (7.40 mg, 5.33 μmol) according to General Method A, yielding a white powder (2.44 mg, 1.52 μmol, 28%).

LRMS (ESI) m/z found $[M+2H]^{2+}$ 805.4; $C_{78}H_{129}N_{17}O_{19}$ required 805.0. **HPLC** (5-95% $MeCN/H_2O$ over 15 min) retention time 7.68 min; (5-50% $MeCN/H_2O$ over 15 min) retention time 11.57 min.

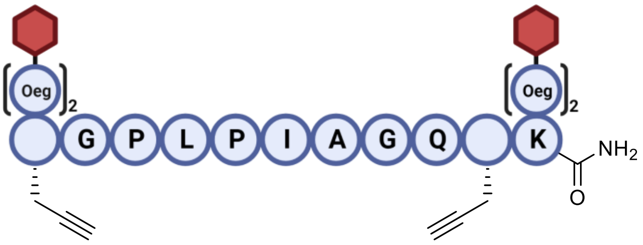

Bis DVP **B3** was prepared from **P3**·2TFA (10.0 mg, 5.44 μmol) according to General Method A, yielding a white powder (9.89 mg, 4.85 μmol, 89%).

LRMS (ESI) m/z found $[M+2H]^{2+}$ 1021.2; $C_{95}H_{146}N_{24}O_{26}$ required 1021.0. **HPLC** (5-95% $MeCN/H_2O$ over 15 min) retention time 7.86 min; (10-60% $MeCN/H_2O$ over 15 min) retention time 9.78 min.

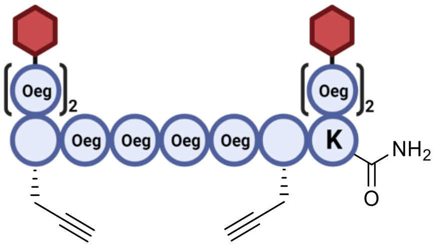

Bis DVP **B4** was prepared from **P4**·2TFA (10.0 mg, 5.80 μmol) according to General Method A, yielding a white powder (4.63 mg, 2.40 μmol, 41%).

LRMS (ESI) m/z found $[M+2H]^{2+}$ 964.5; $C_{88}H_{139}N_{19}O_{29}$ required 964.5. **HPLC** (5-95% $MeCN/H_2O$ over 15 min) retention time 7.28 min.

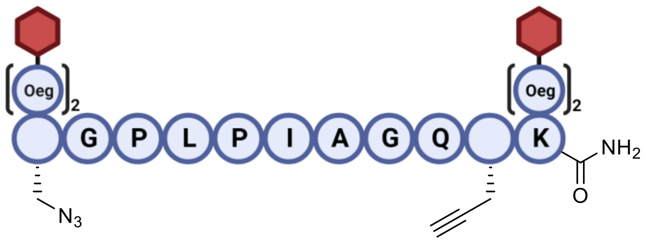

Bis DVP **B5** was prepared from **P5**·2TFA (10.0 mg, 5.39 μmol) according to General Method A, yielding a white powder (9.20 mg, 4.47 μmol, 83%).

LRMS (ESI) m/z found $[M+H]^+$ 2058.1; $C_{93}H_{145}N_{27}O_{26}$ required 2058.1. **HPLC** (5-95% MeCN/H_2O over 15 min) retention time 8.37 min; (10-60% MeCN/H_2O over 15 min) retention time 10.91 min.

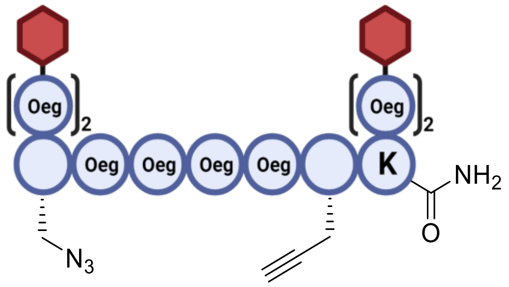

Bis DVP **B6** was prepared from **P6**·2TFA (10.0 mg, 5.74 μmol) according to General Method A, yielding a white powder (6.57 mg, 3.38 μmol, 59%).

LRMS (ESI) m/z found $[M+3H]^{3+}$ 648.9; $C_{86}H_{138}N_{22}O_{29}$ required 649.0. **HPLC** (5-95% MeCN/H_2O over 15 min) retention time 8.20 min; (10-60% MeCN/H_2O over 15 min) retention time 10.58 min.

1.5 Protein Chemistry

SDS-PAGE

Non-reducing Tris-Glycine SDS-PAGE with 12% acrylamide with 4% stacking gel was performed as standard. Broad range molecular weight marker (10-200 kDa, New England BioLabs) was run in all gels. Samples (10 μL at approximately 0.4 mg/mL) were prepared with reducing or non-reducing loading dye (10 μL, reducing dye contains β-mercaptoethanol) and heated to 90 °C for 5 min prior to loading. Gels were run at constant voltage (160 V or 200 V) for 80 min in 1 × Laemmli running buffer (LRB). All gels were stained with Coomassie dye and imaged on a Syngene gel imaging system. Gels containing fluorescently labelled samples were imaged for in-gel fluorescence prior to Coomassie staining.

UV-vis spectroscopy

UV-vis spectroscopy was used to determine protein concentrations and fluorophore to antibody ratios (FAR) using a NanoDrop One spectrophotometer. Sample buffer was used as a blank for baseline correction. The following extinction coefficients were used: ε_{280} = 215380 M^{-1} cm^{-1} for trastuzumab IgG; ε_{280} = 68590 M^{-1} cm^{-1} for trastuzumab Fab; ε_{280} = 72900 M^{-1} cm^{-1} for cetuximab Fab; ε_{280} = 79300 M^{-1} cm^{-1} for durvalumab Fab (extinction coefficients for IgG and Fab were determined theoretically from amino acid sequence data[6]); ε_{280} = 2540 M^{-1} cm^{-1} for divinylpyrimidine residue;[7] ε_{495} = 71000 M^{-1} cm^{-1} for Alexa Fluor® 488;[8] ε_{555} = 65000 M^{-1} cm^{-1} for TAMRA.[9] Correction factors for the absorption at 280 nm of Alexa Fluor® 488 and TAMRA were 0.11 and 0.30 respectively, obtained from the manufacturer. Extinction coefficients for synthesised protein dimers were determined from the sum of the extinction coefficients of the constituent parts. Fluorophore antibody conjugates were washed *via* ultrafiltration, with periodic analysis of the FAR *via* UV-vis spectroscopy, until no change in FAR was detected.

Protein LCMS

Protein LC–MS was performed on a Xevo G2-S TOF mass spectrometer coupled to an Acquity UPLC system using an Acquity UPLC BEH300 C4 column (1.7 μm, 2.1 × 50 mm). H_2O with 0.1% formic acid (solvent A) and 95% MeCN and 5% water with 0.1% formic acid (solvent B), were used as the mobile phase at a flow rate of 0.2 mL/min. The gradient was programmed as follows: 95% A for 0.93 min, then a gradient to 100% B over 4.28 min, then 100% B for 1.04 minutes, then a gradient to 95% A over 1.04 min. The electrospray source was operated with a capillary voltage of 2.0 kV and a cone voltage of 150 V. Nitrogen was used as the desolvation gas at a total flow of 850 L/h. Total mass spectra were reconstructed from the ion series using the MaxEnt 1 algorithm preinstalled on MassLynx 4.2 software according to the manufacturer's instructions. In the main body of this thesis, some mass spectra are presented with the aid of image editing software, wherein peak annotations have been enlarged or removed (where overlapping), solely for

the purposes of improving readability. All deconvoluted mass spectra and ion series as generated in MassLynx are presented without modification in Appendix C. Trastuzumab samples were deglycosylated with PNGase F (New England Biolabs) prior to LCMS analysis, unless otherwise specified. Only the region of the total ion chromatograph (TIC) between 3.25-3.75 min was analysed. Peaks outside of this range did not contain proteinogenic signals and were excluded. Analysis was conducted in the same way for all protein LCMS traces.

Hydrophobic Interaction Chromatography (HIC)

Hydrophobic interaction chromatography (HIC) was performed on an Agilent 1260 Infinity using a Tosoh Bioscience TSKgel Butyl-NPR column (4.6 mm ID × 3.5 cm L) at a flow rate of 0.6 mL/min. Proteins were eluted using a linear gradient (0% B for 3 min then gradate to 100% B over 17 min) of Solvent B in Solvent A (Solvent A: 1.5 M ammonium sulfate, 25 mM NaPi, pH 7; Solvent B: 25% isopropyl alcohol in 25 mM NaPi, pH 7).

Size-exclusion chromatography (SEC)

Analytical size-exclusion chromatography (SEC) was performed on an Agilent 1260 Infinity using a Tosoh Bioscience TSKgel G3000SWXL column (7.8 mm × ID 30 cm L). Proteins were eluted with NaPi pH 7 (50 mM NaPi, 100 mM NaCl, 0.02% NaN_3) at a flow rate of 0.6 mL/min.

Preparative SEC was performed on an AKTA pure chromatography system using a Superdex™ 200 Increase 10/300 GL column. Samples were injected at a concentration of approximately 1 mg/mL and eluted with either TBS pH 8 (25 mM Tris HCl, 200 mM NaCl, 0.5 mM EDTA) or PBS at a flow rate of 0.5 mL/min.

General Method B: Fab Preparation

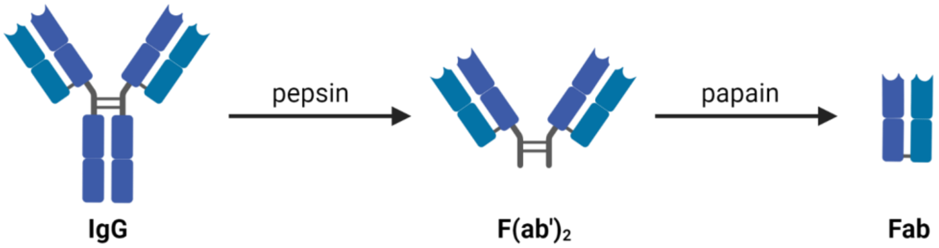

Immobilised pepsin (Thermo Scientific, Cat. No. 20343; 150 μL) was isolated in an enzyme column and washed with Digest Buffer 1 (20 mM NaOAc, pH 3.1; 4 × 200 μL). Antibody (approx. 8 mg) was dissolved in Digest Buffer 1 (500 μL), added to the immobilized pepsin, and shaken vigorously overnight at 37 °C. The resin was separated from the filtrate, and washed with Digest Buffer 2 (4 × 200 μL; 50 mM NaPi, 150 mM NaCl, 1 mM EDTA, pH 6.8). The filtrates were combined and subjected to repeated membrane

filtration with Digest Buffer 2. Immobilised papain (Thermo Scientific, Cat. No. 20341; 500 μL) was isolated in an enzyme column, washed with Digest Buffer 2 (2 × 500 μL), and activated with DTT (500 μL, 10 mM) at 37 °C for 1 h. The resin was separated from the DTT solution, and washed with Digest Buffer 2 (4 × 500 μL). The antibody fragment solution was added to the resin, and incubated at 37 °C overnight. The resin was then isolated, washed with TBS (25 mM Tris, 25 mM NaCl, 0.5 mM EDTA, pH 8.0; 4 × 500 μL), and the filtrates combined and subjected to repeated membrane filtration with TBS, to yield a solution of the antibody Fab in TBS.

General Method C: Fab Reduction

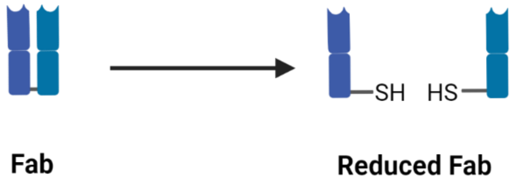

A solution of antibody Fab in TBS (25 mM Tris HCl, 25 mM NaCl, 0.5 mM EDTA, pH 8) was diluted with TBS and TCEP (5 equiv.) added. The mixture was vortexed, incubated at 37 °C for 1 h, and passed through a ZebaSpin desalting column (7000 MWCO, Thermo Scientific). The concentration was confirmed by UV-vis spectroscopy prior to further use.

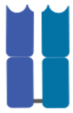

Trastuzumab Fab

Trastuzumab Fab was prepared from Herceptin® according to General Method B.

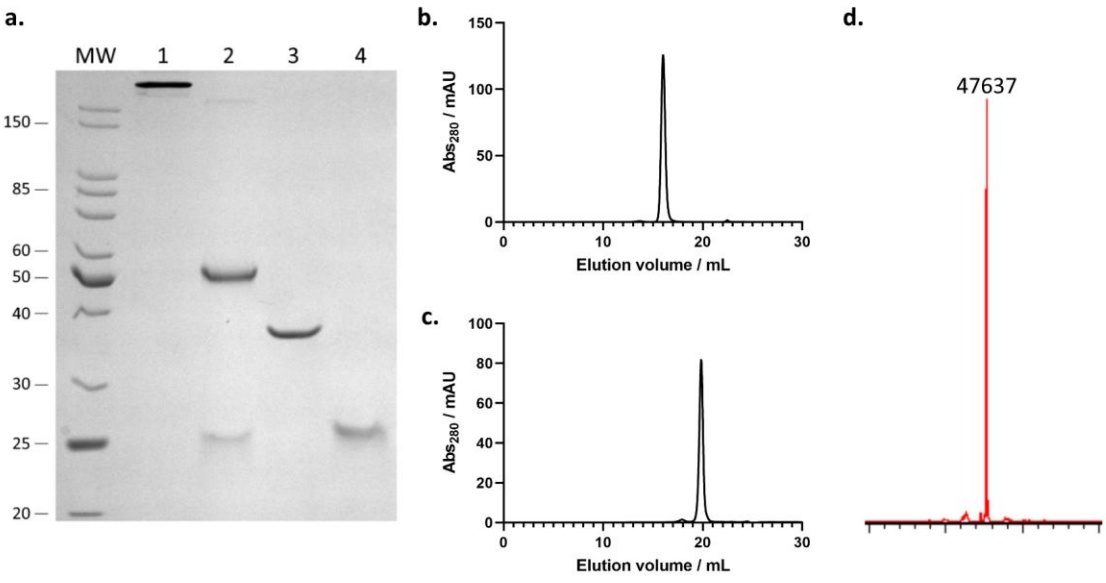

(a) SDS-PAGE analysis. Lanes MW = molecular weight marker; 1 = Herceptin®; 2 = Herceptin® [reducing conditions]; 3 = trastuzumab Fab; 4 = trastuzumab Fab [reducing conditions]; (b) SEC analysis of Herceptin; (c) SEC analysis of trastuzumab Fab; and (d) deconvoluted MS analysis of trastuzumab Fab (expected 47638 Da, observed 47637 Da).

Cetuximab Fab

Erbitux® (Merck, 2 mg/mL) was buffer exchanged into 20 mM NaOAc, pH 3.1 *via* repeated diafiltration. From this solution, cetuximab Fab was prepared according to General Method B, and purified by size-exclusion chromatography.

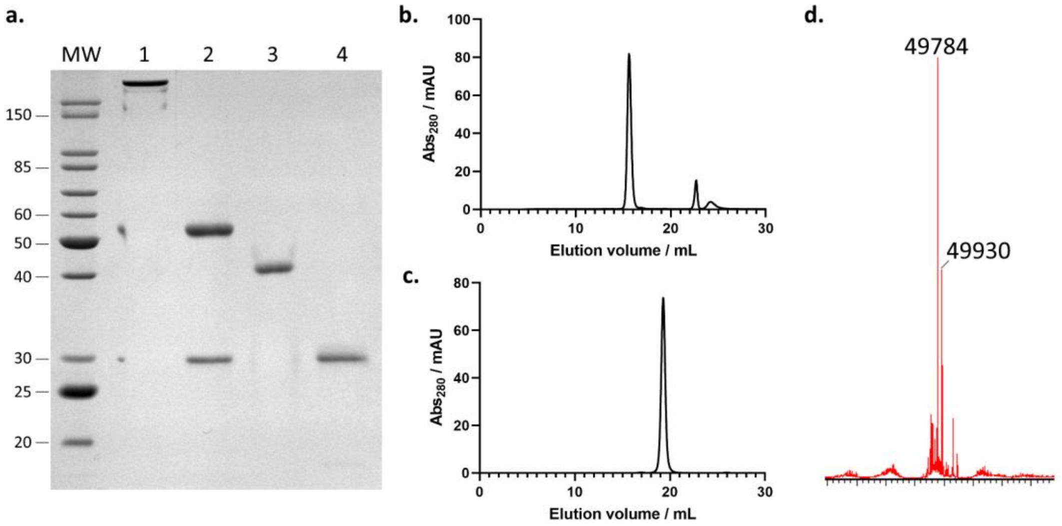

(a) SDS-PAGE analysis. Lanes: MW = molecular weight marker; 1 = Erbitux®; 2 = Erbitux® [reducing conditions]; 3 = cetuximab Fab; 4 = cetuximab Fab [reducing conditions]; (b) SEC analysis of Erbitux®; (c) SEC analysis of cetuximab Fab; and (d) deconvoluted MS analysis of cetuximab Fab (observed 49784 Da and 49930 Da (glycosylation)).

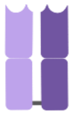

Durvalumab Fab

Imfinzi® (AstraZeneca, 50 mg/mL) was buffer exchanged into 20 mM NaOAc, pH 3.1 *via* repeated diafiltration. From this solution, durvalumab Fab was prepared according to General Method B, and purified by size-exclusion chromatography.

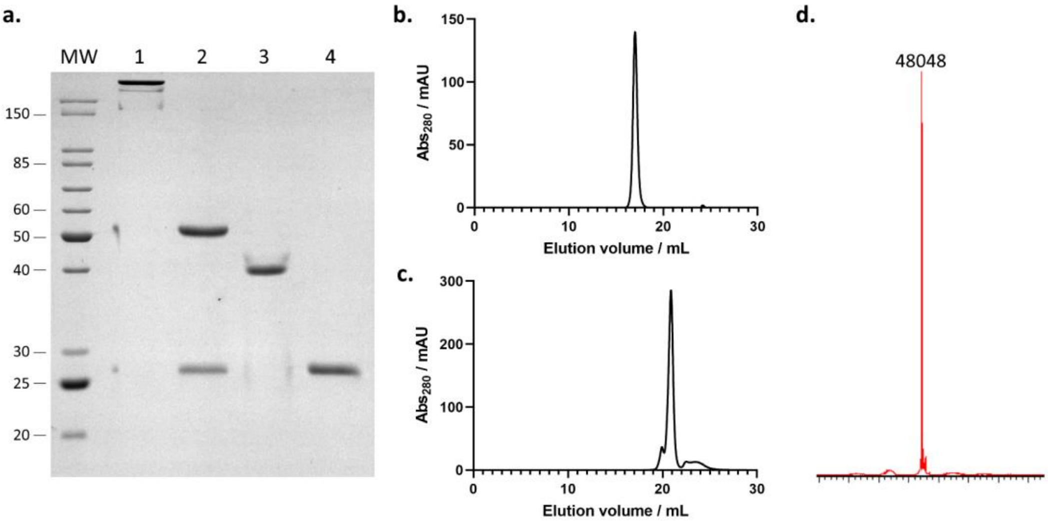

(a) SDS-PAGE analysis. Lanes: MW = molecular weight marker; 1 = Imfinzi®; 2 = Imfinzi® [reducing conditions]; 3 = durvalumab Fab; 4 = durvalumab Fab [reducing conditions]; (b) SEC analysis of Imfinzi®; (c) SEC analysis of durvalumab Fab; and (d) deconvoluted MS analysis of durvalumab Fab (observed 48048 Da, expected 48047 Da).

Preparation of unfunctionalised α-HER2×α-HER2 Fab Dimers

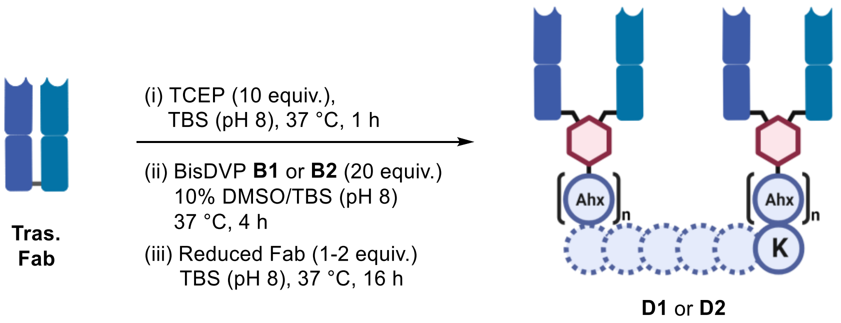

Conjugate	n	Yield (%)	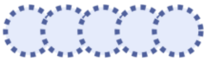
**D1** (from **B1**)	2	15	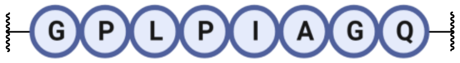
**D2** (from **B2**)	2	17	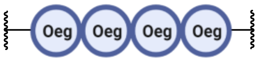

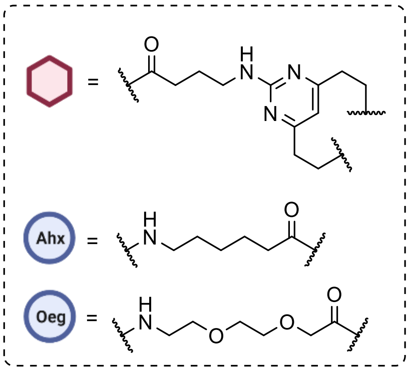

A solution of trastuzumab Fab in TBS (25 mM Tris HCl, 25 mM NaCl, 0.5 mM EDTA, pH 8) was reduced according to General Method C, diluted with TBS to approximately 2.5 mg/mL, BisDVP **B1** or **B2** (20 equiv., 10 mM in DMSO) added, and the DMSO composition adjusted to 10%. The mixture was vortexed briefly, then incubated at 37 °C for 4 h. Excess reagents were removed by passing the solution through a ZebaSpin desalting column (7000 MWCO, ThermoScientific) and repeated diafiltration with TBS. A second addition of reduced trastuzumab Fab (1-2 equiv.) was added, the solution vortexed, and incubated for 37 °C for 16 h. The dimer was isolated in 15-17% yield *via* size-exclusion chromatography.

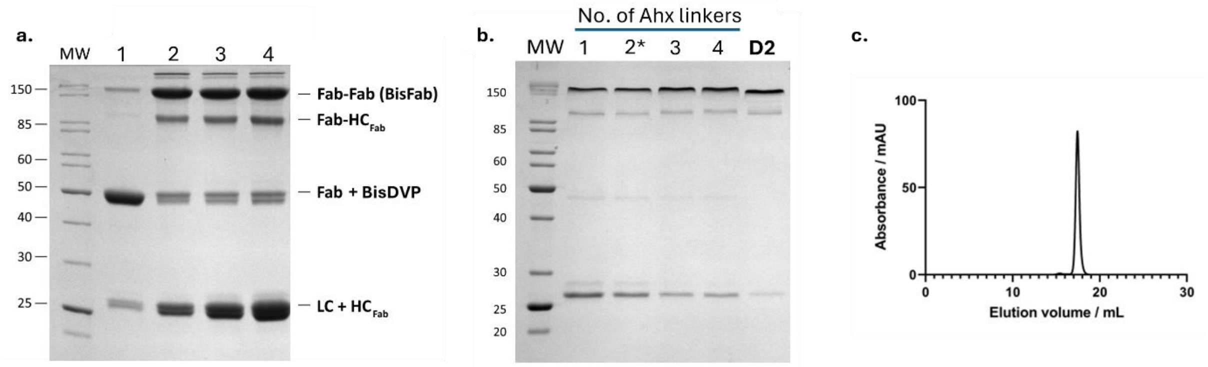

Figure S2: Extent of BisFab dimerisation. a) SDS-PAGE analysis of crude reaction mixture from screen of equivalents for the second addition of Fab in the two-step BisDVP Fab dimerisation method. Lanes: MW = molecular weight marker; 1 = initial rebridging with BisDVP **B1** (10 equiv.); 2 = 1.0 equivalents of additional reduced Fab; 3 = 1.5 equivalents of additional reduced Fab; 2.0 equivalents of additional reduced Fab. LC = light chain;

HC_{Fab} = Fab heavy chain. b) SDS-PAGE analysis of purified BisFabs. Lanes: MW = molecular weight marker; number represent number of Ahx linkers employed in the cleavable constructs, **D2** (non-cleavable); * = This is **D1**. The number of Ahx linkers made little conjugation difference and so **D1** (where Ahx = 2) was taken forward and the other lengths are not described in this work. SDS-PAGE conditions: 12% SDS, 200 V, 65 min. c) SEC analysis of **D2** following purification, showing single elution band at 17-18 mL.

Preparation of Functionalised α-HER2×α-HER2 Fab Dimers

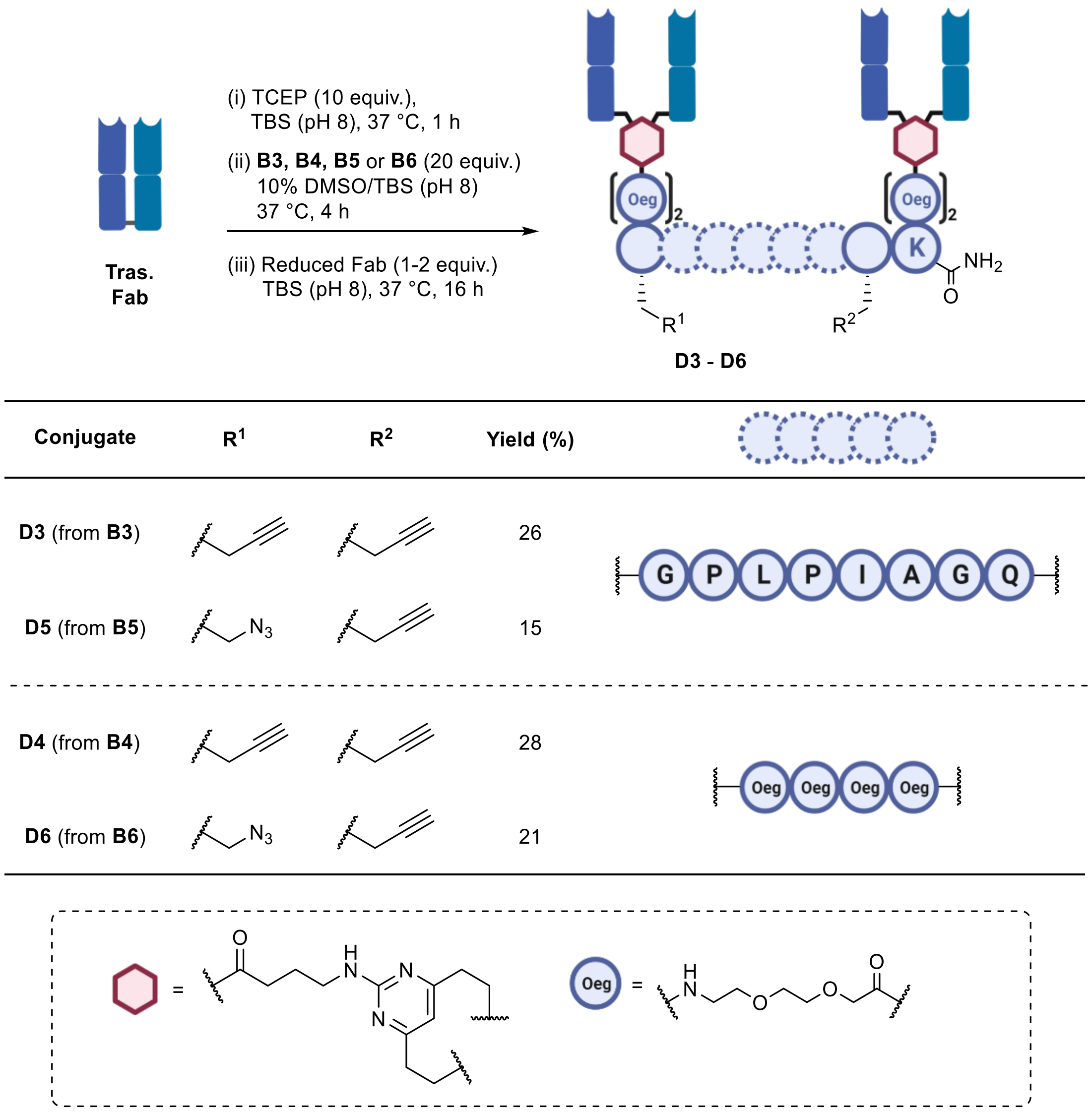

A solution of trastuzumab Fab in TBS (25 mM Tris HCl, 25 mM NaCl, 0.5 mM EDTA, pH 8) was reduced according to General Method C, diluted with TBS to approximately 2.5 mg/mL, linker **B3-B6** (20 equiv., 10 mM in DMSO) added, and the DMSO content adjusted to 10% (v/v) through addition of DMSO. The mixture was vortexed briefly, then incubated at 37 °C for 4 h. Excess reagents were removed by passing the solution through a ZebaSpin desalting column (7000 MWCO, ThermoScientific) and repeated diafiltration with TBS using an Amicon-Ultra centrifugal filter (10000 MWCO, Merck Millipore).. A

second addition of reduced trastuzumab Fab (1-2 equiv.) was added, the solution vortexed, and incubated for 37 °C for 16 h. The dimer (**D3**, **D4**, **D5**, or **D6**) was isolated *via* size-exclusion chromatography.

Preparation of α-HER2×α-HER2 AFCs

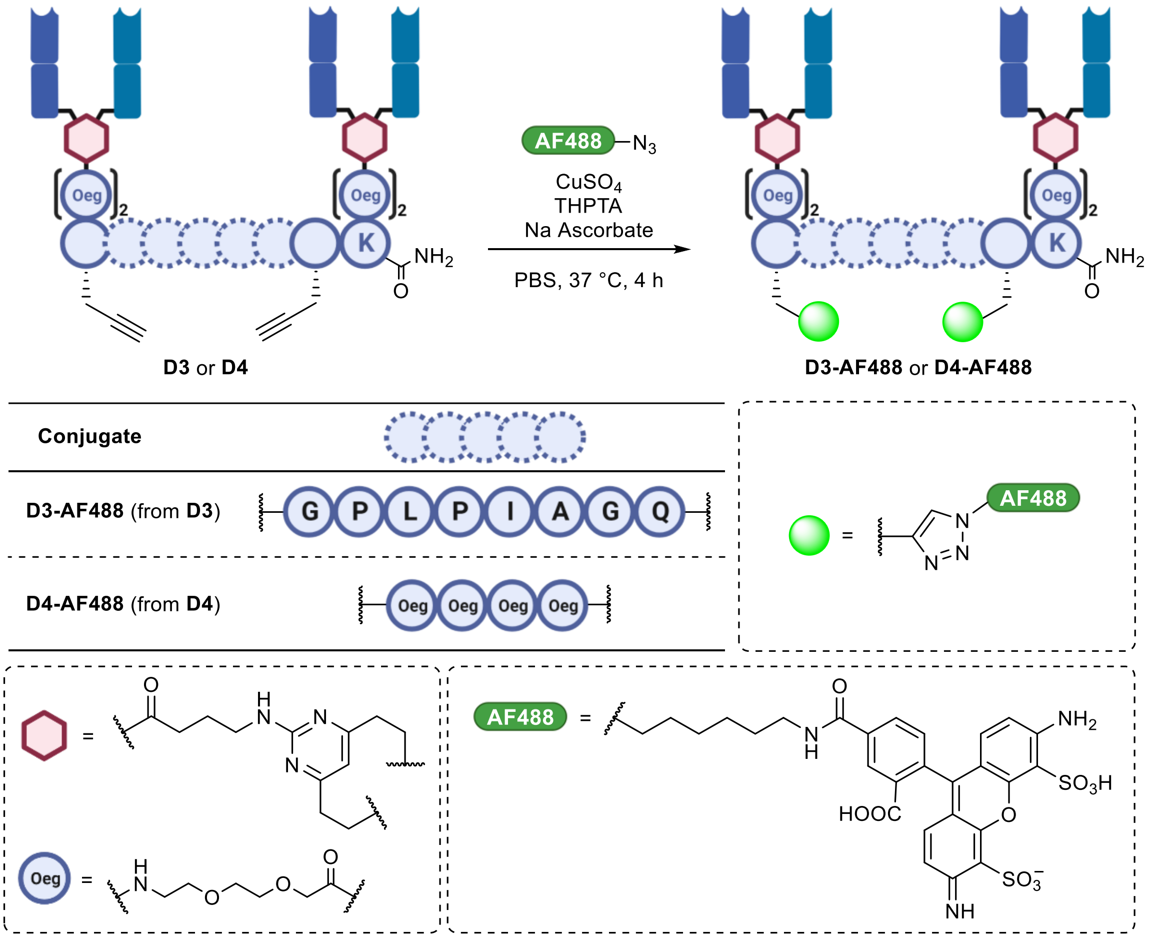

To a solution of conjugate **D3** or **D4** in PBS was added THPTA (400 equiv.), $CuSO_4 \cdot 5H_2O$ (100 equiv.), sodium ascorbate (300 equiv.) and Alexa Fluor® 488 azide (20 mM in DMSO, 50 equiv.). The DMSO content was adjusted to 10% through addition of DMSO, then the mixture was vortexed and incubated at 37 °C for 6 h. The reaction was quenched with the addition of EDTA (30 min), and the excess reagents were removed by filtration through two successive ZebaSpin Desalting Columns (40000 MWCO, ThermoFisherScientific), followed by repeated diafiltration into PBS using an Amicon-Ultra centrifugal filter (10000 MWCO, Merck Millipore).

Preparation of α-HER2×α-HER2 ADCs

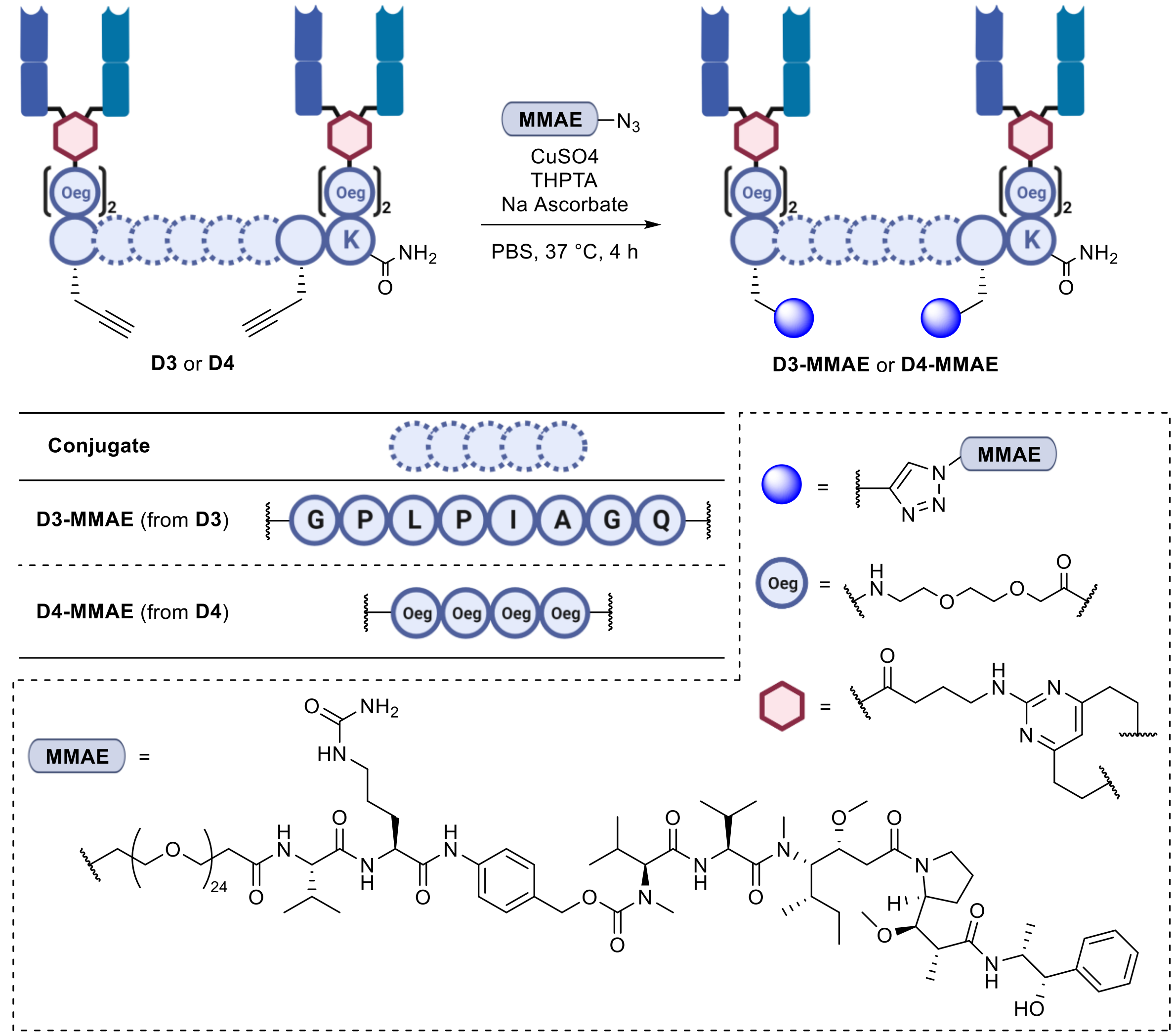

To a solution of conjugate **D3** or **D4** in PBS was added THPTA (400 equiv.), $CuSO_4{\cdot}5H_2O$ (100 equiv.), sodium ascorbate (300 equiv.) and N_3-PEG_{24}-Val-Cit-PABC-MMAE (20 mM in DMSO, 50 equiv.). The DMSO content was adjusted to 10% through addition of DMSO, then the mixture was vortexed and incubated at 37 °C for 6 h. The reaction was quenched with the addition of EDTA (30 min), and the excess reagents were removed by filtration through two successive ZebaSpin Desalting Columns (40000 MWCO, ThermoFisherScientific), followed by repeated diafiltration into PBS using an Amicon-Ultra centrifugal filter (10000 MWCO, Merck Millipore).

Preparation of α-HER2×α-HER2 TAMRA Dimers

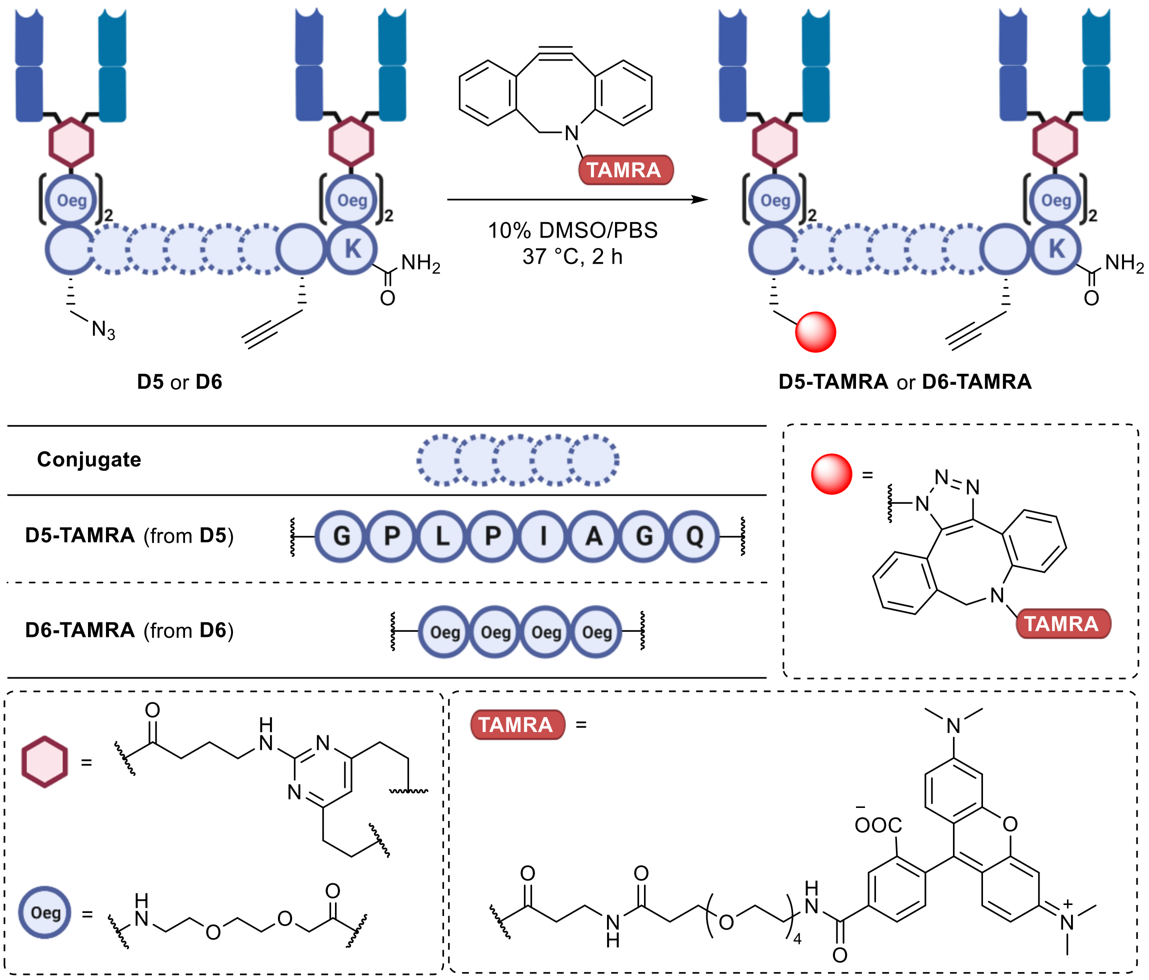

To a solution of conjugate **D5** or **D6** in PBS was added DBCO-TAMRA (20 mM in DMSO, 10 equiv.). The DMSO content was adjusted to 10% through addition of DMSO, then the mixture was vortexed and incubated at 37 °C for 1.5 h. The excess reagents were removed by filtration through two successive ZebaSpin Desalting Columns (40000 MWCO, ThermoFisherScientific), followed by repeated diafiltration into PBS using an Amicon-Ultra centrifugal filter (10000 MWCO, Merck Millipore).

Preparation of α-HER2×α-HER2 FRET Dimers

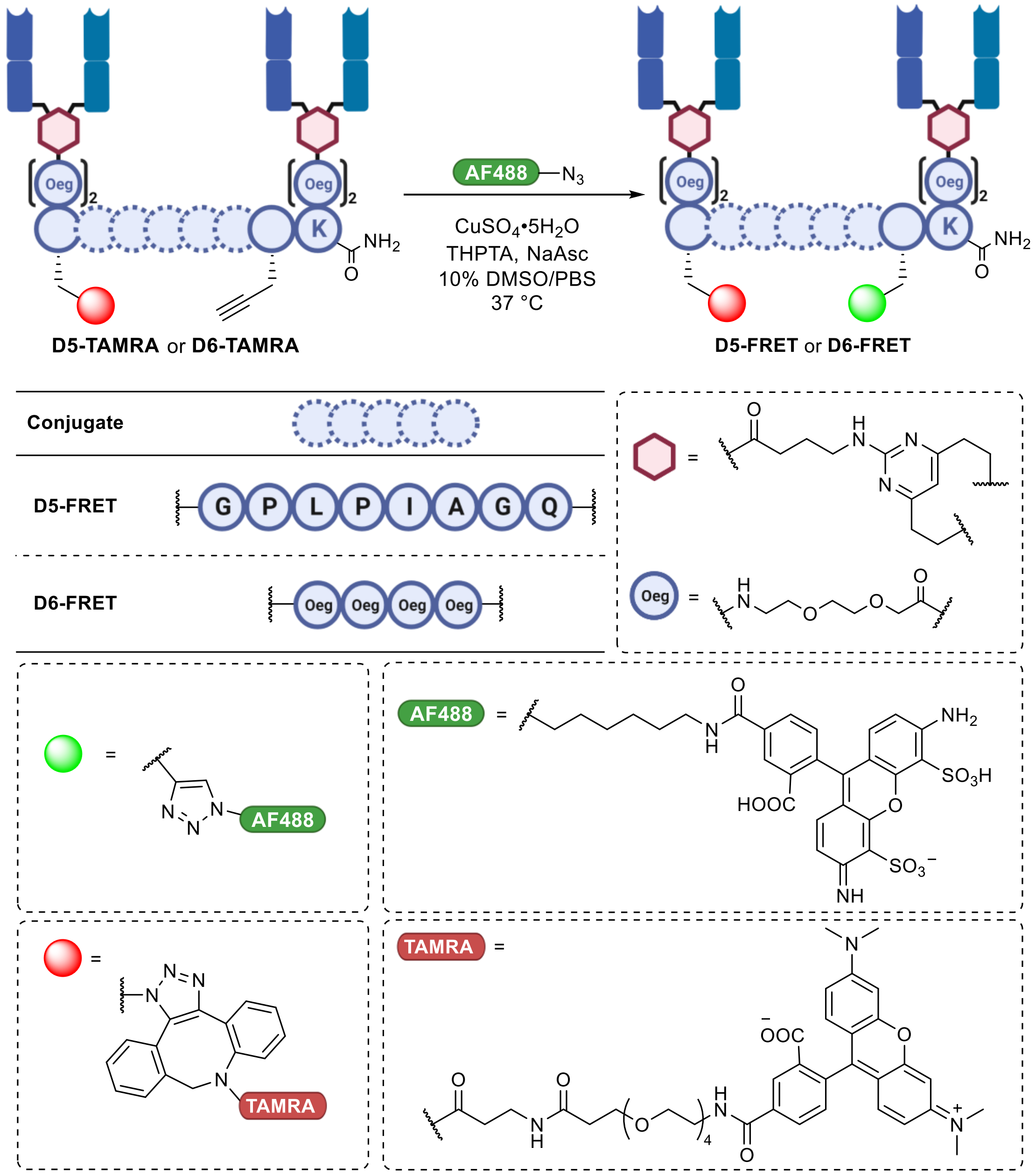

To a solution of conjugate **D5-TAMRA** or **D6-TAMRA** in PBS was added THPTA (200 equiv.), $CuSO_4 \cdot 5H_2O$ (50 equiv.), sodium ascorbate (300 equiv.) and Alexa Fluor® 488 azide (5 mM in DMSO, 50 equiv.). The DMSO content was adjusted to 10% (v/v) through addition of DMSO, then the mixture was vortexed and incubated at 37 °C for 4 h. The reaction was quenched with the addition of EDTA (30 min), and the excess reagents were removed by filtration through two successive ZebaSpin Desalting Columns (40000 MWCO, ThermoFisherScientific), followed by repeated diafiltration into PBS using an Amicon-Ultra centrifugal filter (10000 MWCO, Merck Millipore).

Preparation of α-EGFR×α-HER2 Fab Dimers

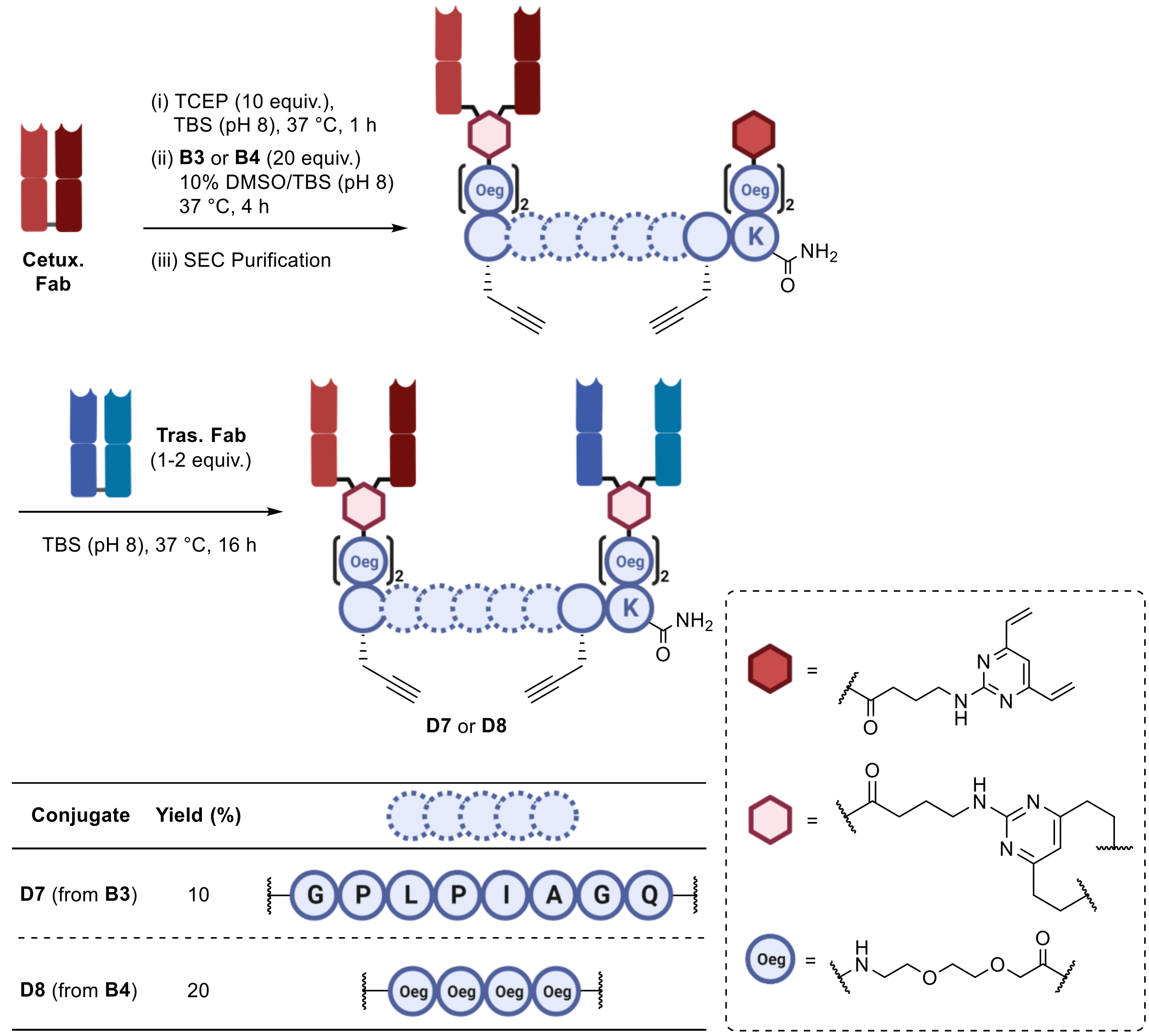

A solution of trastuzumab Fab in TBS (25 mM Tris HCl, 25 mM NaCl, 0.5 mM EDTA, pH 8) was reduced according to General Method C, diluted with TBS to approximately 2.5 mg/mL, linker **B3** or **B4** (10 equiv., 10 mM in DMSO) added, and the DMSO content adjusted to 10% through addition of DMSO. The mixture was vortexed briefly, then incubated at 37 °C for 4 h. The monomer was isolated *via* size-exclusion chromatography (eluting with TBS), concentrated, and a solution of reduced trastuzumab Fab (1-2 equiv.) was added, the solution vortexed, and incubated for 37 °C for 16 h. The bispecific dimer (**D7** or **D8**) was isolated in 10-20% yield *via* size-exclusion chromatography (eluting with PBS).

Preparation of α-EGFR×α-HER2 Alexa Fluor® 488 conjugates

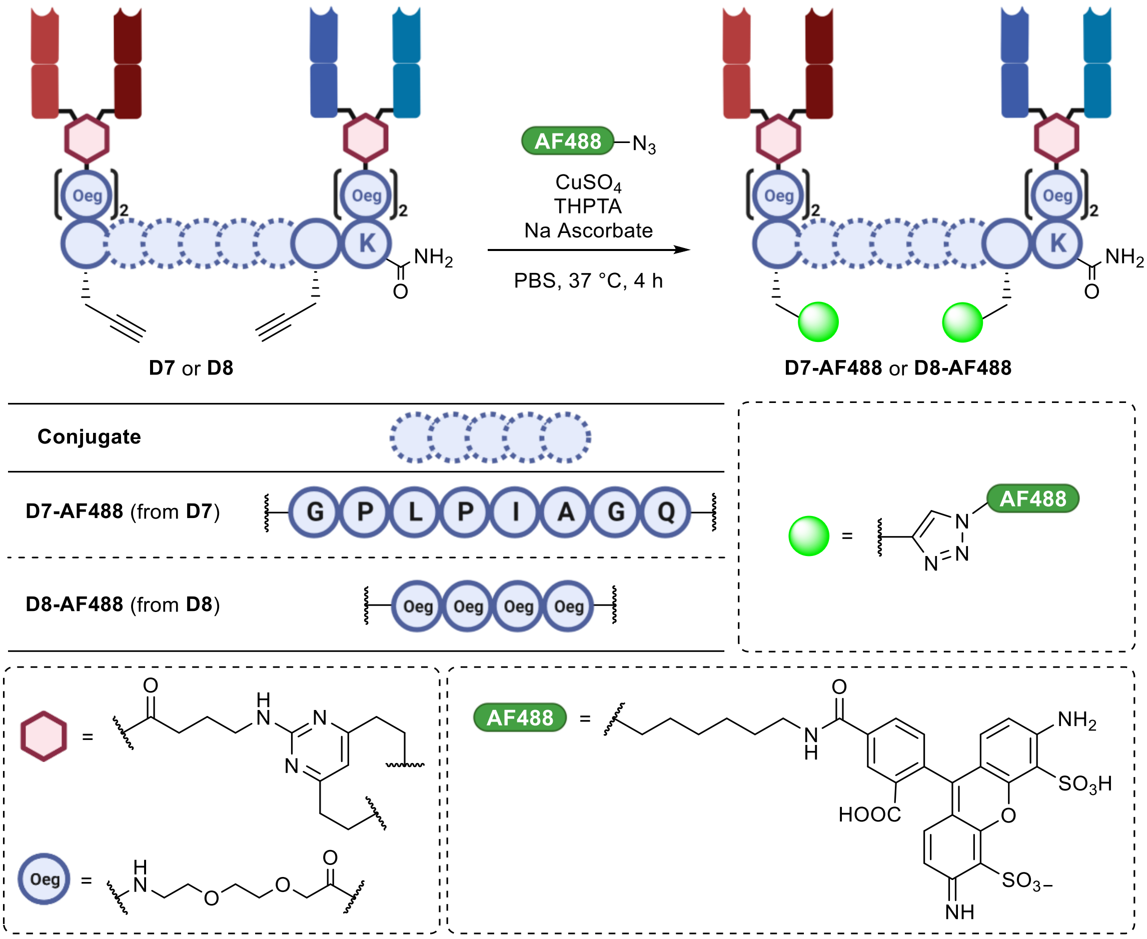

To a solution of conjugate **D7** or **D8** in PBS was added THPTA (400 equiv.), $CuSO_4{\cdot}5H_2O$ (100 equiv.), sodium ascorbate (300 equiv.) and Alexa Fluor® 488 (5 mM in DMSO, 50 equiv.). The DMSO content was adjusted to 10% through addition of DMSO, then the mixture was vortexed and incubated at 37 °C for 6 h. The reaction was quenched with the addition of EDTA (30 min), and the excess reagents were removed by filtration through two successive ZebaSpin Desalting Columns (40000 MWCO, ThermoFisherScientific), followed by repeated diafiltration into PBS using an Amicon-Ultra centrifugal filter (10000 MWCO, Merck Millipore).

Preparation of α-EGFR×α-HER2 ADCs

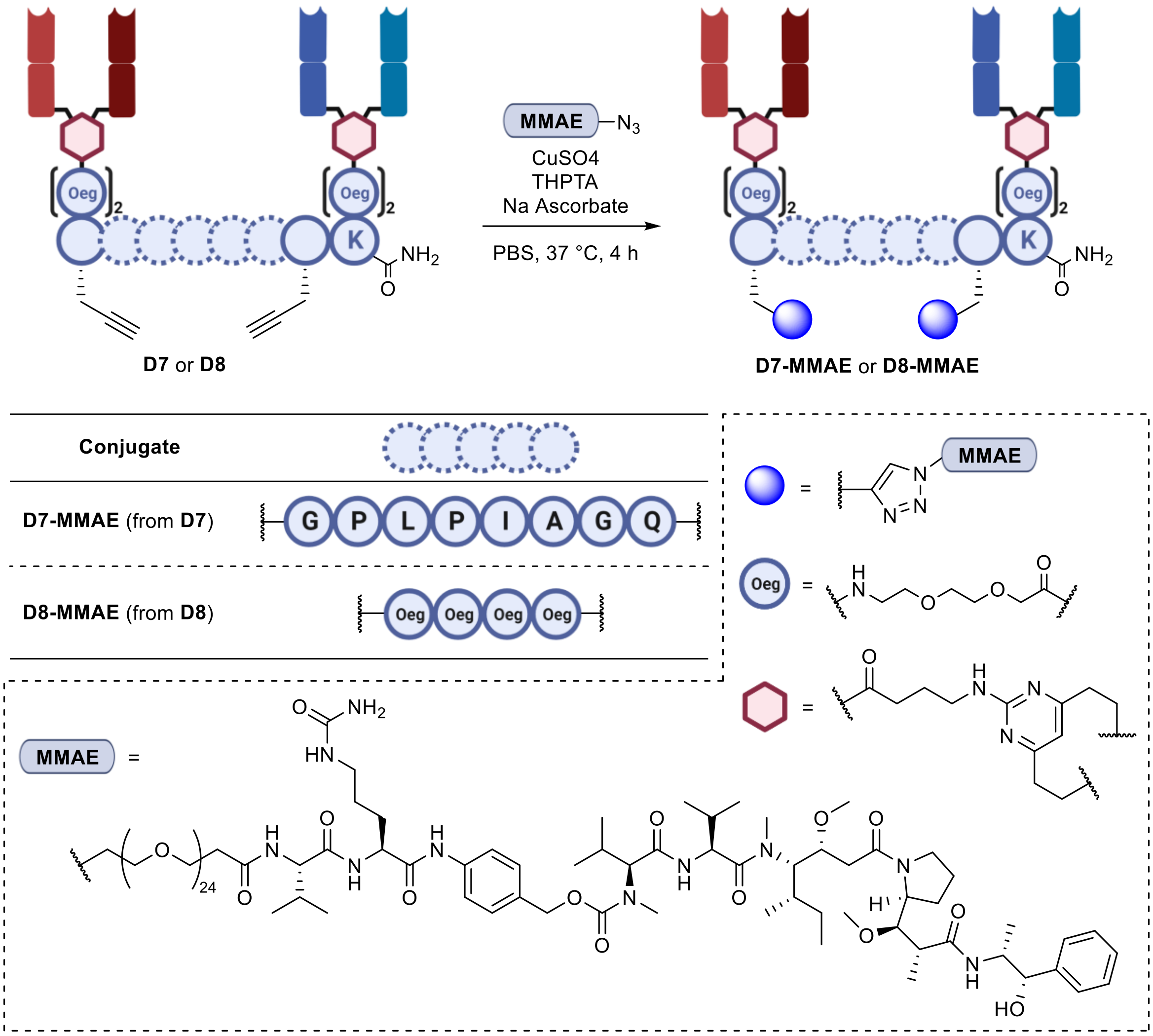

To a solution of conjugate **D7** or **D8** in PBS was added THPTA (400 equiv.), $CuSO_4 \cdot 5H_2O$ (100 equiv.), sodium ascorbate (300 equiv.) and N_3-PEG_{24}-Val-Cit-PABC-MMAE (20 mM in DMSO, 50 equiv.). The DMSO content was adjusted to 10% through addition of DMSO, then the mixture was vortexed and incubated at 37 °C for 5 h. The reaction was quenched with the addition of EDTA (30 min), and the excess reagents were removed by filtration through two successive ZebaSpin Desalting Columns (40000 MWCO, ThermoFisherScientific), followed by repeated diafiltration into PBS using an Amicon-Ultra centrifugal filter (10000 MWCO, Merck Millipore).

Preparation of α-PD-L1×α-HER2 Fab Dimers

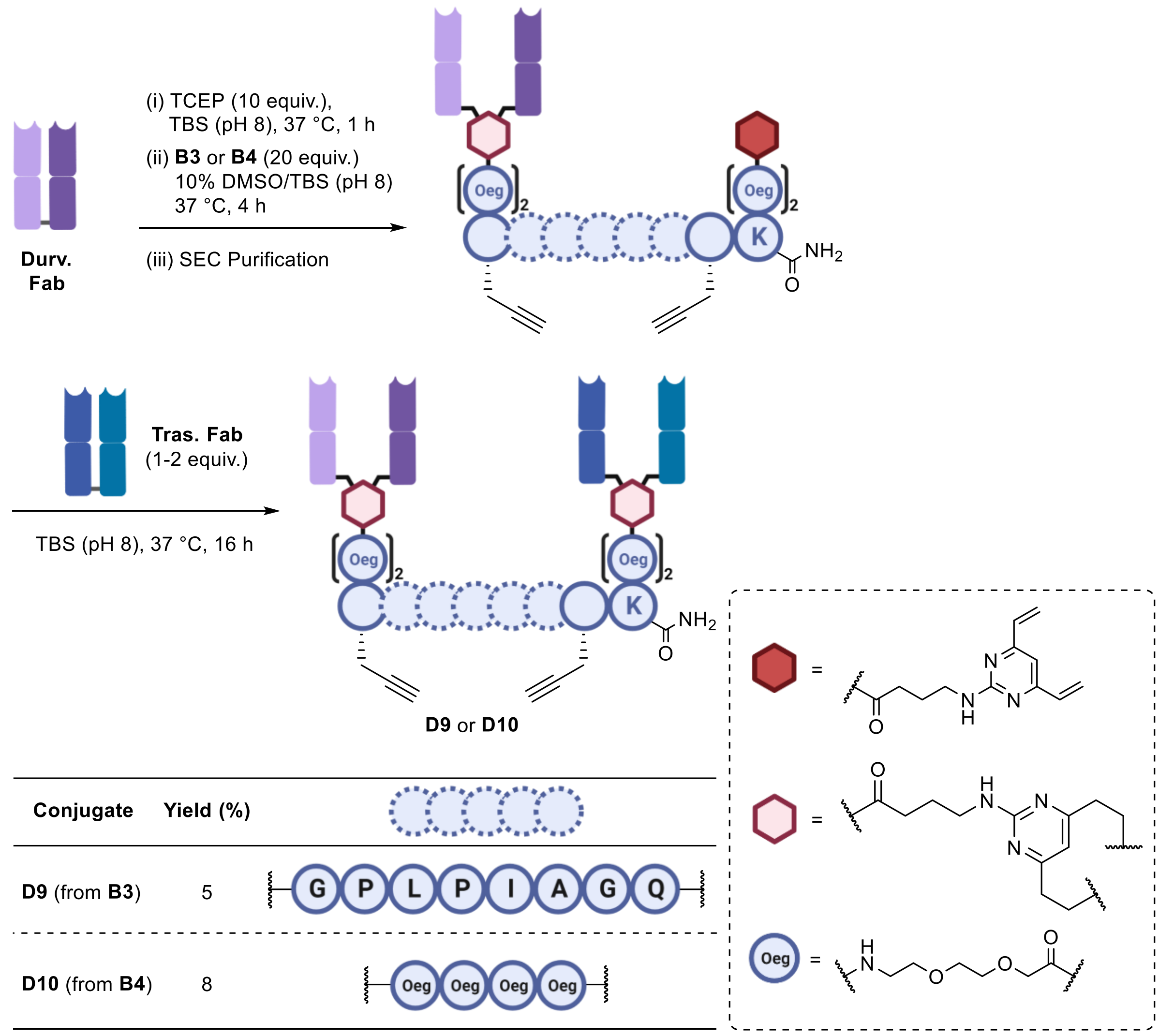

A solution of trastuzumab Fab in TBS (25 mM Tris HCl, 25 mM NaCl, 0.5 mM EDTA, pH 8) was reduced according to General Method C, diluted with TBS to approximately 2.5 mg/mL, linker **B3** or **B4** (10 equiv., 10 mM in DMSO) added, and the DMSO content adjusted to 10% through the addition of DMSO. The mixture was vortexed briefly, then incubated at 37 °C for 16 h. The monomer was isolated *via* size-exclusion chromatography (eluting with TBS), concentrated, and a solution of reduced trastuzumab Fab (1-2 equiv.) was added, the solution vortexed, and incubated for 37 °C for 16 h. The bispecific dimer (**D9** or **D10**) was isolated in 5-8% yield *via* size-exclusion chromatography (eluting with PBS)

1.6 MMP2 Cleavage

Activation of MMP2/MMP9

Latent MMP2 (SinoBiological, Cat. No. 10082-HNAH) or MMP9 (SinoBiological, Cat. No. 10327-NHAH) was reconstituted according to the supplier's instructions, aliquoted, flash frozen in N_2 and stored at -80 °C. To activate, the aliquot was warmed to room temperature, diluted by a factor of 5 with a 1 mM solution of 4-aminophenylmercuric acetate in TCN buffer (50 mM Tris, 150 mM NaCl, 10 mM $CaCl_2$, pH 7.5), and incubated at 37 °C for 1 h.

Cleavage

D1 or **D2** (5 µM) was incubated at 37 °C for 8 hours in TCN buffer (50 mM Tris, 10 mM $CaCl_2$, 150 mM NaCl, pH 7.5) with a selected amount of activated MMP2. Aliquots were taken at 1, 2, 4 and 8 hours, flash frozen in N_2, and stored at -20 °C until analysis. The aliquots were reduced and analysed by SDS-PAGE. Consumption of the dimer species was observed through gradual disappearance of the corresponding band on the SDS-PAGE gel.

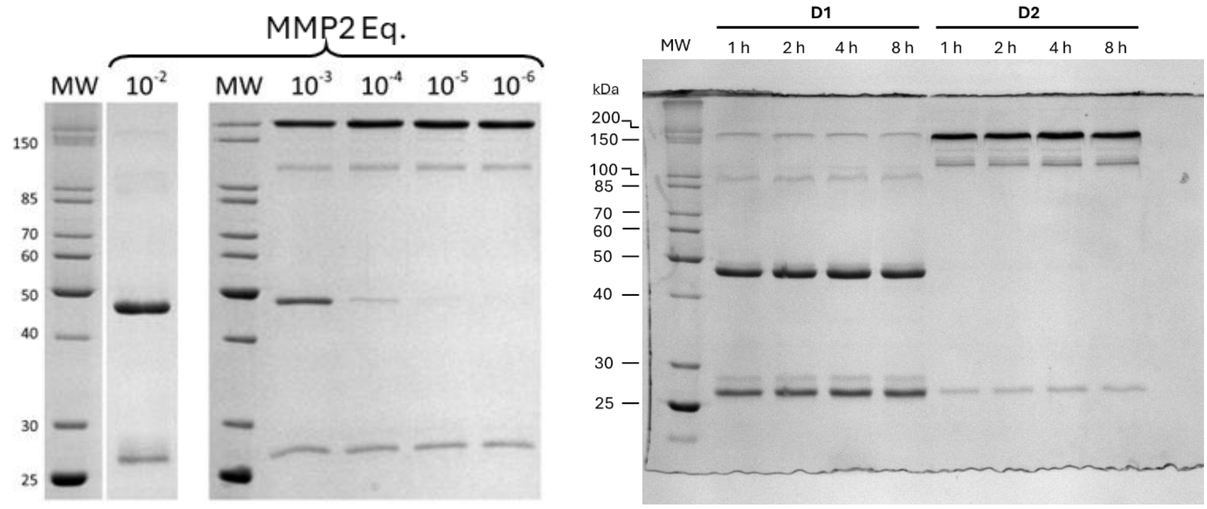

Figure S3: SDS-PAGE analysis of incubation of **D1** and **D2** (5 µM) with activated MMP2 (50 nM). All lanes run under reducing conditions, with preheating at 95 °C for 5 min. SDS-PAGE conditions: 12% SDS, 200 V, 65 min. Left: effect of different MMP2 equivalents on the cleavage of **D1**. Right: uncropped gel from Figure 2C.

1.7 Fluorimetry and DAR analysis

Fluorescence was measured with a Pherastar FS plate reader, or ClarioStar plate reader (λ_{ex} = 488 nm, λ_{em} = 535 nm). A 100 µL solution of **D5-FRET** or **D6-FRET** (final concentration 10 nM) and optionally, activated MMP2 or MMP9 (final concentration 35 µM), and optionally, (2R)-2-[(4-biphenylylsulfonyl)amino]-3-phenylpropionic acid (final concentration 100 µM; MMP2/MMP9 Inhibitor I, Calbiochem) was added to a 98 well plate (Greiner, black, clear bottomed) and fluorescence intensity measured over either 1.5 or 16 h at 37 °C with readings taken at 1-3 minute intervals. An adhesive film (Bio-Rad) was used to prevent solvent evaporation. The reactions were performed in triplicate, and the plotted values are the mean values.

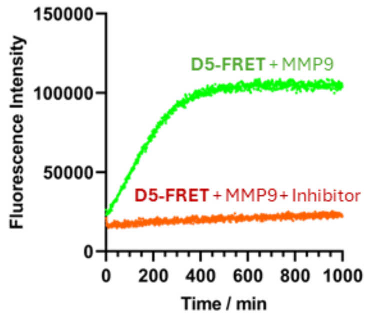

Figure S4: Fluorometric measurement of Alexa Fluor® 488 quenching in dimer **D5-FRET**; Incubation with MMP9 in the presence (orange) and absence (green) of the MMP inhibitor (2R)-2-[(4-biphenylylsulfonyl)amino]-3- phenylpropionic acid; λex = 495 nm, λem = 535 nm. Each point is the average of three technical replicates.

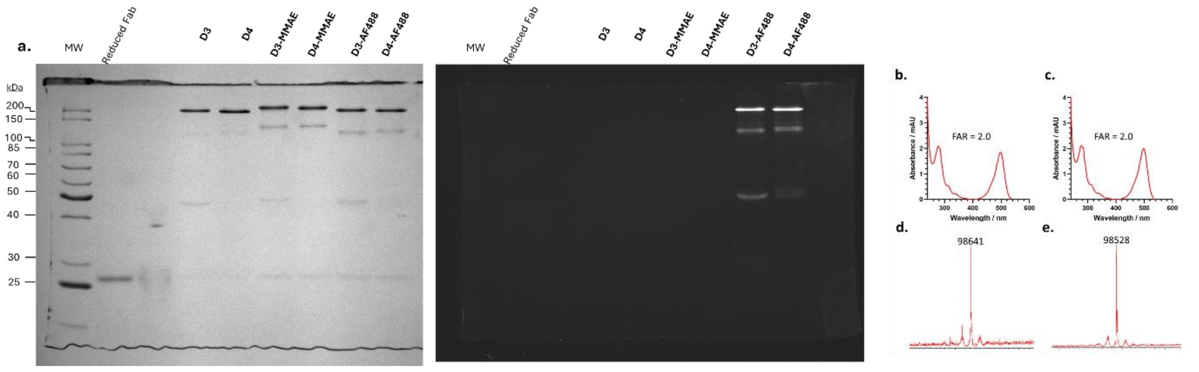

Figure S5: (a) SDS-PAGE analysis of **D3-AF488** and **D4-AF488**. Lanes: MW = molecular weight marker. Coomassie stain (left) and in-gel fluorescence (right); (b) average UV-vis spectrum of **D3-AF488** (three independent measurements); (c) average UV-vis spectrum of **D4-AF488** (three independent measurements); (d) deconvoluted MS of **D3-AF488**, observed 98641 Da, expected 98631 Da; (e) deconvoluted MS of **D4-AF488**, observed 98528 Da, expected 98519 Da. Lanes marked * are not relevant for this work.

BisFabs **D3** and **D4** each contain two alkyne handles, and so it was expected that a CuAAC with Alexa Fluor® 488 azide would, in each case and assuming complete conversion, result in a fluorophore-to-antibody ratio (FAR) of 2. Although, the deconvoluted MS of both products confirmed this to be case, corroboration was sought *via* UV-vis spectroscopic analysis (Figure S5b-c). In both cases, the FAR was determined to be 2.0 according to the following equation:

$$FAR = \frac{Abs_{495}/\varepsilon_{495}}{(Abs_{280} - 0.11 \times Abs_{495})/\varepsilon_{280}}$$

where

$$\varepsilon_{495} = 71000\ \mathrm{M^{-1}\ cm^{-1}}$$
$$\varepsilon_{280} = 142255\ \mathrm{M^{-1}\ cm^{-1}}$$

A correction factor of 0.11 was applied to account for absorbance at 280 nm due to Alexa Fluor® 488. The extinction coefficient at 280 nm for the Fab dimer was estimated from the summation of the extinction coefficients of its constituent parts: namely, trastuzumab Fab (68590) and conjugated-'divinylpyrimidine' (2540).[7]

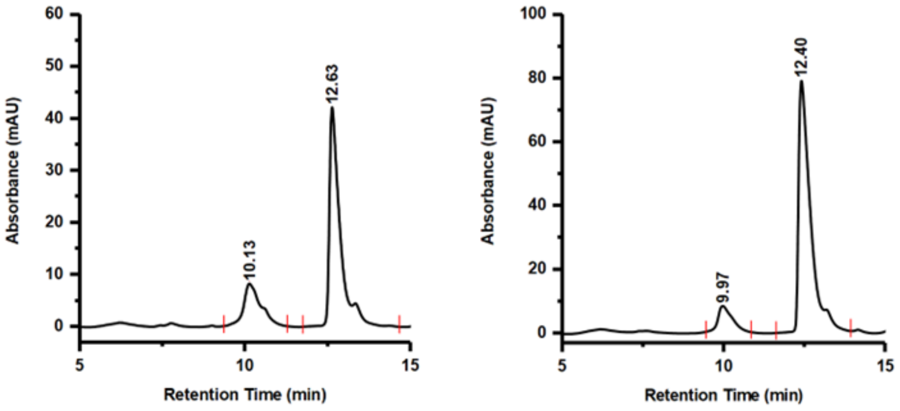

Figure S6: HIC analysis of CuAAC between: Left; **D3** and 100 equiv. of N_3-PEG24-Val-Cit-PABC-MMAE (DAR = 1.8); and right; **D7** and 100 equiv. of N_3-PEG24-Val-Cit-PABC-MMAE (DAR = 1.9).

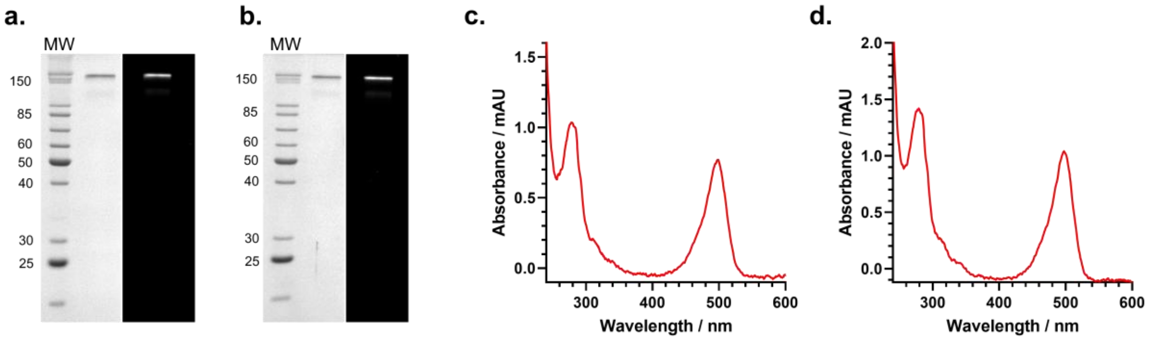

Figure S7: SDS-PAGE analysis of Alexa Fluor® 488 functionalised α-EGFR×α-HER2 BisFabs: (a) **D7-AF488** and (b) **D8-AF488**. Lanes: MW = molecular weight marker; 1 = **D7-AF488**; 4 = **D8-AF488**. Coomassie stain (left) and in-gel fluorescence (right) shown. SDS-PAGE conditions: 12% SDS, 200V, 65 min. Average UV-vis spectra of (c) **D7-AF488** and (d) **D8-AF488** (three independent measurements).

1.8 BisFab Plasma Stability

To a solution of conjugate **D3-AF488**, **D4-AF488**, **D7-AF488**, or **D8-AF488** in PBS (final concentration 1 μM) was added reconstituted human plasma (Sigma, final concentration 10% v/v). The mixture was incubated at 37 °C for 4 days. Aliquots were removed at 0, 1, 2, 3, and 4 days, flash frozen in N_2 and stored at -80 °C until analysis. SDS-PAGE was followed by in-gel fluorescence imaging and Coomassie brilliant blue staining and imaging.

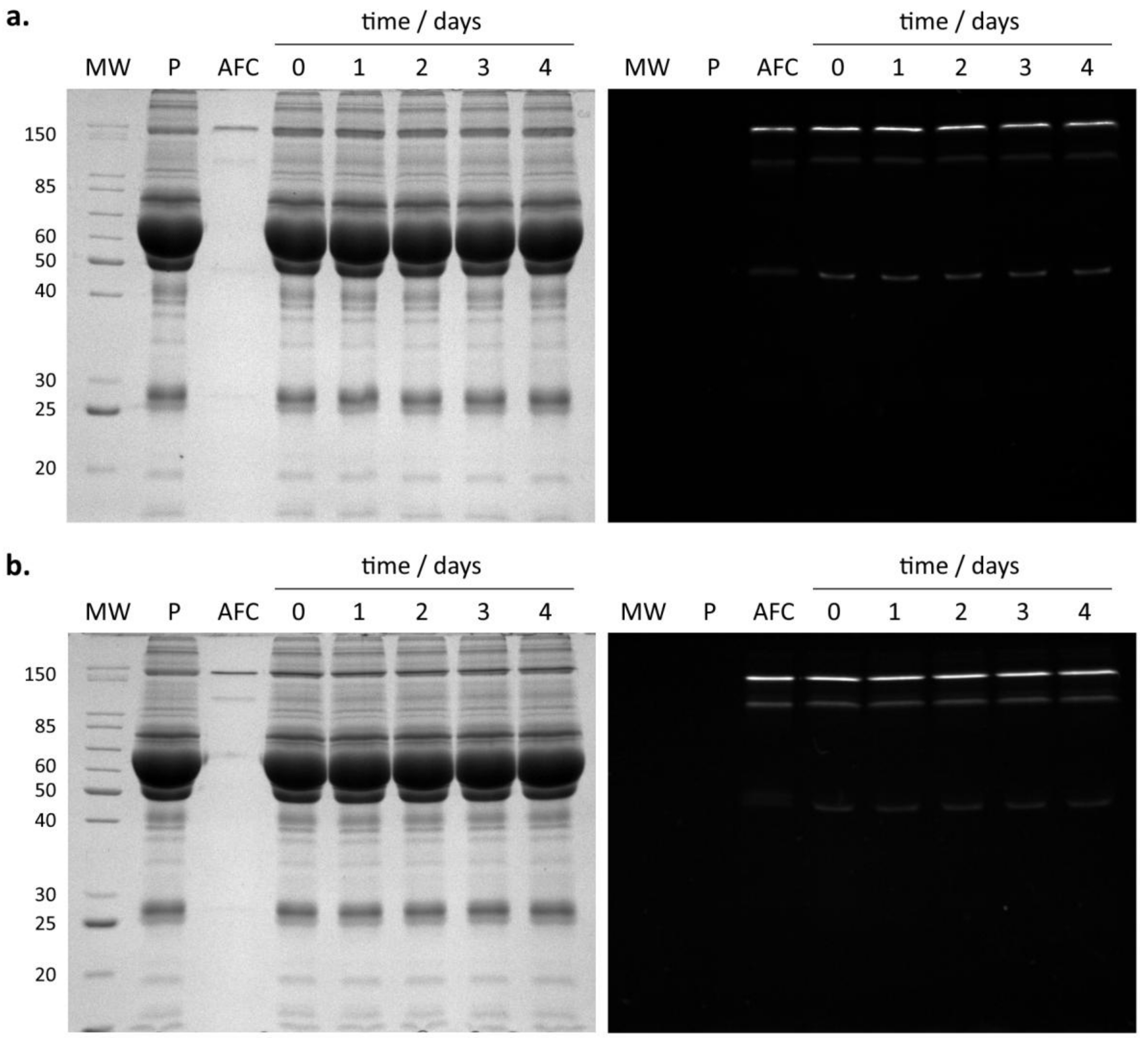

Figure S8: Stability analysis of Alexa Fluor® 488-conjugates (a) **D3-AF488** and (b) **D4-AF488** by SDS-PAGE. Lanes: MW = molecular weight marker; P = human plasma; AFC = either (a) **D3-AF488**, or (b) **D4-AF488**; {0, 1, 2, 3, 4} = days of incubation. Coomassie stain (left) and in-gel fluorescence (right) prior to staining are depicted. No evidence of significant loss or transfer of Alexa Fluor® 488 to plasma proteins was observed over 4 day period.

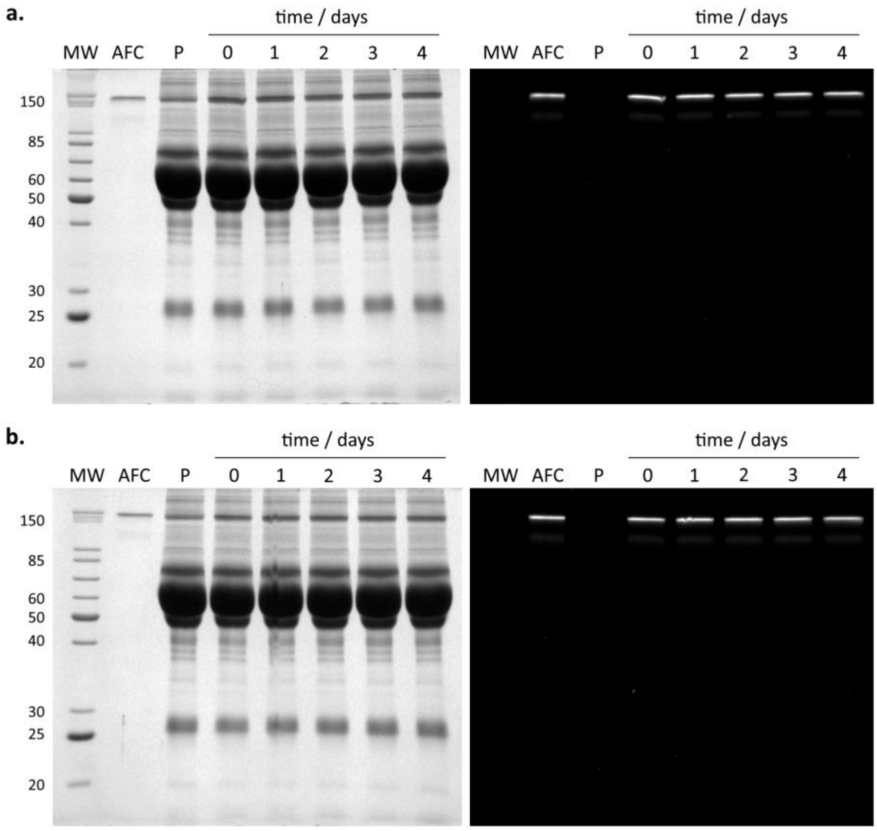

Figure S9: Stability analysis of Alexa Fluor® 488-conjugates: (a) **D7-AF488**, and (b) **D8-AF488** by SDS-PAGE. Lanes: MW = molecular weight marker; P = human plasma; AFC = either (a) **D7-AF488**, or (b) **D8-AF488**; {0, 1, 2, 3, 4} = days of incubation. Coomassie stain (left) and in-gel fluorescence (right) prior to staining are depicted. No evidence of significant loss or transfer of Alexa Fluor® 488 to plasma proteins was observed over the 4 day period.

1.9 Enzyme-linked immunosorbent assay (ELISA)

Indirect ELISA

A 96-well plate was coated with 100 µL of a 0.25 µg/mL solution of HER2 (Sino Biological, His-tagged) overnight at 4 °C. The coating solution was removed and each well washed with PBS (3 × 200 µL). Each well was then blocked with 1% BSA in PBS (200 µL) for 1 h at room temperature. The blocking solution was removed, and each well washed with PBS (3 × 200 µL). The wells were then treated with 100 µL of Trastuzumab IgG, Trastuzumab Fab, **D3-MMAE**, **D4-MMAE** at the following concentrations in PBS, prepared by serial dilution: 90 nM, 30 nM, 10 nM, 3.33 nM, 1.11 nM, 0.37 nM, 0.12 nM. Control wells were coated with 100 µL of PBS. The plate was incubated for 2 h at room temperature. The conjugate solutions were then removed, and each well washed with 0.1% Tween 20 in PBS (3 × 200 µL), followed by PBS (3 × 200 µL). 100 µL of detection antibody (1:5000 dilution in PBS, goat anti-human IgG-HRP, SigmaAldrich) was added to each well, and the plate incubated for 1 h at room temperature. Each well was then washed with 0.1% Tween 20 in PBS (3 × 200 µL), followed by PBS (3 × 200 µL), and 100 µL of an OPD solution (1 capsule of OPD in 9 mL H2O and 1 mL of stable peroxide buffer (10x), Thermo Fisher) added to each well. After approximately 15 minutes, 50 µL of 4 M HCl was added to each well to quench the reaction. The absorbance at 490 and 590 nm was measured with a CLARIOstar microplate reader. Measurements were performed in triplicate, with error bars representing the standard deviation. Three independent repeats were performed.

Sandwich ELISA

A 96-well plate was coated with 100 µL of a 0.25 µg/mL solution of HER2 (Sino Biological, His-tagged) for 1 h at room temperature. The coating solution was removed and each well washed with PBS (3 × 200 µL). Each well was then blocked with 1% BSA in PBS (200 µL) overnight at 4 °C. The blocking solution was removed, and each well washed with PBS (3 × 200 µL). The wells were then treated with 100 µL of Trastuzumab Fab, Cetuximab Fab, Durvalumab Fab, **D7-MMAE**, or **D8-MMAE** at the following concentrations in PBS, prepared by serial dilution: 90 nM, 30 nM, 10 nM, 3.33 nM, 1.11 nM, 0.37 nM, 0.12 nM. Control wells were coated with 100 µL of PBS. The plate was incubated for 2 h at room temperature. The conjugate solutions were then removed, and each well washed with 0.1% Tween 20 in PBS (3 × 200 µL), followed by PBS (3 × 200 µL). 100 µL of either biotinylated human EGFRvIII (0.2 µg/mL in PBS, AcroBiosystems) or biotinylated human PD-L1 (0.2 µg/mL in PBS, AcroBiosystems) was added to each well and the plate incubated for 1 h at room temperature. Each well was then washed with PBS (3 × 200 µL), and 100 µL of ExtrAvidin-peroxidase (5 µL in 10 mL of PBS) added to each well, and the plate incubated overnight at 4 °C. Each well was then washed with 0.1% Tween 20 in PBS (3 × 200 µL), followed by PBS (3 × 200 µL), and 100 µL of an OPD solution (1 capsule of OPD in 9 mL H_2O and 1 mL of stable peroxide buffer (10x), Thermo Fisher) added to each well. After approximately 15 minutes, 50 µL of 4 M HCl was added to each well to quench

the reaction. The absorbance at 490 and 590 nm was measured with a CLARIOstar microplate reader. Measurements were performed in triplicate, with error bars representing the standard deviation. Two independent repeats were performed.

1.10 Cell-based assays

Cell lines

HER2-positive SKBR3 and BT474 cells were obtained from the American Type Culture Collection (ATCC) and HER2-negative MCF7 and MDA-MB-468 cells were obtained from the European Collection of Authenticated Cell Cultures (ECACC) and ATCC, respectively. SKBR3 cells were maintained in high glucose McCoy's 5A medium, supplemented with 10% heat inactivated foetal-bovine serum (FBS), GlutaMAX™, 50 U/mL penicillin and 50 µg/mL streptomycin. MCF7 and MDA-MB-468 cells were maintained in Dulbecco's Modified Eagle Medium (DMEM) supplemented with 10% FBS, 2 mM L-glutamine, 50 U/mL penicillin and 50 µg/mL streptomycin. BT474 cells were maintained in RPMI1640 medium supplemented with 10% FBS, 2 mM L-glutamine, 50 U/mL penicillin and 50 µg/mL streptomycin. All cell lines were incubated at 37 °C with 5% CO_2.

Cell viability

Cells were seeded in 96-well plates for 24 h at 37 °C with 5% CO_2. SKBR3 cells were seeded at 15,000 cells/well, BT474 cells were seeded at 20,000 cells/well, MCF7 cells were seeded at 7,500 cells/well and MDA-MB-468 cells were seeded at 10,000 cells/well. Serial dilutions of **D3**, **D4**, **D3-MMAE**, **D4-MMAE**, **D7-MMAE**, **D8-MMAE**, cetuximab Fab or trastuzumab Fab were added to the cells in complete growth medium and incubated at 37 °C with 5% CO_2 for 96 h. Cell viability was determined using a CellTiter-Glo viability assay (Promega) according to the manufacturer's instructions. Cell viability was plotted as a percentage of that of untreated cells. Each measurement was taken in triplicate. Three biologically independent replicates were performed.

Live Cell Microscopy

SKBR3, BT474, MDA-MB-468, or MCF7 cells were seeded at 40,000 cells/well in 8-well chambered µ-slide (Ibidi, 80826) for 48 h at 37 °C with 5% CO_2. Slides were then placed on ice and washed with Ham's F12 Nutrient Mix media containing 10% FBS, 2 mM L-glutamine, 50 U/mL penicillin and 50 µg/mL streptomycin (3 × 200 µL). Antibody-fluorescent-conjugate **D3-AF488**, **D4-AF488**, **D7-AF488**, or **D8-AF488** (50 nM) were added to the cells in complete F12 growth medium and incubated at 4 °C in the dark for 30 min. Cells were placed back on ice and washed with complete F12 growth medium (3 × 200 µL). Complete F12 growth medium (200 µL) was added, and the cells incubated for either 0.5 h or 3.5 h at 37 °C with 5% CO_2, after which Hoechst 33342 trihydrochloride trihydrate (1 µg/mL, Invitrogen, H3570) was added and the cells incubated for a further 0.5 h. Live cell microscopy was performed on an Operetta CLS confocal microscope

(Perkin Elmer) with a 40× water objective. Cells were maintained in a humidified atmosphere at 37 °C and 5% CO_2 throughout analysis. Data analysis was performed using Harmony and ImageJ (Fiji).

Analytical Data

NMR Spectra

Ethyl 4-((4,6-dichloropyrimidin-2-yl)amino)butanoate (8)

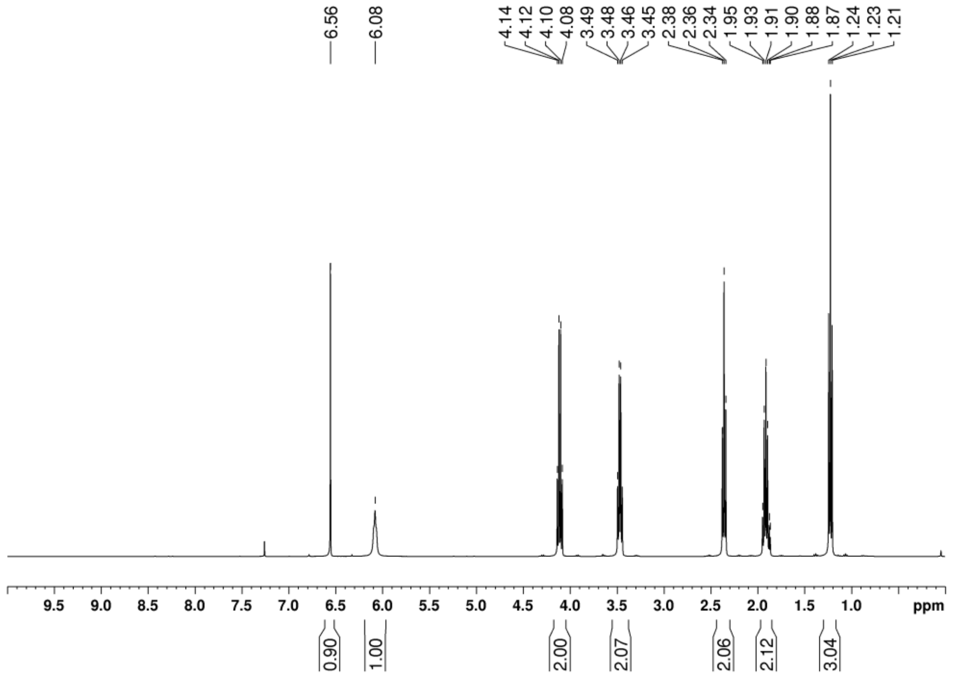

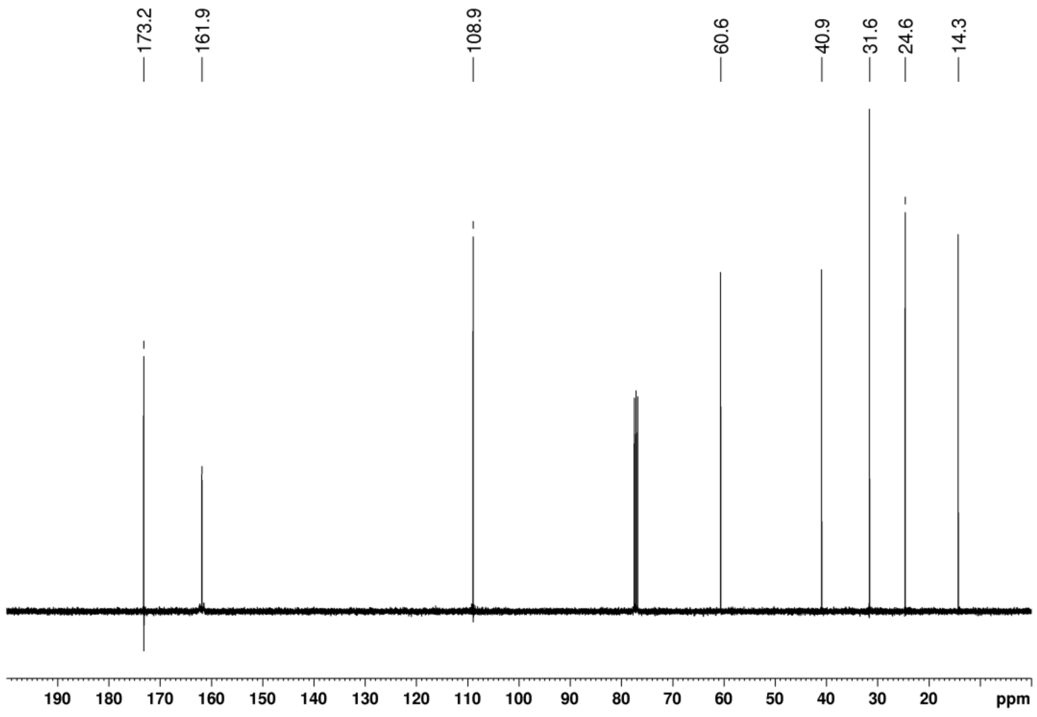

Ethyl 4-((4,6-divinylpyrimidin-2-yl)amino)butanoate (9)

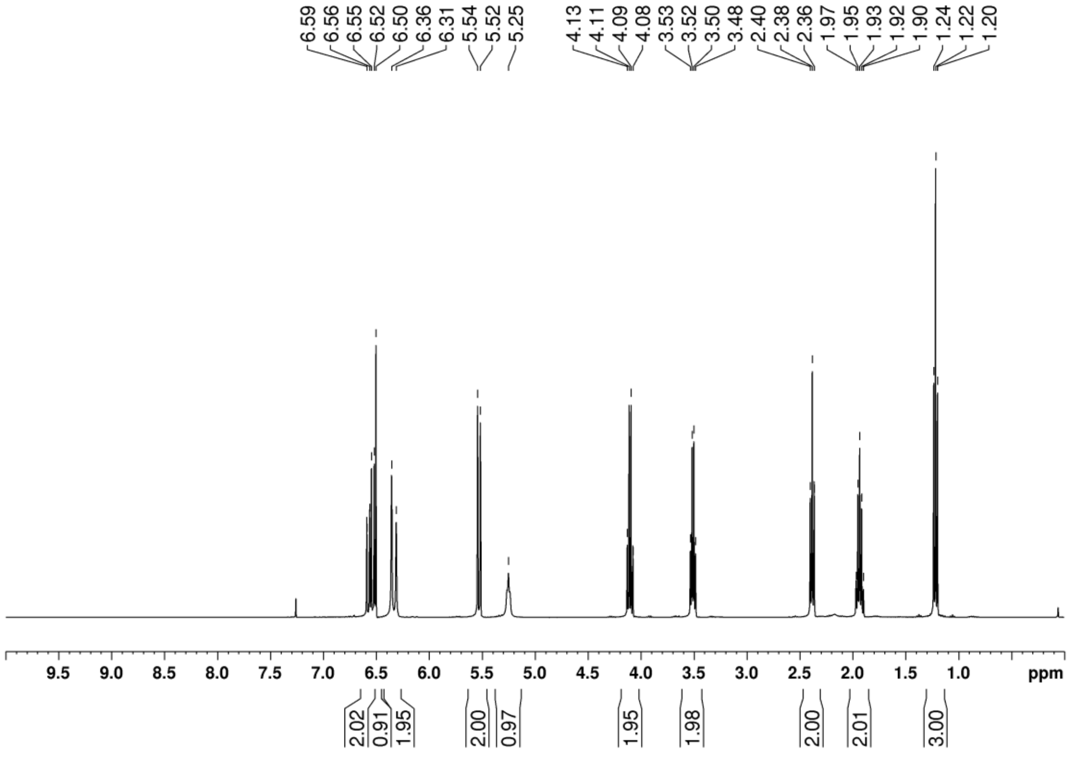

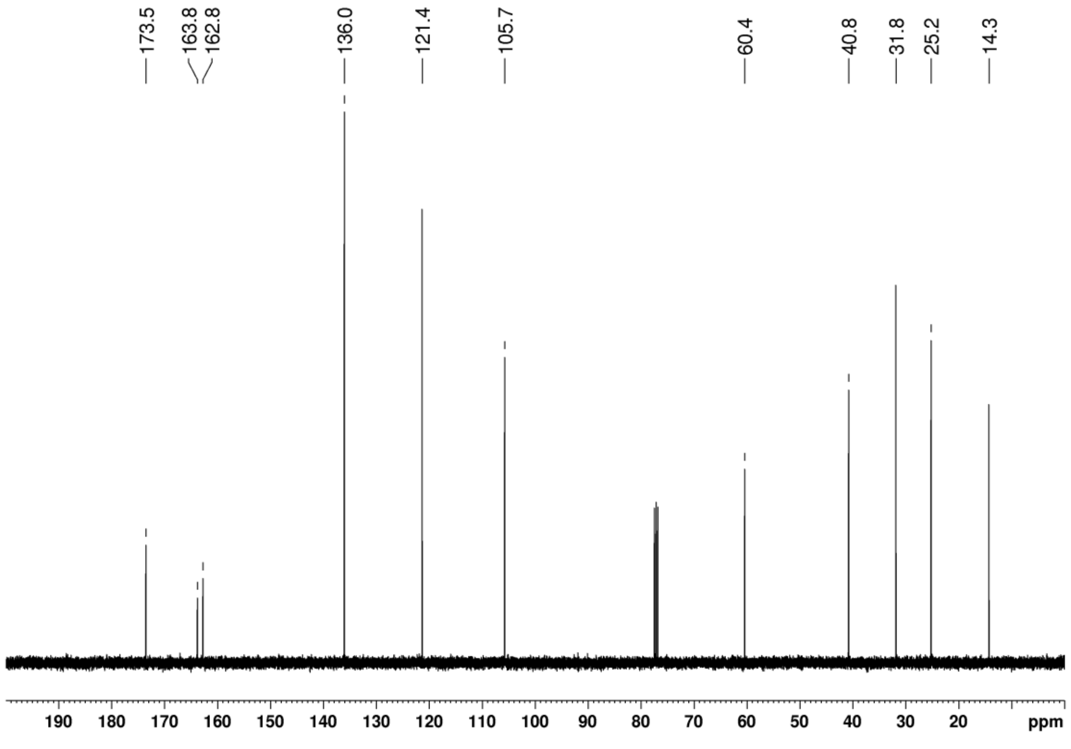

4-((4,6-divinylpyrimidin-2-yl)amino)butanoic acid (10)

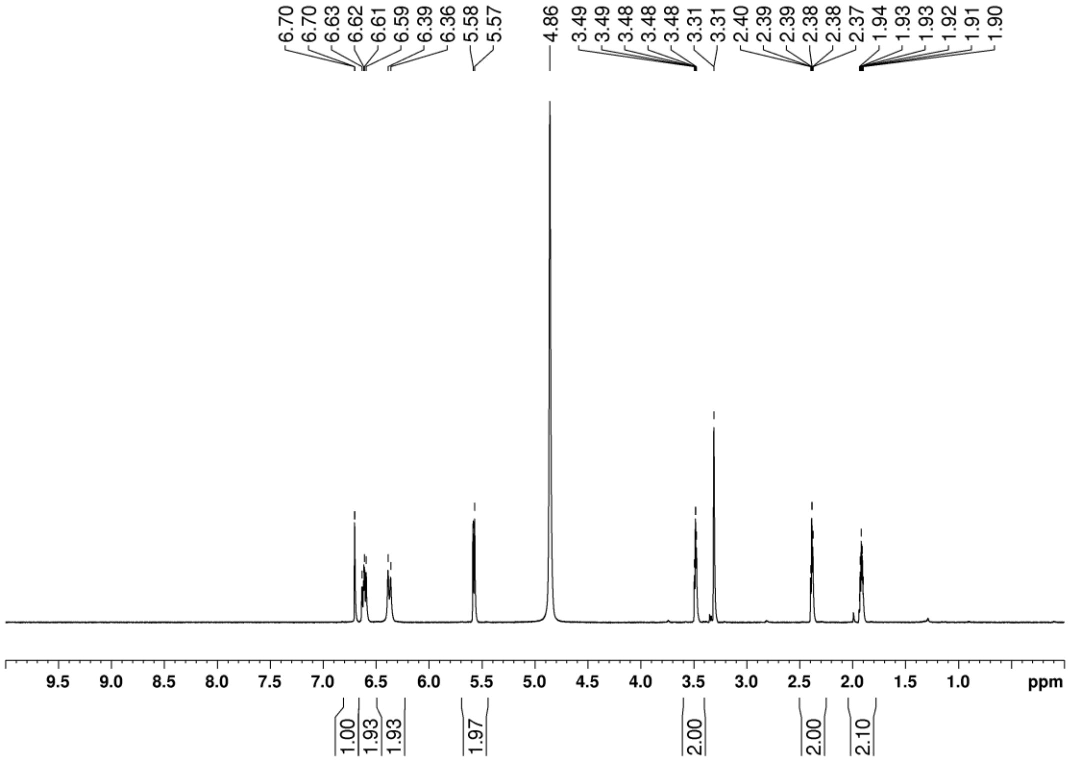

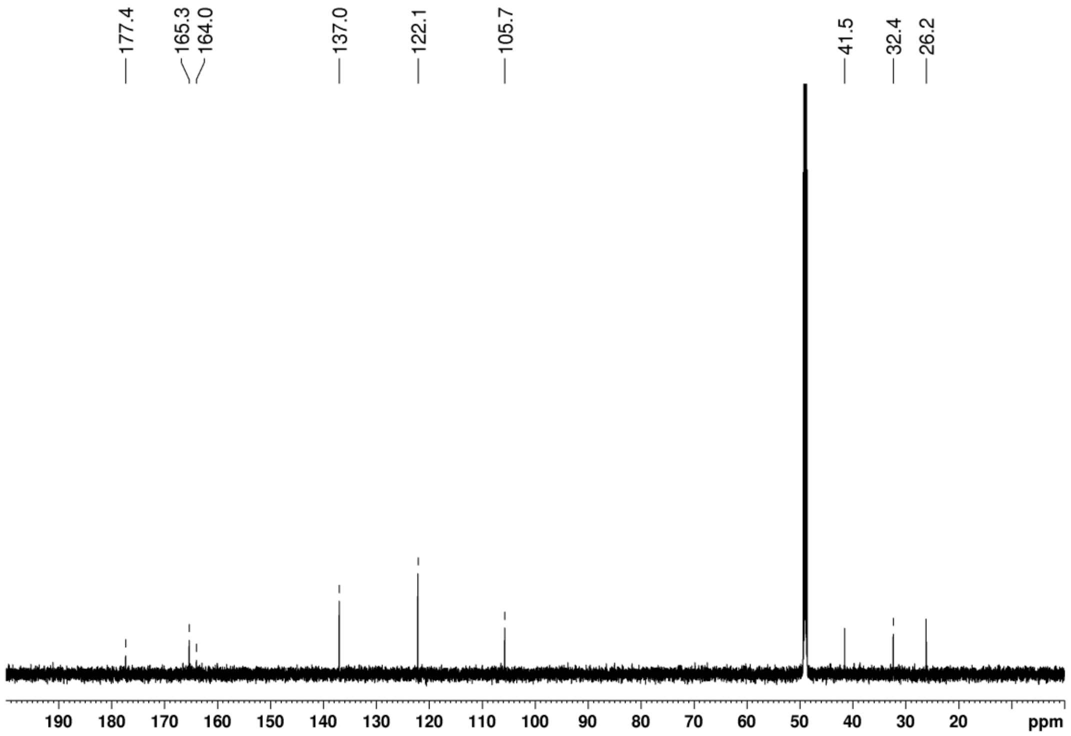

***N*[2]-(((9*H*-fluoren-9-yl)methoxy)carbonyl)-*N*[6]-(1-(4,4-dimethyl-2,6-dioxocyclohexylidene)ethyl)-*L*-lysine (11)**

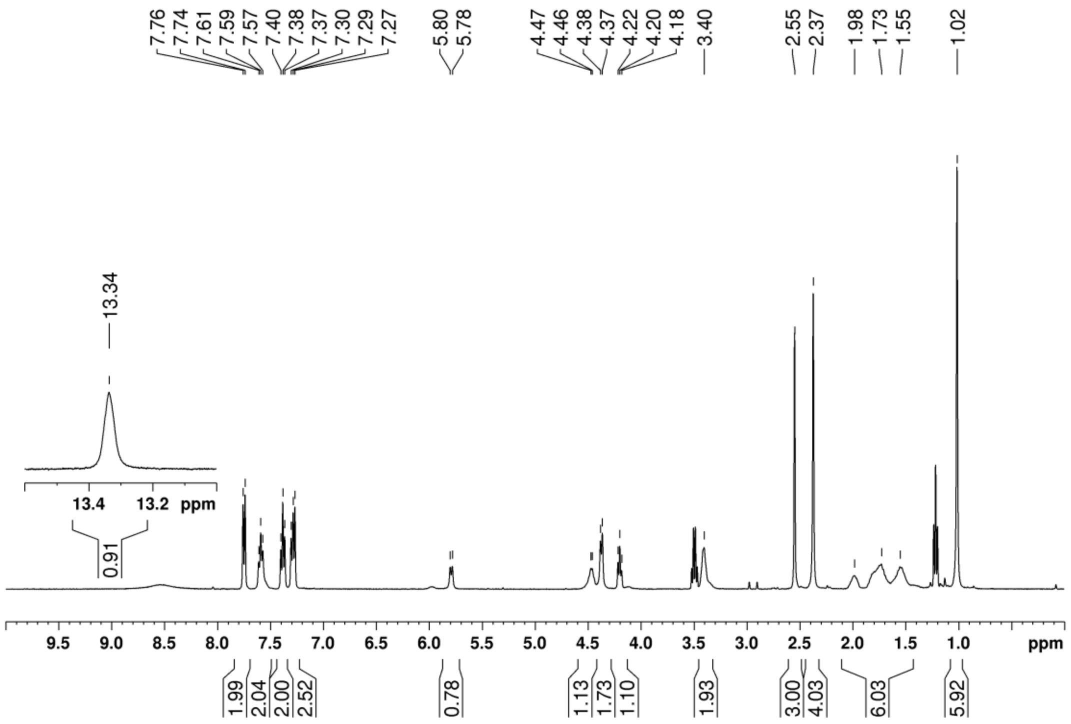

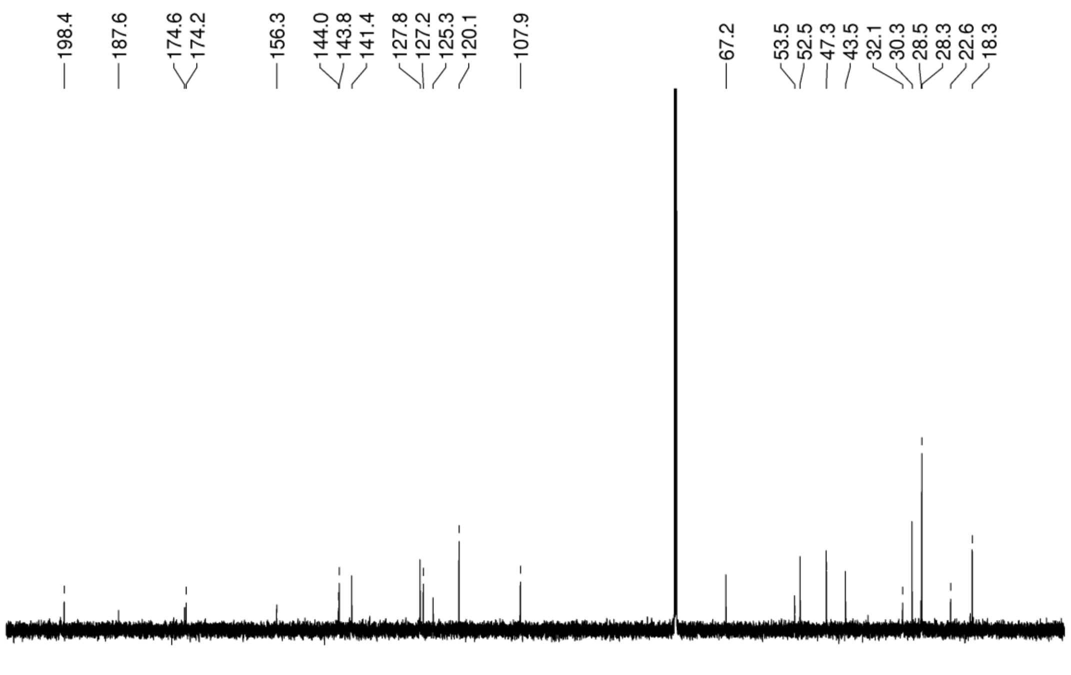

(*S*)-2-((((9*H*-fluoren-9-yl)methoxy)carbonyl)amino)-3-ammoniopropanoate (12)

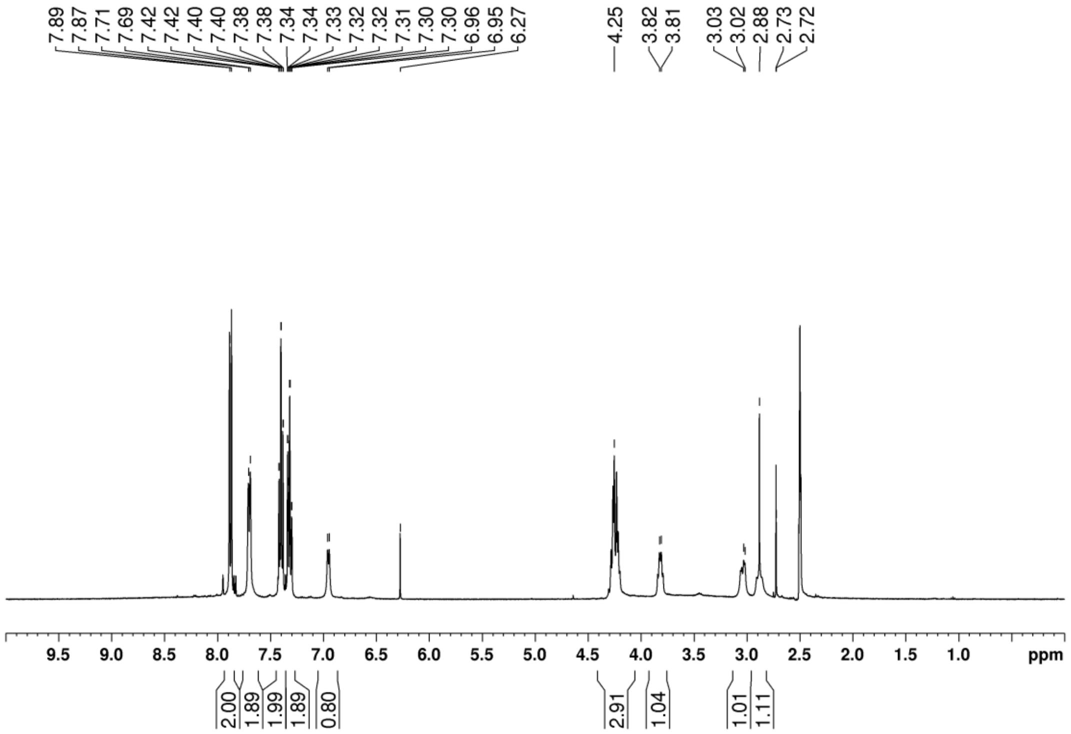

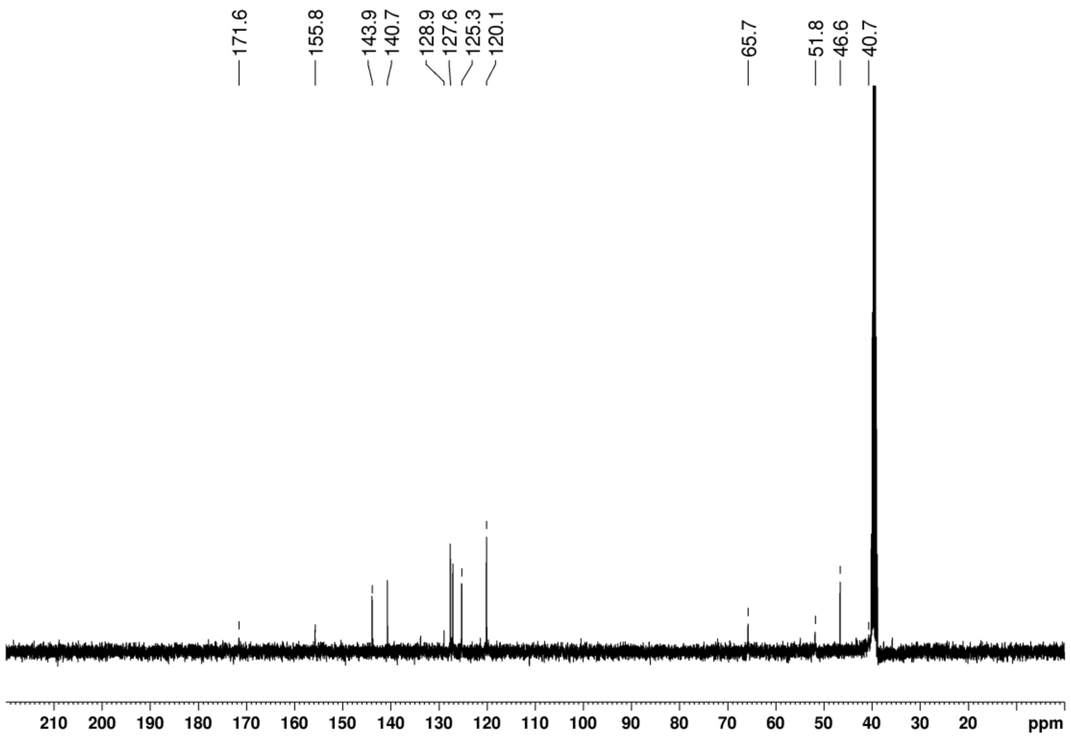

(*S*)-2-((((9*H*-fluoren-9-yl)methoxy)carbonyl)amino)-3-azidopropanoic acid (13)

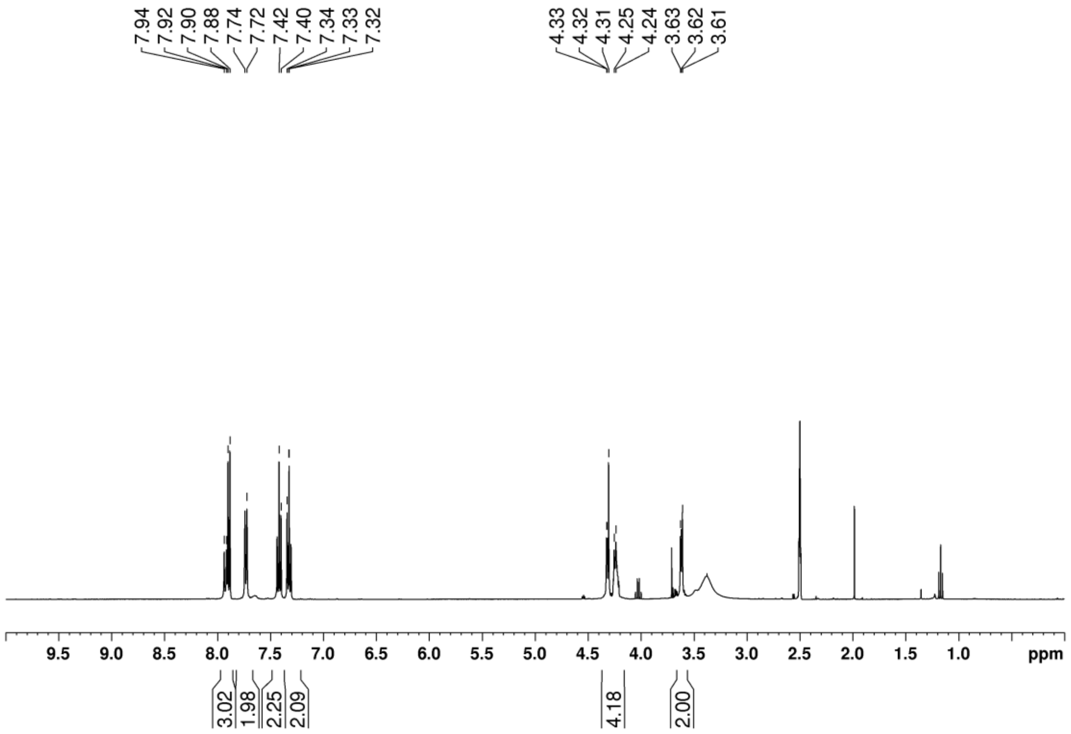

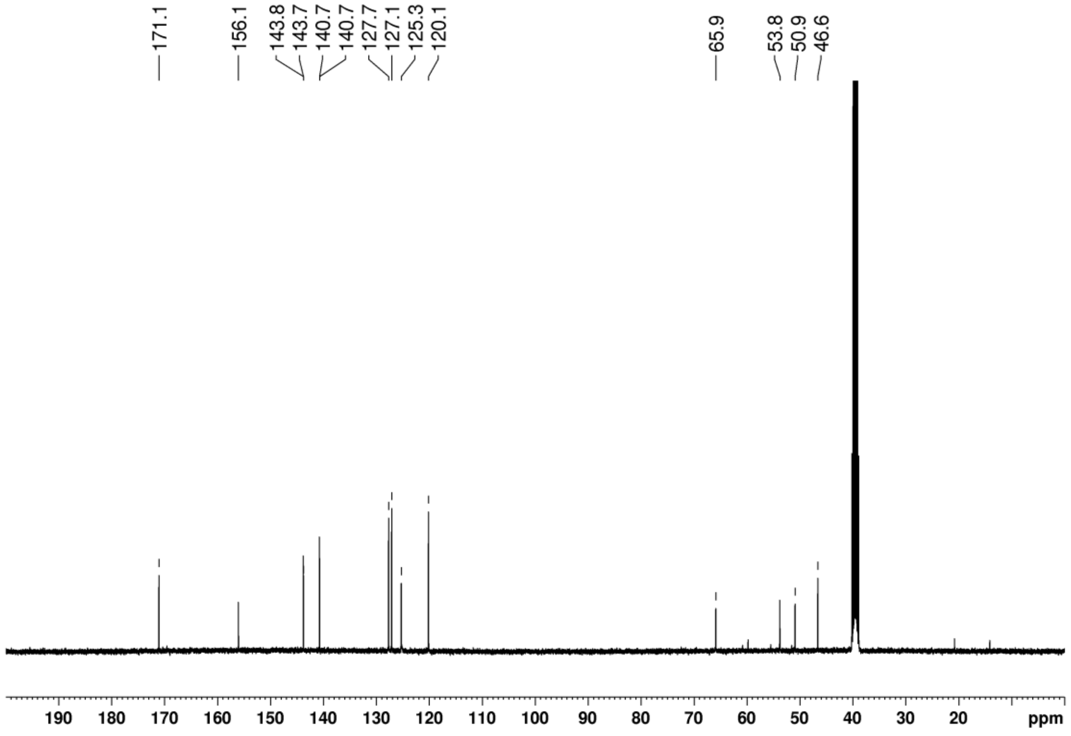

1-(azidosulfonyl)-1*H*-imidazol-3-ium hydrogen sulfate (14)

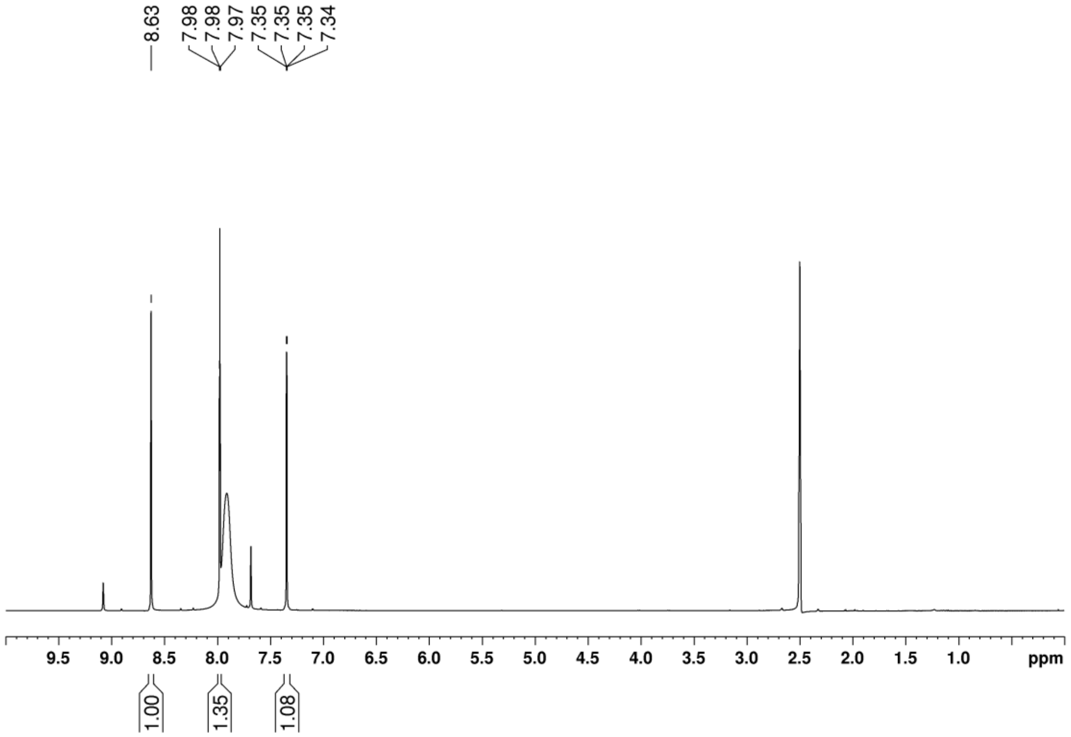

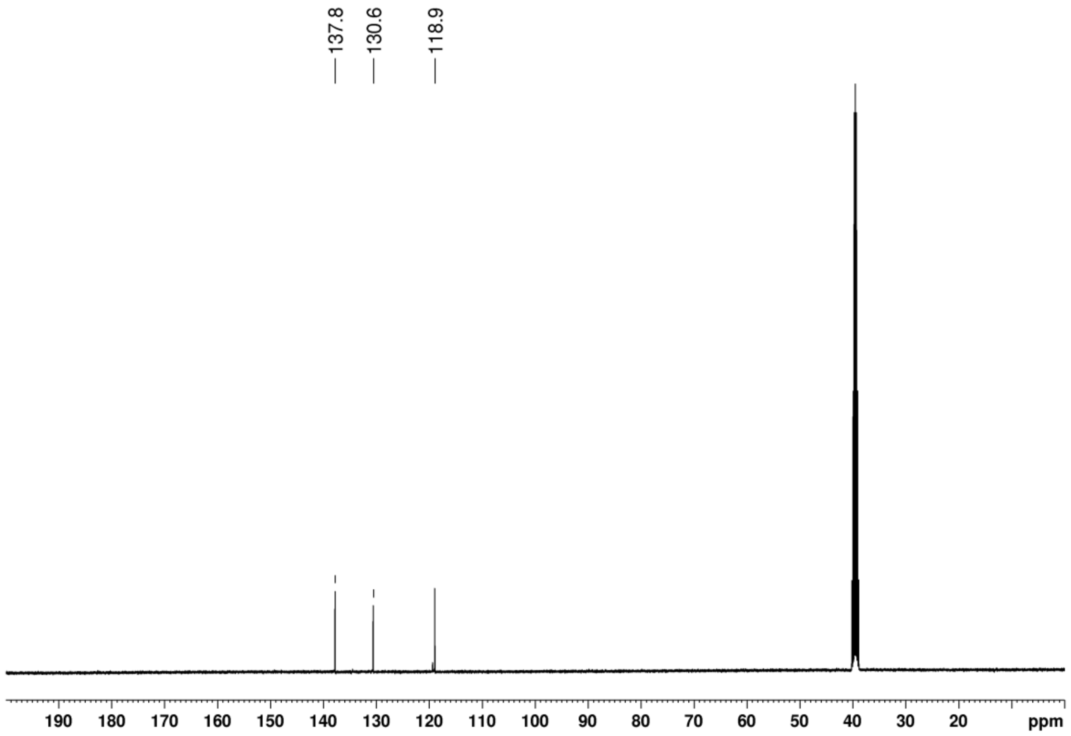

HPLC Traces

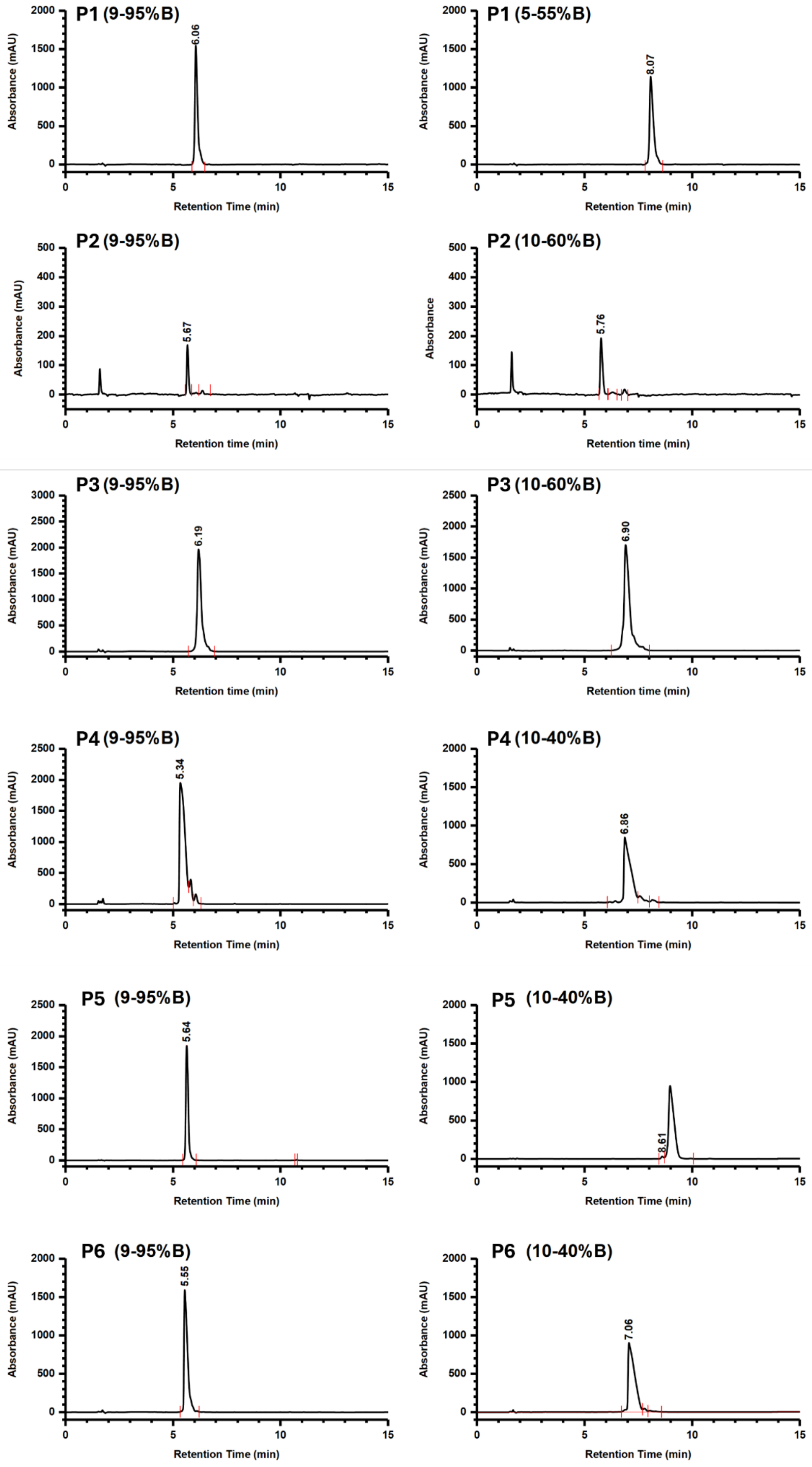
P1 (9-95%B)
6.06
Absorbance (mAU)
Retention Time (min)
P1 (5-55%B)
8.07
Absorbance (mAU)
Retention Time (min)
P2 (9-95%B)
5.67
Absorbance (mAU)
Retention time (min)
P2 (10-60%B)
5.76
Absorbance
Retention time (min)
P3 (9-95%B)
6.19
Absorbance (mAU)
Retention time (min)
P3 (10-60%B)
6.90
Absorbance (mAU)
Retention time (min)
P4 (9-95%B)
5.34
Absorbance (mAU)
Retention Time (min)
P4 (10-40%B)
6.86
Absorbance (mAU)
Retention Time (min)
P5 (9-95%B)
5.64
Absorbance (mAU)
Retention Time (min)
P5 (10-40%B)
8.61
Absorbance (mAU)
Retention Time (min)
P6 (9-95%B)
5.55
Absorbance (mAU)
Retention Time (min)
P6 (10-40%B)
7.06
Absorbance (mAU)
Retention Time (min)

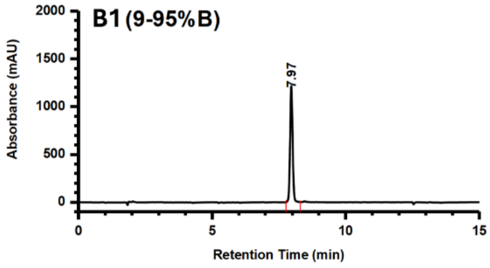
B1 (9-95%B)
7.97
Absorbance (mAU)
Retention Time (min)

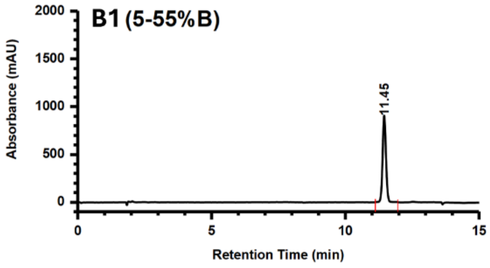
B1 (5-55%B)
11.45
Absorbance (mAU)
Retention Time (min)

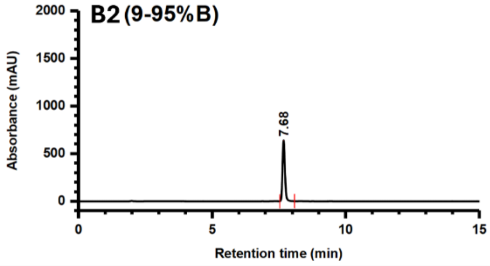
B2 (9-95%B)
7.68
Absorbance (mAU)
Retention time (min)

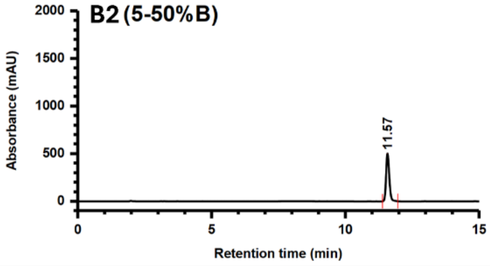
B2 (5-50%B)
11.57
Absorbance (mAU)
Retention time (min)

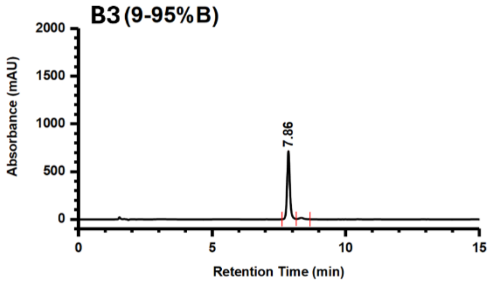
B3 (9-95%B)
7.86
Absorbance (mAU)
Retention Time (min)

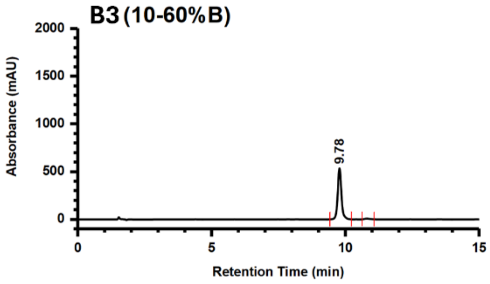
B3 (10-60%B)
9.78
Absorbance (mAU)
Retention Time (min)

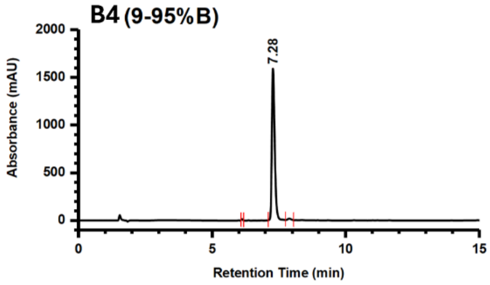
B4 (9-95%B)
7.28
Absorbance (mAU)
Retention Time (min)

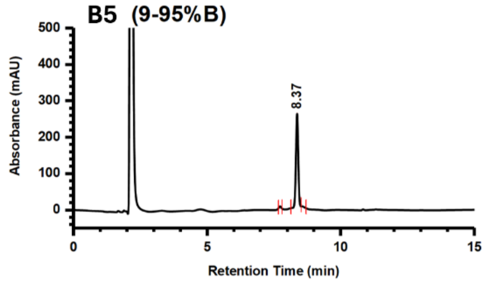
B5 (9-95%B)
8.37
Absorbance (mAU)
Retention Time (min)

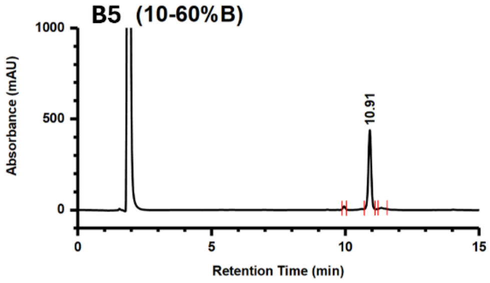
B5 (10-60%B)
10.91
Absorbance (mAU)
Retention Time (min)

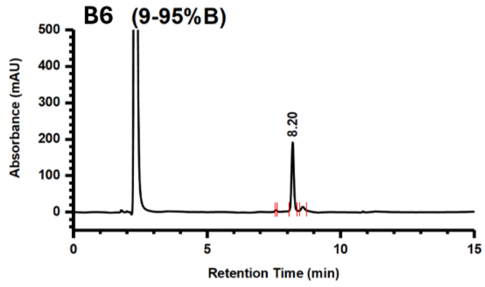
B6 (9-95%B)
8.20
Absorbance (mAU)
Retention Time (min)

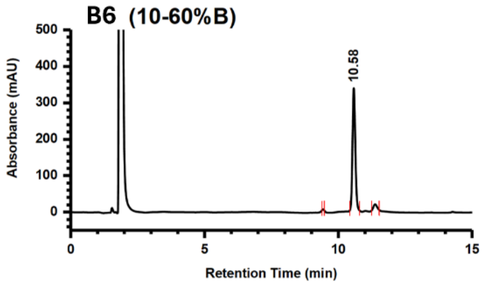
B6 (10-60%B)
10.58
Absorbance (mAU)
Retention Time (min)

Protein LCMS

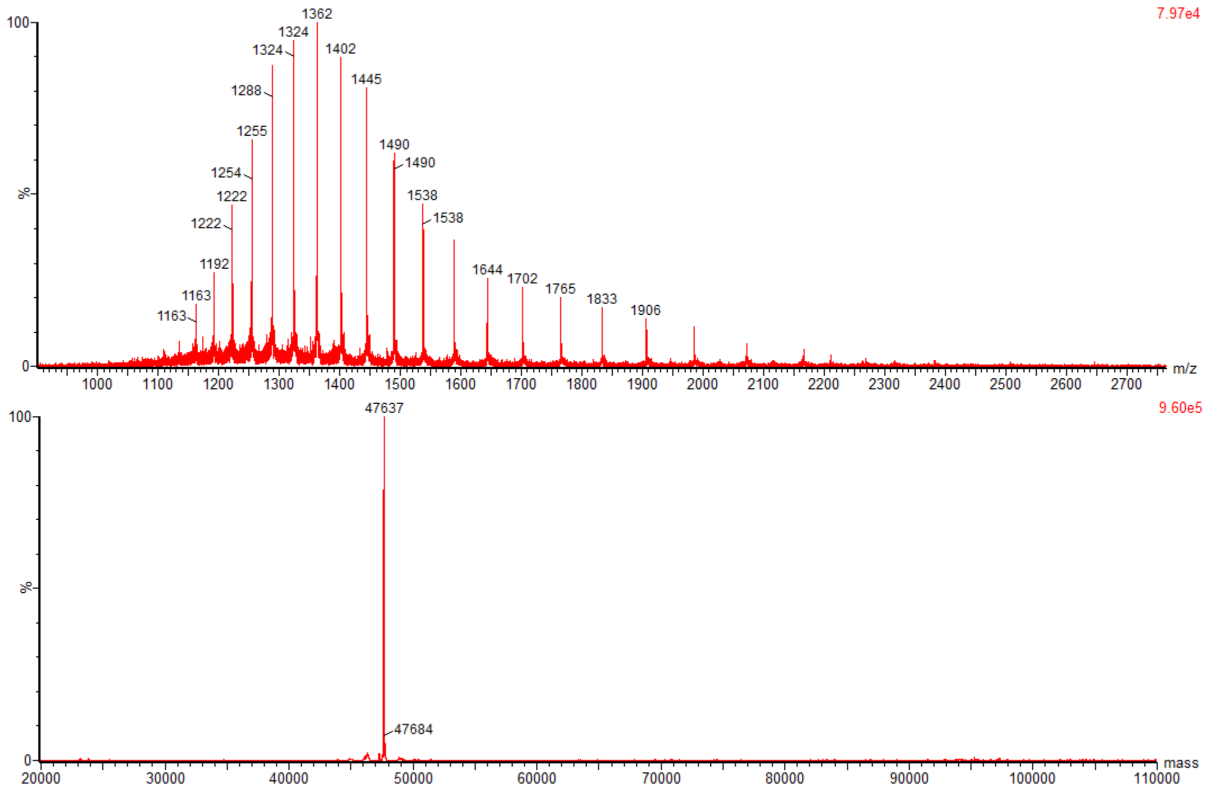

Figure S10: Non-deconvoluted (top) and deconvoluted (bottom) MS of trastuzumab Fab. Expected 47638 Da, observed 47637 Da.

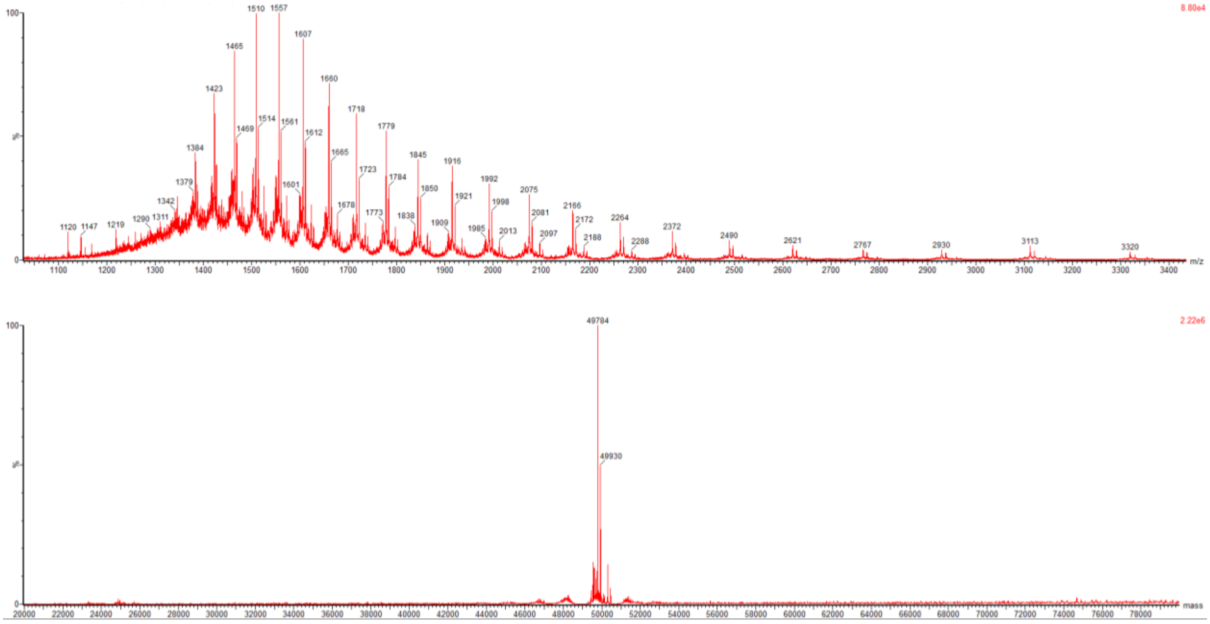

Figure S11: Non-deconvoluted (top) and deconvoluted (bottom) MS of Cetuximab Fab. Observed 49784 Da and 49930 Da (glycosylation).

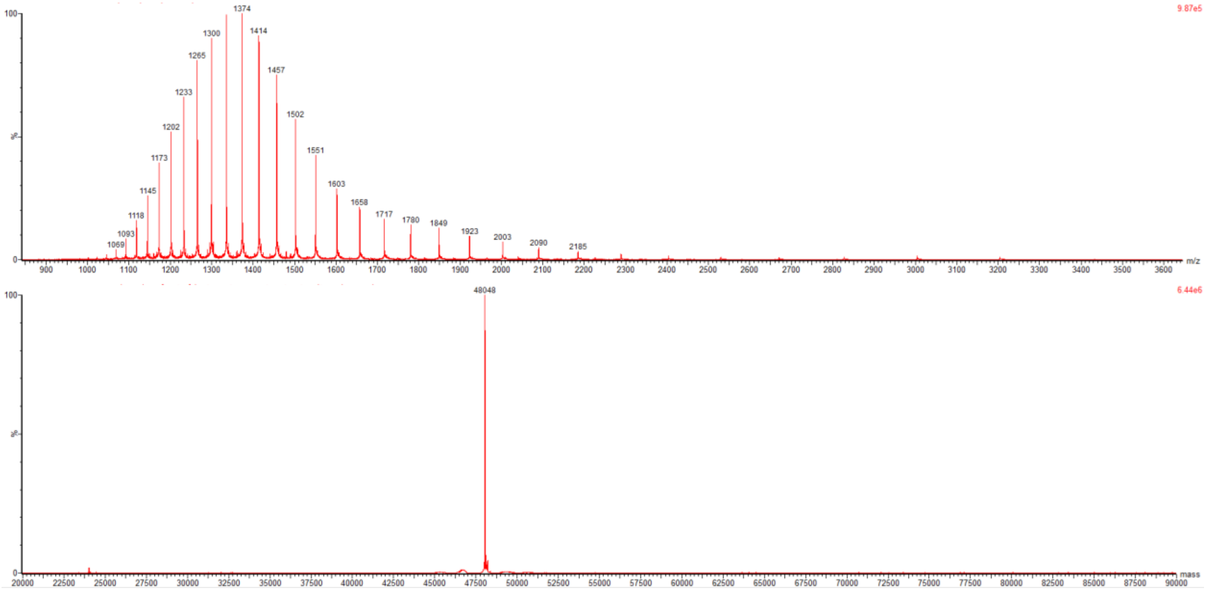

Figure S12: Non-deconvoluted (top) and deconvoluted (bottom) MS of durvalumab Fab. Expected 48046 Da, observed 48048 Da.

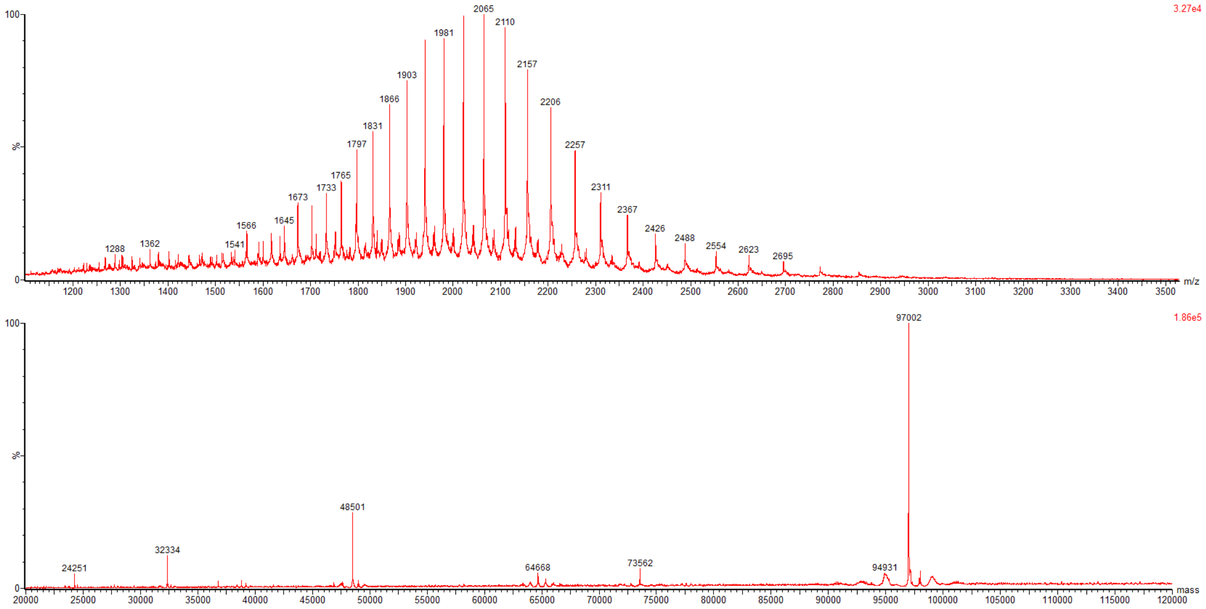

Figure S13: Non-deconvoluted (top) and deconvoluted (bottom) MS of conjugate **D1**. Expected 96998 Da, observed 97002 Da.

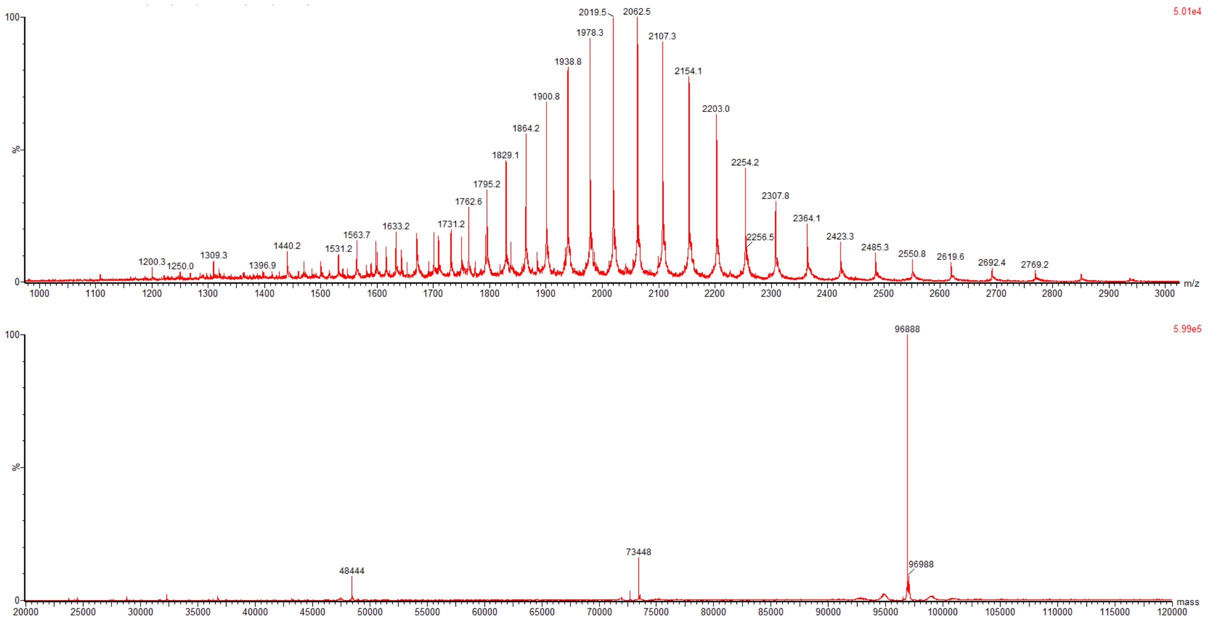

Figure S14: Non-deconvoluted (top) and deconvoluted (bottom) MS of conjugate **D2**. Expected 96884 Da, observed 96888 Da.

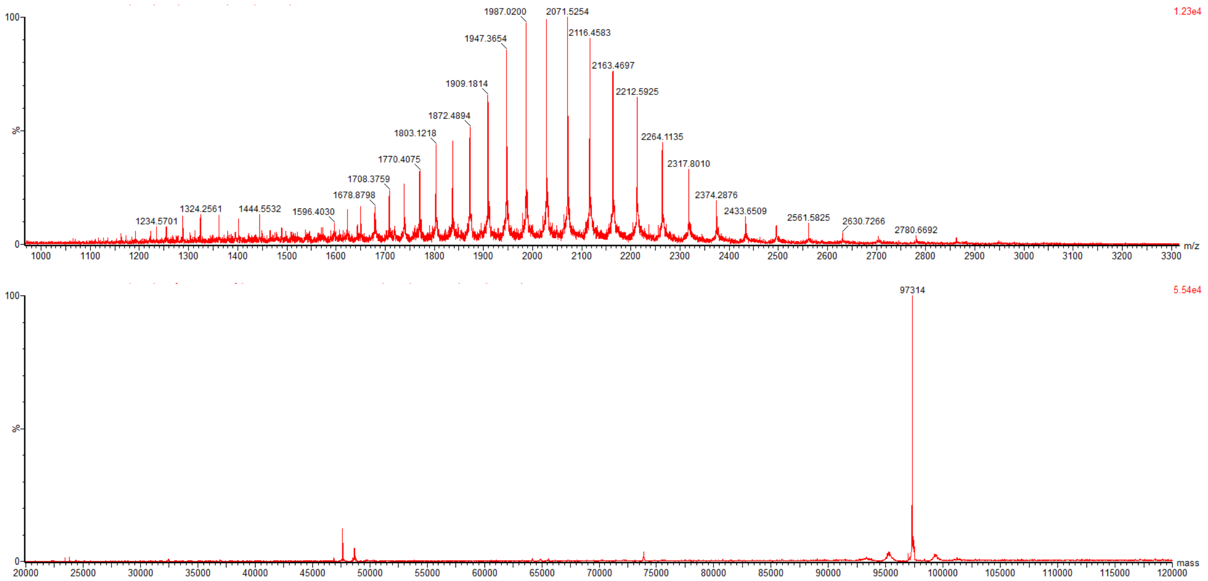

Figure S15: Non-deconvoluted (top) and deconvoluted (bottom) MS of conjugate **D3**. Expected 97315 Da, observed 97314 Da.

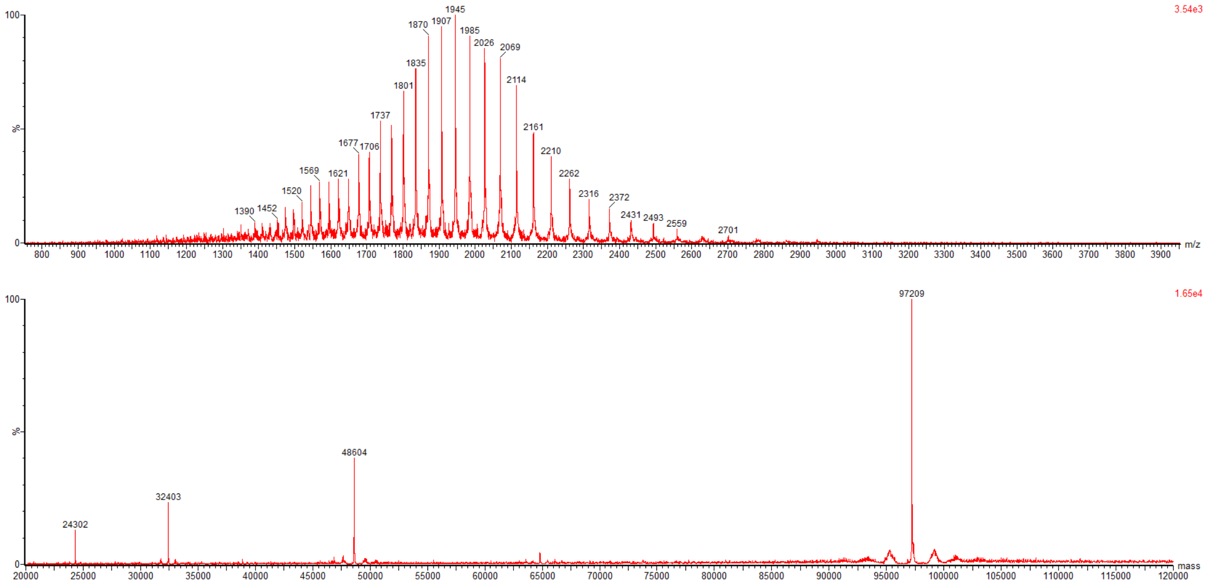

Figure S16: Non-deconvoluted (top) and deconvoluted (bottom) MS of conjugate **D4**. Expected 97203 Da, observed 97209 Da.

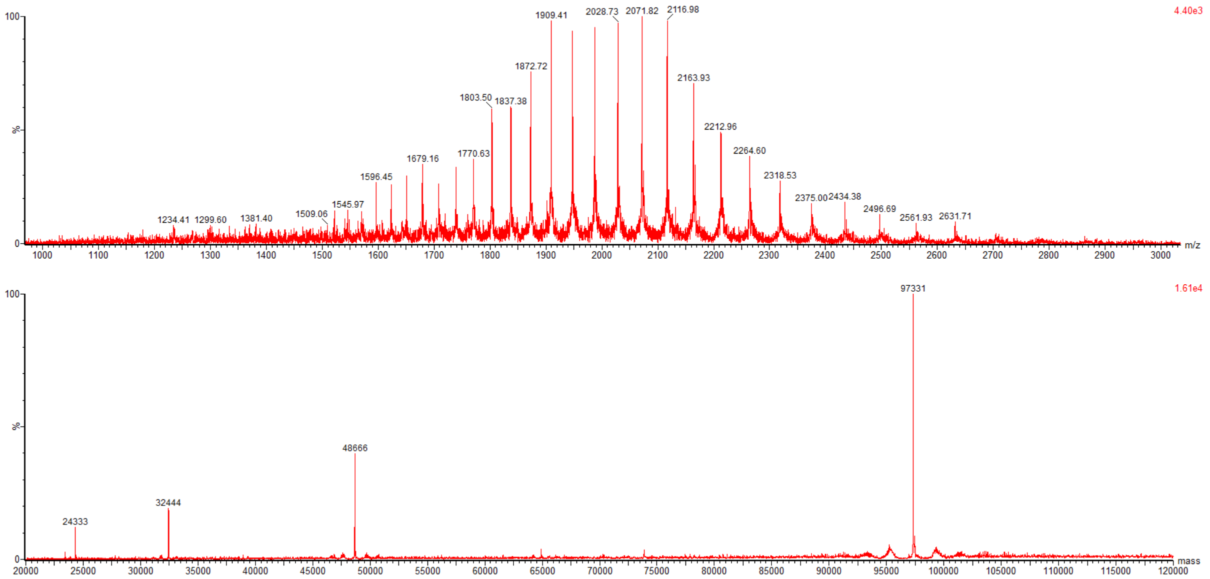

Figure S17: Non-deconvoluted (top) and deconvoluted (bottom) MS of conjugate **D5**. Expected 97333 Da, observed 97331 Da.

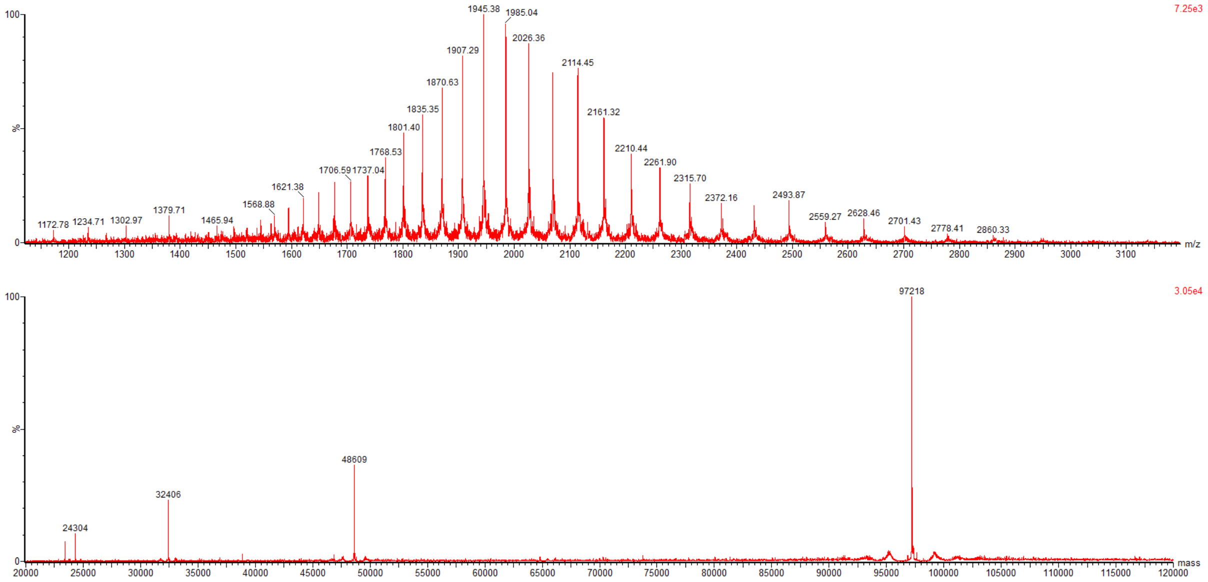

Figure S18: Non-deconvoluted (top) and deconvoluted (bottom) MS of conjugate **D6**. Expected 97220 Da, observed 97218 Da.

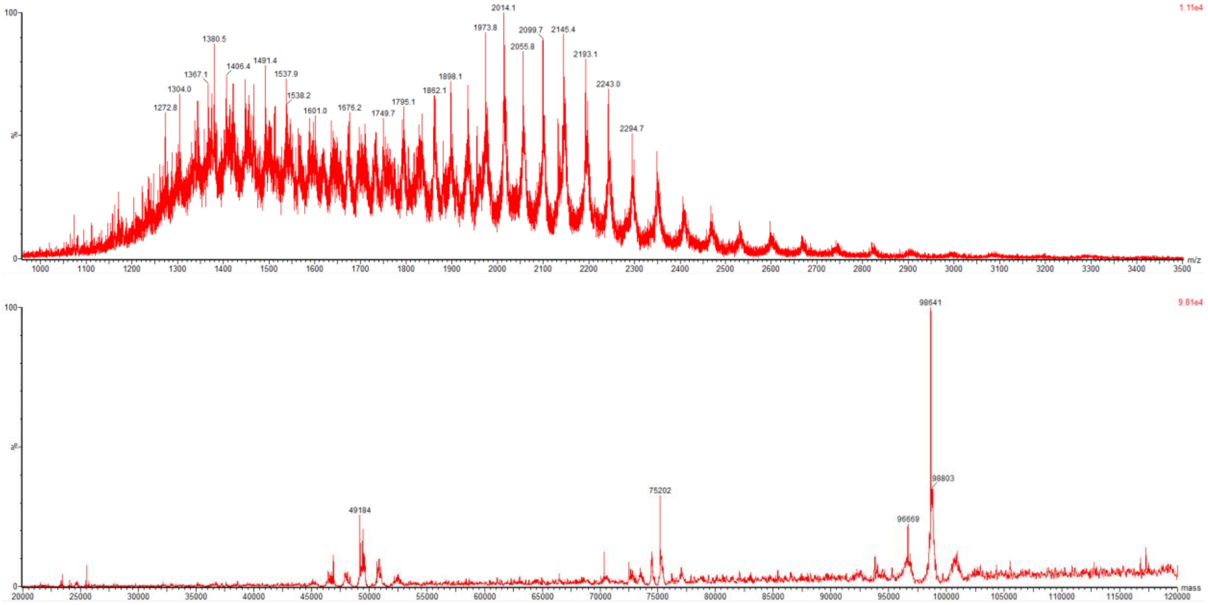

Figure S19: Non-deconvoluted (top) and deconvoluted (bottom) MS of conjugate **D3-AF488**. Expected 98631 Da, observed 98641 Da.

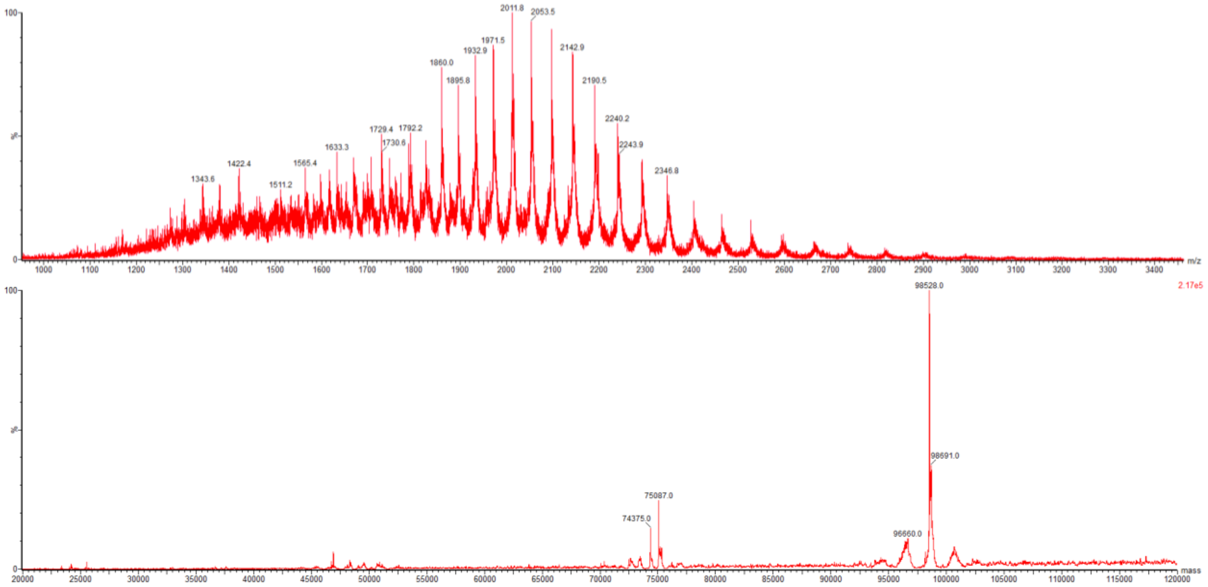

Figure S20: Non-deconvoluted (top) and deconvoluted (bottom) MS of conjugate **D4-AF488**. Expected 98519 Da, observed 98528 Da.

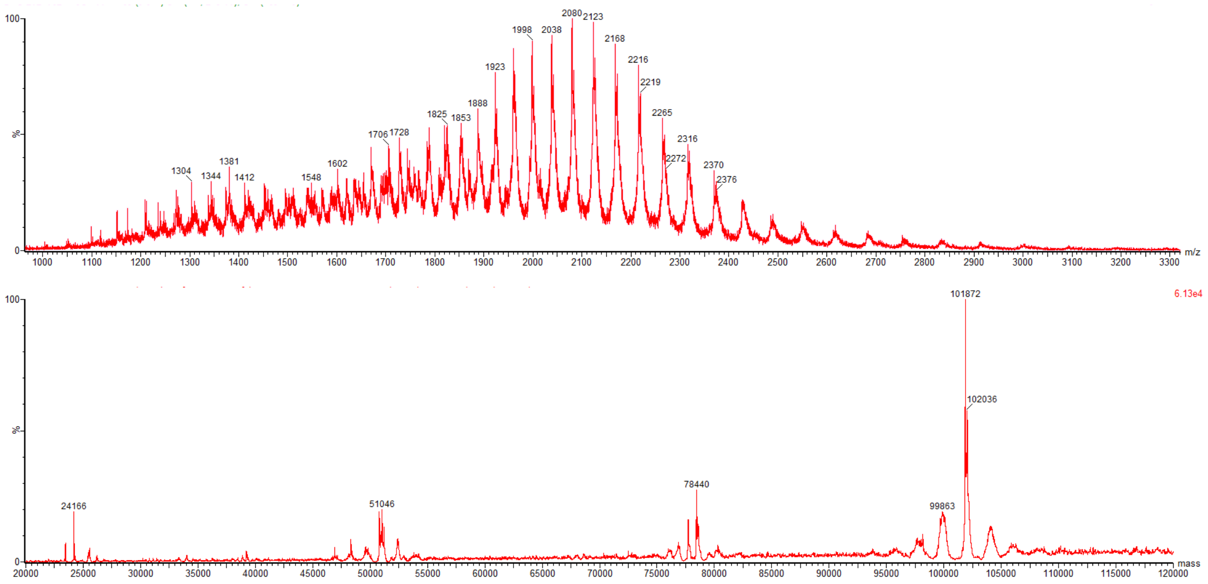

Figure S21: Non-deconvoluted (top) and deconvoluted (bottom) MS of conjugate **D3-MMAE**. Expected 101870 Da, observed 101872 Da.

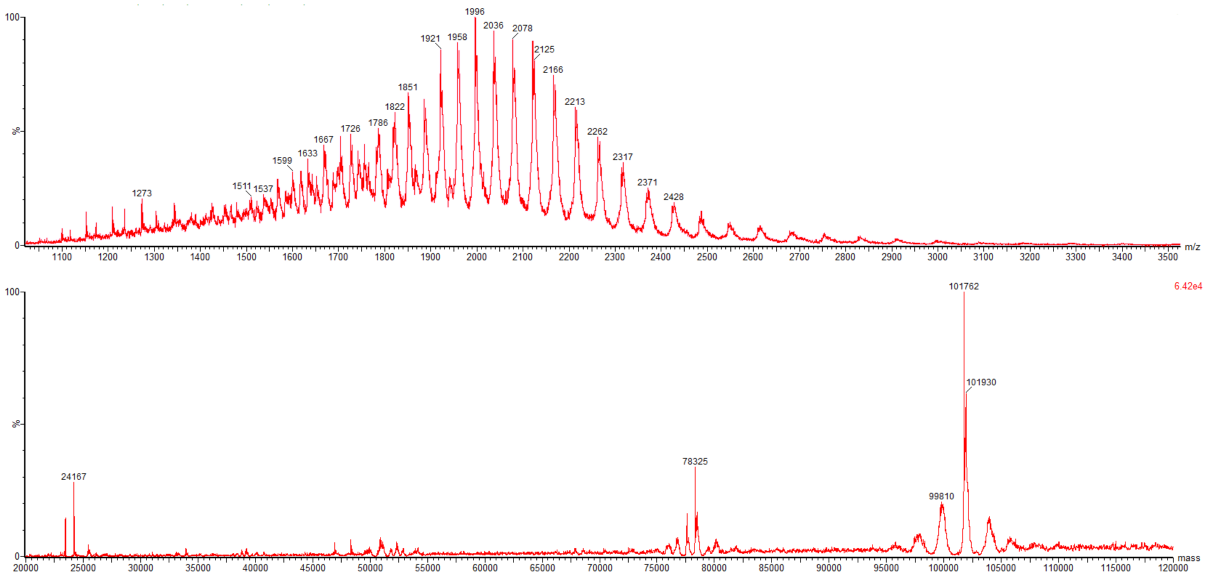

Figure S22: Non-deconvoluted (top) and deconvoluted (bottom) MS of conjugate **D4-MMAE**. Expected 101758 Da, observed 101762 Da.

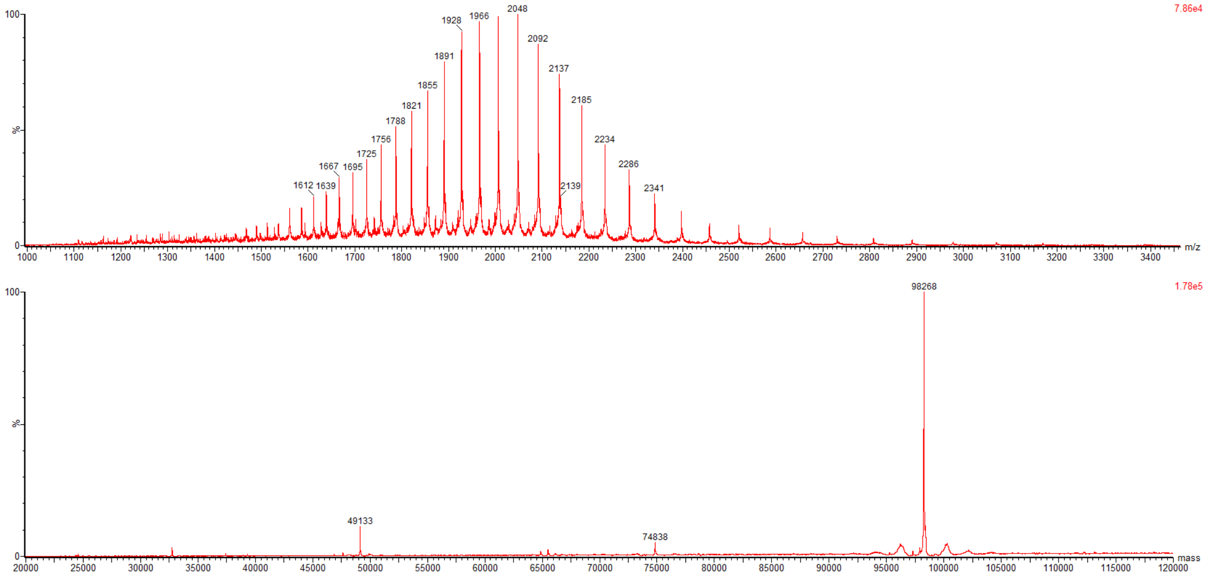

Figure S23: Non-deconvoluted (top) and deconvoluted (bottom) MS of conjugate **D5-TAMRA**. Expected 98269 Da, observed 98268 Da.

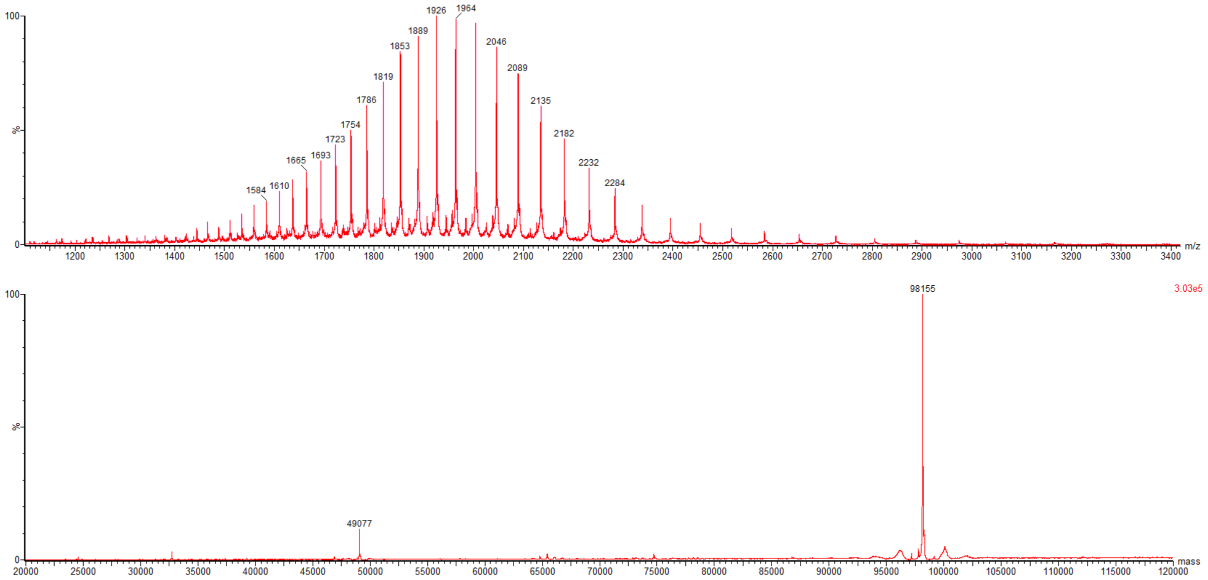

Figure S24: Non-deconvoluted (top) and deconvoluted (bottom) MS of conjugate **D6-TAMRA**. Expected 98156 Da, observed 98155 Da.

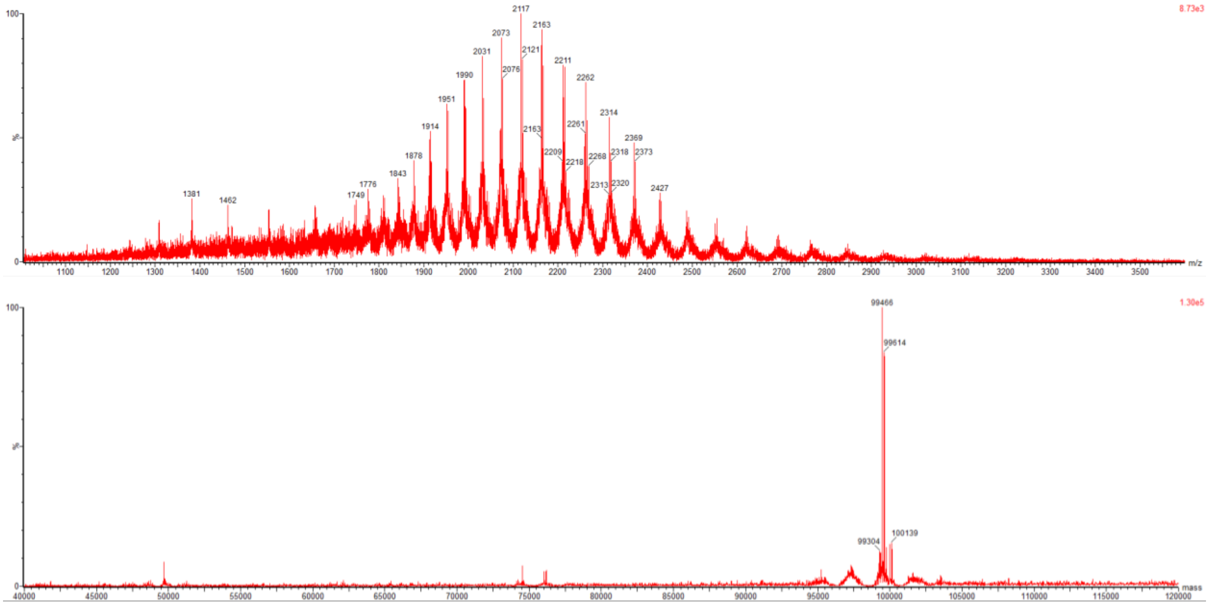

Figure S25: Non-deconvoluted (top) and deconvoluted (bottom) MS of conjugate **D7**. Expected 99466 and 99612 Da, observed 99466 and 99614 Da (glycosylation).

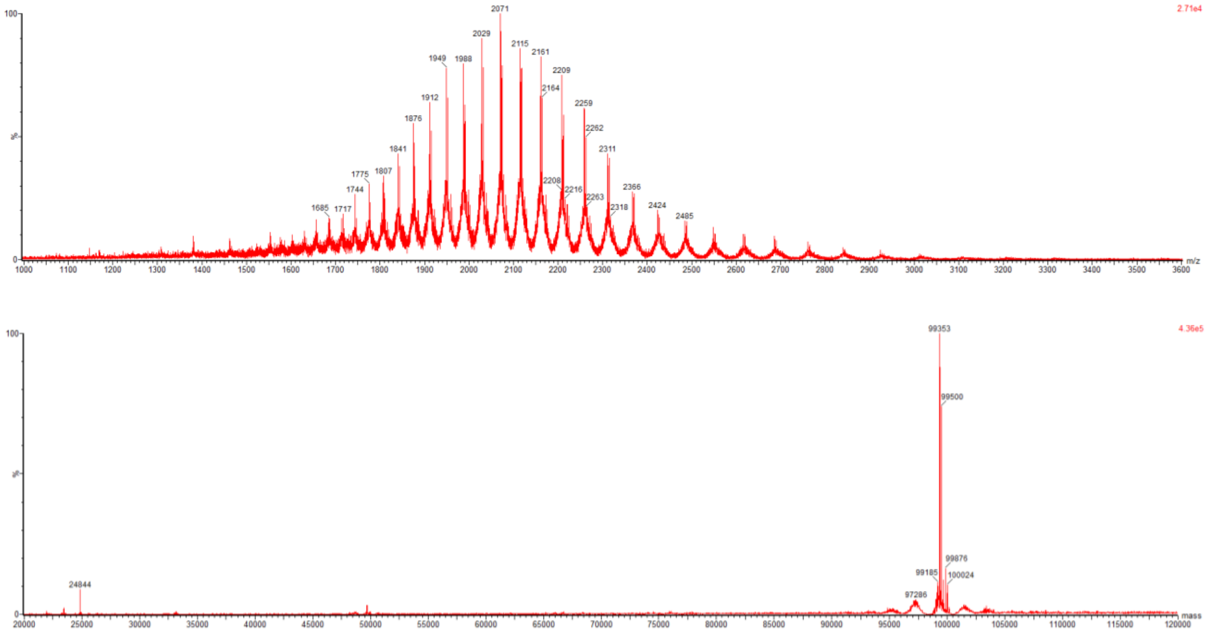

Figure S26: Non-deconvoluted (top) and deconvoluted (bottom) MS of conjugate **D8**. Expected 99349 and 99495 Da, observed 99353 and 99500 Da.

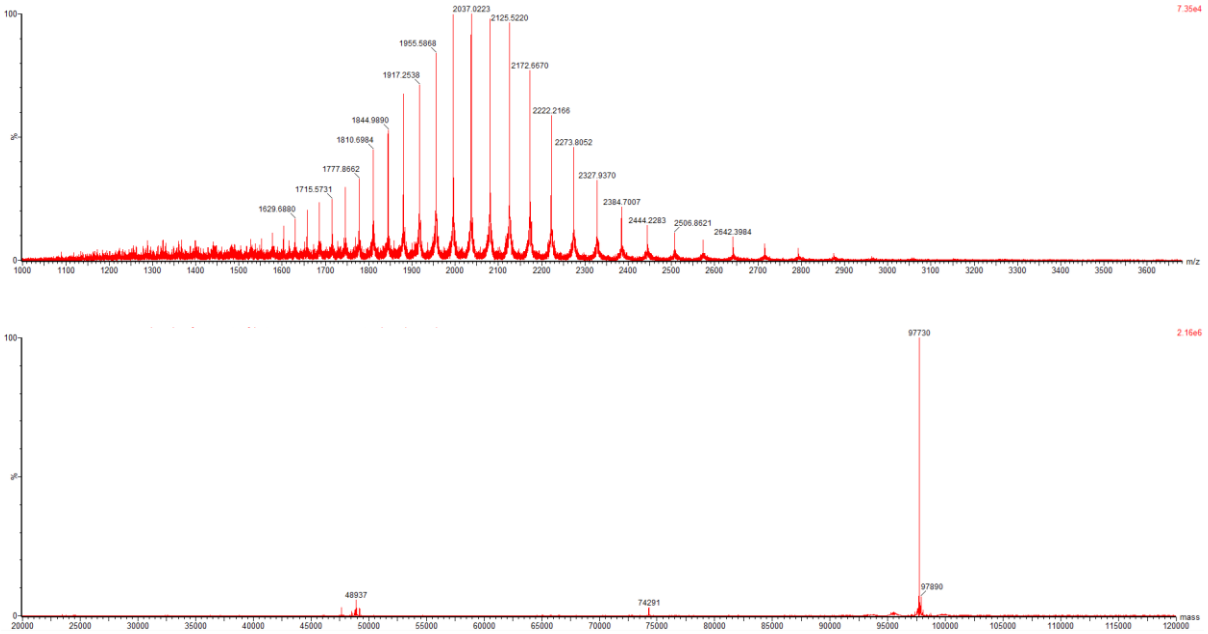

Figure S27: Non-deconvoluted (top) and deconvoluted (bottom) MS of conjugate **D9**. Expected 97724 Da, observed 97730 Da.

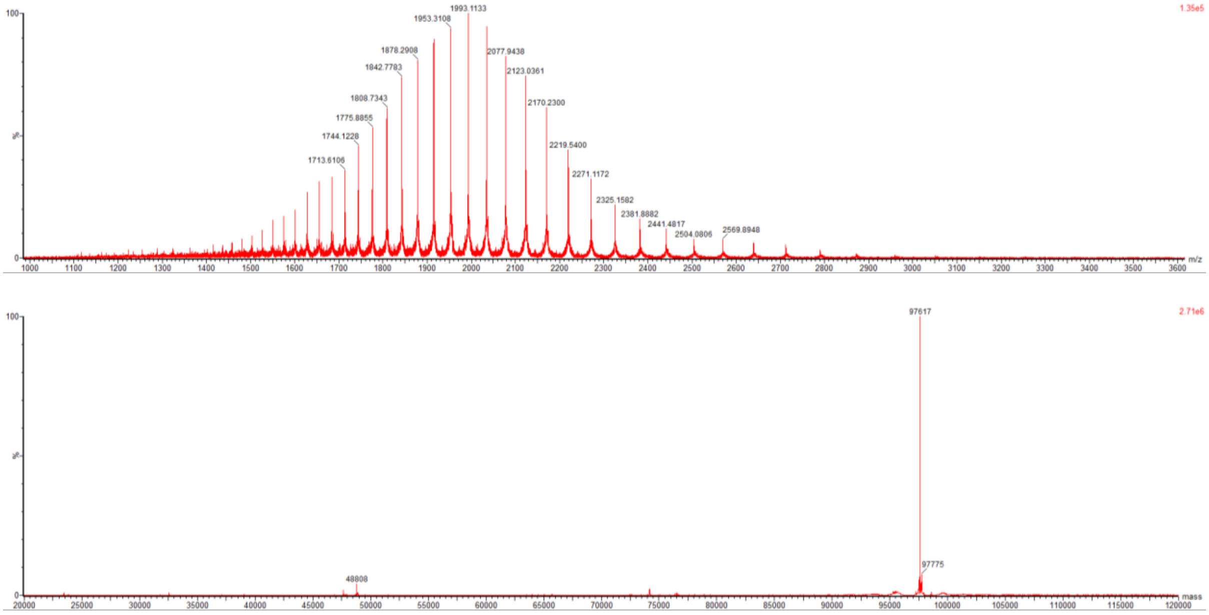

Figure S28: Non-deconvoluted (top) and deconvoluted (bottom) MS of conjugate **D10**. Expected 97611 Da, observed 97617 Da.

Bioconjugation Analyses

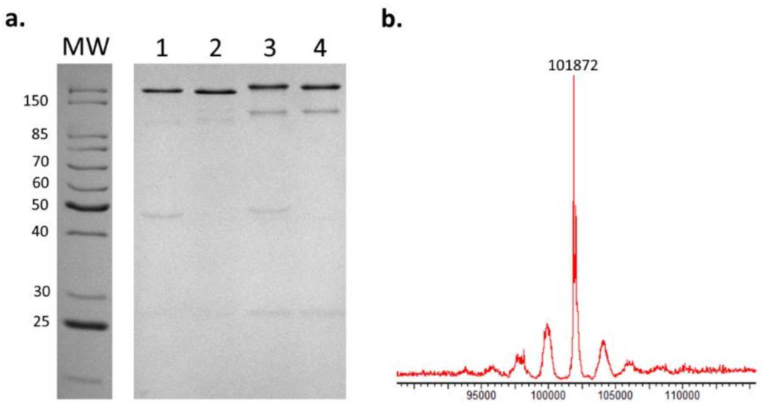

Figure S29. (a) SDS-PAGE analysis (Coomassie stain) of **D3-MMAE** and **D4-MMAE**. Full gel shown in Figure S5. Lanes: MW = molecular weight marker; 1 = **D3**; 2 = **D4**; 3 = **D3-MMAE**; 4 = **D4-MMAE**; (b) deconvoluted MS of **D3-MMAE**, observed 101872 Da, expected 101870 Da; and (c) deconvoluted MS of **D4-MMAE**, observed 101762 Da, expected 101758 Da.

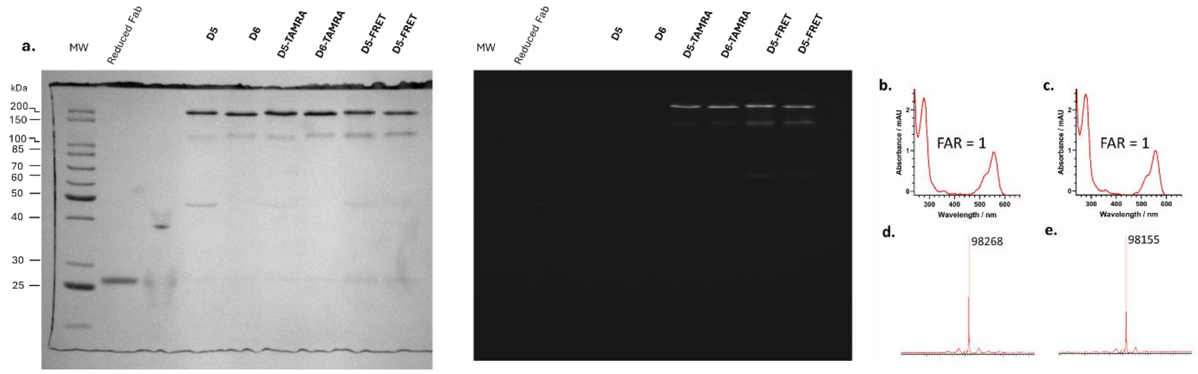

Figure S30. (a) SDS-PAGE analysis of **D5-TAMRA**, **D6-TAMRA**, **D5-FRET**, **D6-FRET**. Lanes: MW = molecular weight marker; Coomassie stain (left) and in-gel fluorescent (right) shown; (b) average UV-vis spectrum of **D5-TAMRA** (three independent measurements); (c) average UV-vis spectrum of **D6-TAMRA** (three independent measurements); (d) deconvoluted MS of **D5-TAMRA**, observed 98268 Da, expected 98269 Da; and (e) deconvoluted MS of **D6-TAMRA**, observed 98155 Da, 98156 expected Da.

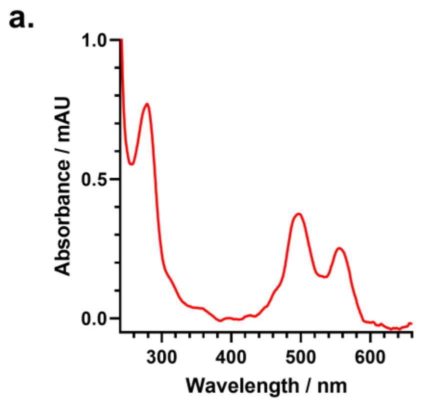

Figure S31. Average UV-vis spectra of (a) **D5-FRET** and (b) **D6-FRET** (three independent measurements).

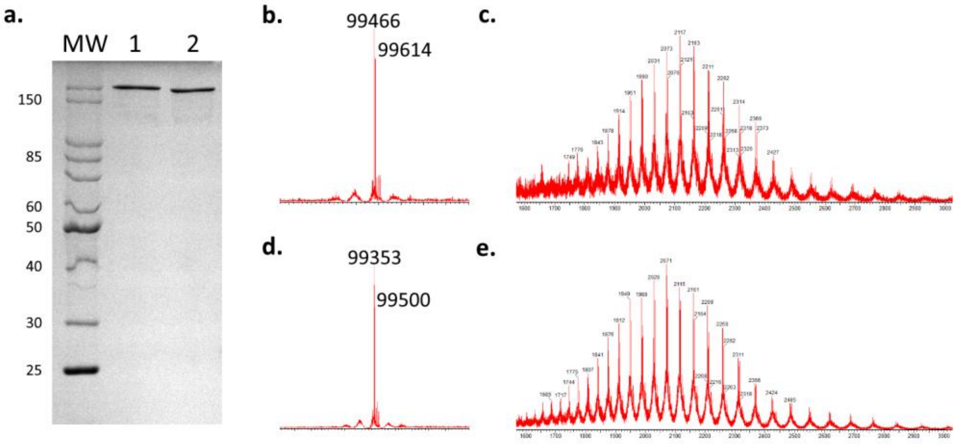

Figure S32. (a) SDS-PAGE analysis of α-EGFR×α-HER2 Fab dimers **D7** and **D8**. Lanes: MW = molecular weight marker; 1 = **D7**; 4 = **D8**. SDS-PAGE conditions: 12% SDS, 200V, 65 min; (b) deconvoluted and (c) non-deconvoluted MS of **D7** (observed 99466 and 99614 Da, expected 99462 and 99607 Da); (d) deconvoluted and (e) non-deconvoluted MS of **D8** (observed 99353 and 99500 Da, expected 99349 and 92494 Da).

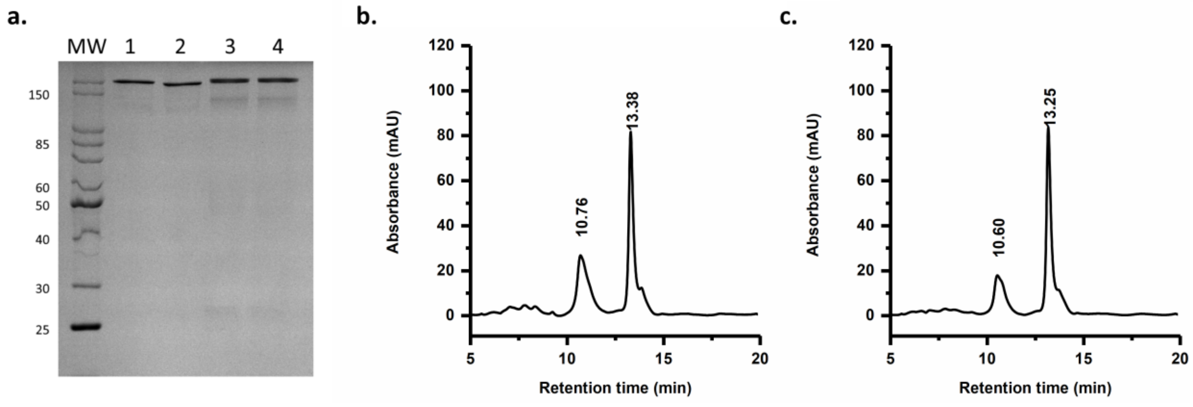

Figure S33. (a) SDS-PAGE analysis of α-EGFR×α-HER2 BisFab ADCs **D7-MMAE** and **D8-MMAE**. Lanes: MW = molecular weight marker; 1 = **D7**; 2 = **D8**; 3 = **D7-MMAE**; 4 = **D8-MMAE**. SDS-PAGE conditions: 12% SDS, 200V, 65 min. HIC analysis of (b) **D7-MMAE** and (c) **D8-MMAE**.

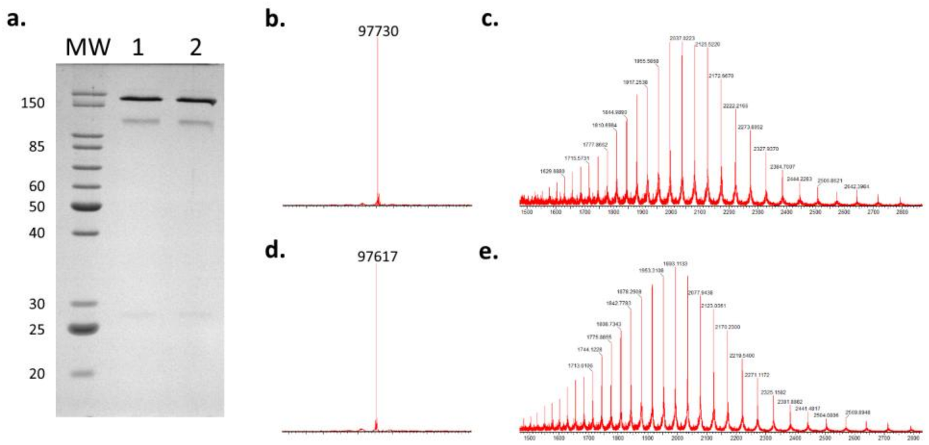

Figure S34. (a) SDS-PAGE analysis of α-PD-L1×α-HER2 Fab dimers **D9** and **D10**. Lanes: MW = molecular weight marker; 1 = **D9**; 4 = **D10**. SDS-PAGE conditions: 12% SDS, 200V, 65 min; (b) deconvoluted and (c) non-deconvoluted MS of **D9** (observed 97730 Da, expected 97724 Da); (d) deconvoluted and (e) non-deconvoluted MS of **D10** (observed 97617 Da, expected 97611 Da).

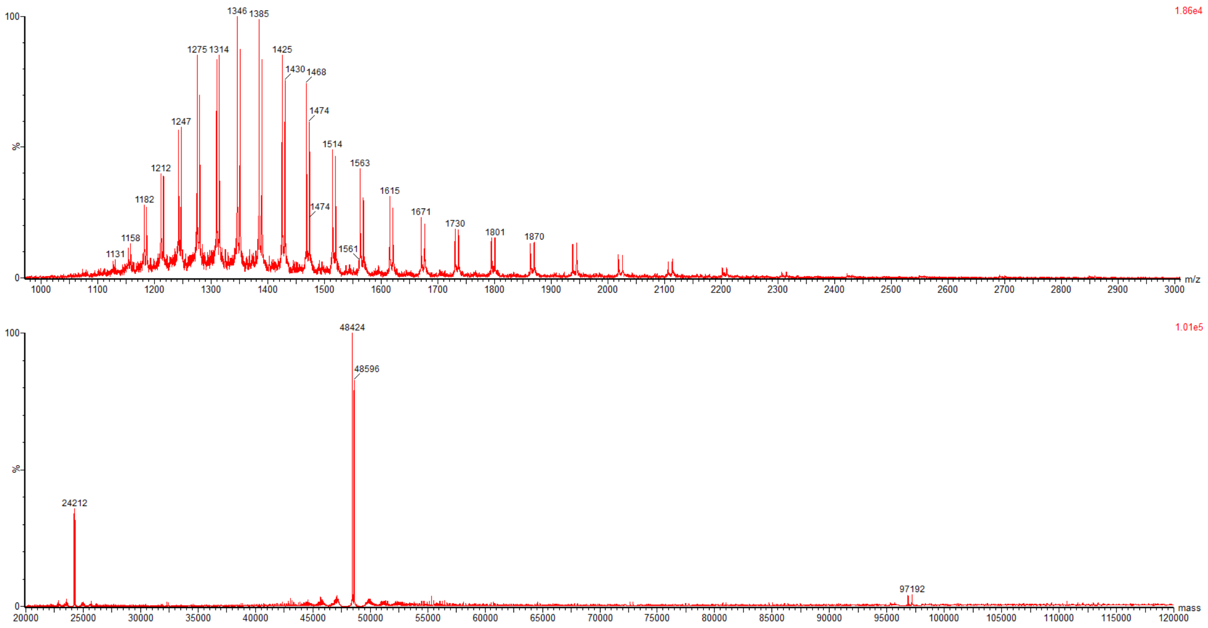

Figure S35. Non-deconvoluted (top) and deconvoluted (bottom) MS of conjugate **D1** after MMP2 cleavage. Observed 48424 Da and 48596 Da, expected 48421 Da and 48594 Da.

References

1 J. D. Bargh, S. J. Walsh, A. Isidro-Llobet, S. Omarjee, J. S. Carroll and D. R. Spring, *Chem. Sci.*, 2020, **11**, 2375–2380.

2 Y. Amano, N. Umezawa, S. Sato, H. Watanabe, T. Umehara and T. Higuchi, *Bioorg. Med. Chem.*, 2017, **25**, 1227–1234.

3 Y. H. Lau and D. R. Spring, *Synlett*, **2011**, 1917–1919.

4 L. H. Zhang, G. S. Kauffman, J. A. Pesti and J. Yin, *J. Org. Chem.*, 1997, **62**, 6918–6920.

5 N. Fischer, E. D. Goddard-Borger, R. Greiner, T. M. Klapötke, B. W. Skelton and J. Stierstorfer, *J. Org. Chem.*, 2012, **77**, 1760–1764.

6 S. C. Gill and P. H. von Hippel, *Anal. Biochem.*, 1989, **182**, 319–326.

7 S. J. Walsh, *The Development of Divinyl-Heteroaryl Linkers for the Synthesis of Stable and Functional Antibody-Drug Conjugates*, University of Cambridge, 2019.

8 P. Rassam, N. A. Copeland, O. Birkholz, C. Tóth, M. Chavent, A. L. Duncan, S. J. Cross, N. G. Housden, R. Kaminska, U. Seger, D. M. Quinn, T. J. Garrod, M. S. P. Sansom, J. Piehler, C. G. Baumann and C. Kleanthous, *Nature*, 2015, **523**, 333–336.

9 A. Pabbathi, L. Coleman, S. Godar, A. Paul, A. Garlapati, M. Spencer, J. Eller and J. D. Alper, *Biophys. J.*, 2022, **121**, 1715–1726.
